# A nickel phosphide nanoalloy catalyst for the C-3 alkylation of oxindoles with alcohols

**DOI:** 10.1038/s41598-021-89561-1

**Published:** 2021-05-21

**Authors:** Shu Fujita, Kohei Imagawa, Sho Yamaguchi, Jun Yamasaki, Seiji Yamazoe, Tomoo Mizugaki, Takato Mitsudome

**Affiliations:** 1 Department of Materials Engineering Science, Graduate School of Engineering Science, Osaka University, 1-3 Machikaneyama, Toyonaka, Osaka 560-8531 Japan; 2 Research Center for Ultra-High Voltage Electron Microscopy, Osaka University, 7-1, Mihogaoka, Ibaraki, Osaka 567-0047 Japan; 3 Department of Chemistry, Tokyo Metropolitan University, 1-1 Minami Osawa, Hachioji, Tokyo 192-0397 Japan; 4 Innovative Catalysis Science Division, Institute for Open and Transdisciplinary Research Initiatives (ICS-OTRI), Osaka University, Suita, Osaka 565-0871 Japan

**Keywords:** Heterogeneous catalysis, Green chemistry

## Abstract

Although transition metal phosphides are well studied as electrocatalysts and hydrotreating catalysts, the application of metal phosphides in organic synthesis is rare, and cooperative catalysis between metal phosphides and supports remains unexplored. Herein, we report that a cerium dioxide-supported nickel phosphide nanoalloy (nano-Ni_2_P/CeO_2_) efficiently promoted the C-3 alkylation of oxindoles with alcohols without any additives through the borrowing hydrogen methodology. Oxindoles were alkylated with various alcohols to provide the corresponding C-3 alkylated oxindoles in high yields. This is the first catalytic system for the C-3 alkylation of oxindoles with alcohols using a non-precious metal-based heterogeneous catalyst. The catalytic activity of nano-Ni_2_P/CeO_2_ was comparable to that reported for precious metal-based catalysts. Moreover, nano-Ni_2_P/CeO_2_ was easily recoverable and reusable without any significant loss of activity. Control experiments revealed that the Ni_2_P nanoalloy and the CeO_2_ support functioned cooperatively, leading to a high catalytic performance.

## Introduction

Metal–metal nanoalloys have been recognized as key materials for the development of novel catalysts. In contrast, metal–nonmetal nanoalloys have not been widely studied in the field of fine chemical synthesis. In this context, metal phosphides have recently received growing attention as electrocatalysts for the hydrogen evolution reaction^1–3^ and hydrodesulfurization catalysts in the petroleum industry^4–6^ due to the fact that they exhibit unique catalysis derived from the charge transfer effect of metal to phosphorus^7,8^ and the ensemble effect^9,10^. Despite these fascinating properties, the application of metal phosphides in liquid-phase organic synthesis is rare^11–19^, with the majority of reported reactions to date being simple hydrogenation reactions^20–47^. Therefore, the study of metal phosphide catalysis for organic synthesis remains an exciting and unexplored research area. Furthermore, although supports are known to greatly improve catalytic performances, cooperative catalysis between metal phosphides and supports has yet to be comprehensively explored. Therefore, functionalization by combining a support and a metal phosphide catalyst is expected to lead to new metal phosphide catalysts for other organic transformations.

**Scheme 1:**
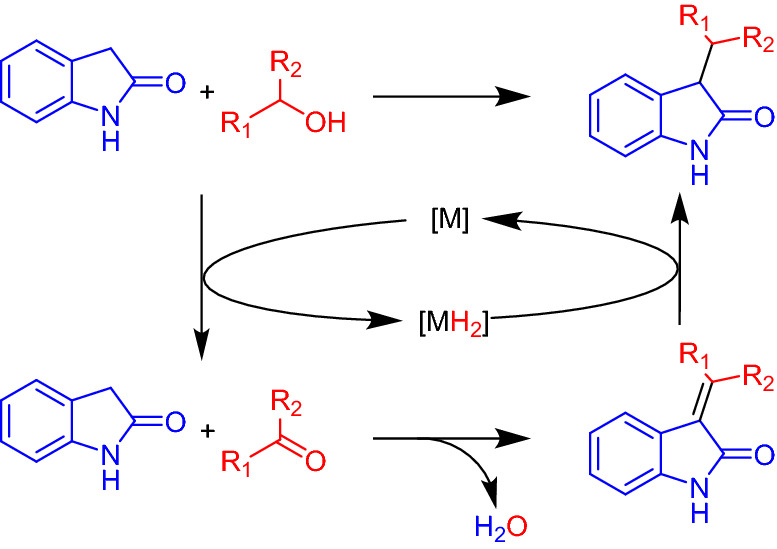
C-3 alkylation of oxindole with alcohols through the borrowing hydrogen methodology.

The C-3 alkylation of oxindoles is one of the key routes to the synthesis of functionalized oxindoles that possess significant potential for use in a wide range of biological applications, including as NMDA antagonists^48^, antiangiogenic agents^49^, and anti-cancer drugs^50^. Recently, catalytic methods using alcohols as alkylating reagents have attracted attention for the C-3 alkylation of oxindoles because this reaction proceeds through the borrowing hydrogen (BH) methodology with the co-production of only water, thereby providing a high atom efficiency (Scheme 1)^51^. In addition, various metal complex catalysts have been reported for the alkylation reaction with alcohols^50–63^. However, these catalysts inevitably require complex ligands and the addition of strong bases. Furthermore, difficulties in the separation and reuse of these catalysts remain an ongoing issue. As an alternative, reusable heterogeneous catalysts based on precious metals have been developed for the C-3 alkylation reaction^64–66^. Although these catalysts are effective, they are both expensive and rare. In terms of non-precious metal-based heterogeneous catalysts, only Raney Ni has been reported to date^67,68^. However, a large amount of Ni is required to promote the alkylation, and the turnover number (TON) tends to be low (i.e., < 0.6), thereby indicating that this system possesses an inadequate catalytic efficiency. Therefore, the development of highly active and reusable non-precious metal-based catalysts for the C-3 alkylation of oxindoles with alcohols remains a great challenge.

We herein report the preparation and application of a cerium dioxide-supported nickel phosphide nanoalloy (nano-Ni_2_P/CeO_2_) for the synthesis of C-3 functionalized oxindoles using alcohols through the BH methodology without the need for additives. This constitutes the first catalytic system for the synthesis of C-3 functionalized oxindoles using a non-precious metal-based heterogeneous catalyst. Furthermore, the recovery and reuse of nano-Ni_2_P/CeO_2_ are also evaluated.

## Results and discussion

**Figure 1:**
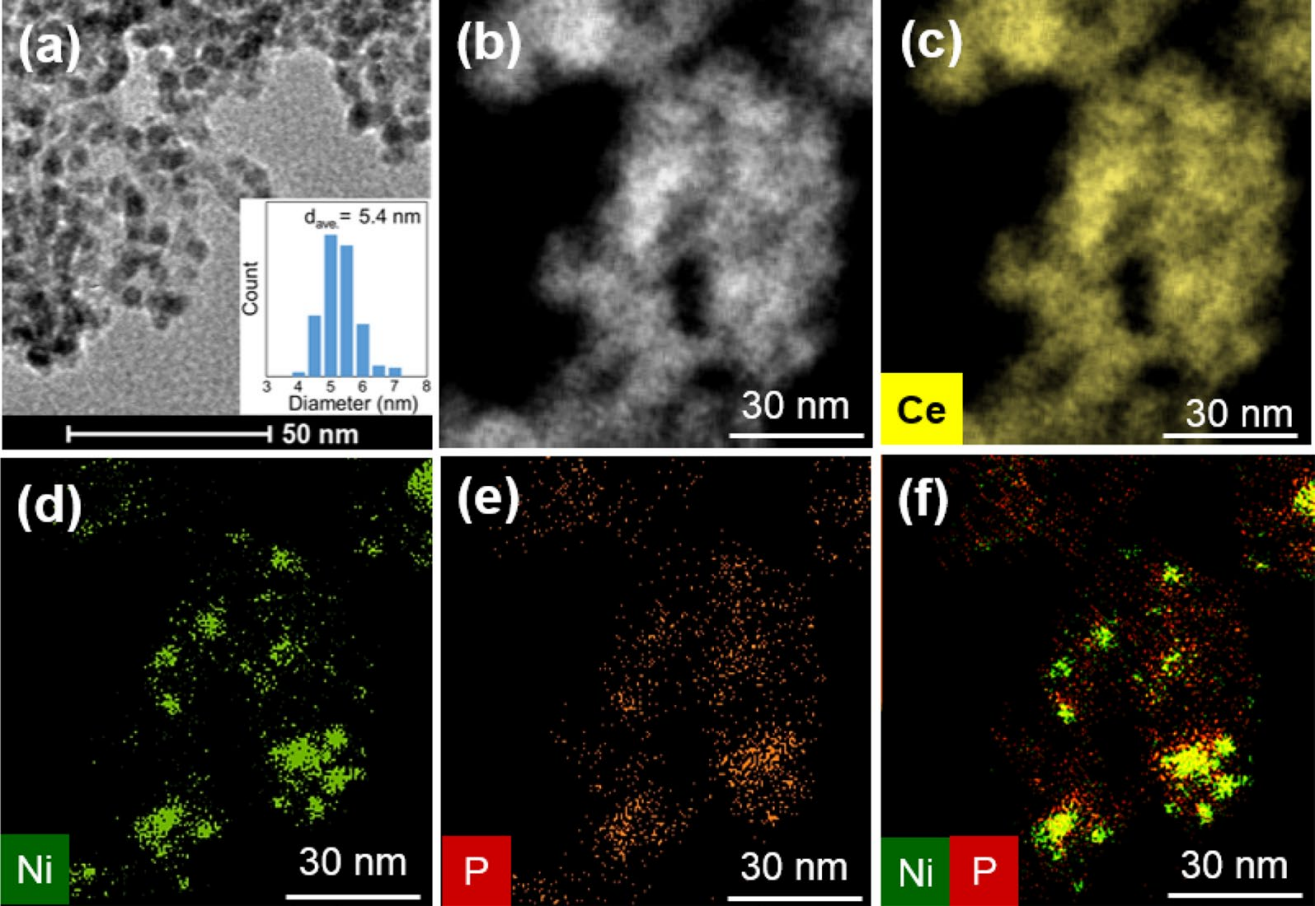
(**a**) TEM image of nano-Ni_2_P (the inset shows the histogram of nano-Ni_2_P). (**b**) HAADF-STEM image of nano-Ni_2_P/CeO_2_. Elemental mapping of (**c**) Ce, (**d**) Ni, and (**e**) P, and (**f**) a composite overlay of (**d**,**e**).

The desired nano-Ni_2_P was prepared according to our previous report with some modifications (see Supplementary Information for details)^43^. More specifically, NiCl_2_·6H_2_O was added to hexadecylamine in the presence of triphenylphosphite. The mixture was heated at 120 °C in vacuo, and then the temperature was increased to 320 °C under Ar atmosphere. The precipitate was collected by centrifugation and washed with acetone and chloroform to afford the nano-Ni_2_P. Subsequently, this nano-Ni_2_P was dispersed in hexane and stirred with CeO_2_, yielding the desired nano-Ni_2_P/CeO_2_ (Fig. S1). BET surface area of the transmission electron microscopy (TEM) images of the nano-Ni_2_P/support catalysts are shown in Table S1 and Fig. S2, respectively. The formation of nano-Ni_2_P was confirmed by X-ray diffraction (XRD) measurements, whereby the diffraction peaks located at 2*θ* = 40.8, 44.7, 47.3, and 54.1° were attributed to the (111), (201), (210), and (300) planes of Ni_2_P (JCPDS card no. 03-0953), respectively (Fig. S3). A representative TEM image of nano-Ni_2_P revealed a collection of spherical nanoparticles with a mean diameter of 5.4 nm (Fig. 1a). The elemental distributions of Ni and P on CeO_2_ were determined using high-angle annular dark field scanning transmission electron microscopy (HAADF-STEM) coupled with energy dispersive X-ray spectroscopy (EDX; Fig. 1b–f). It was found that nano-Ni_2_P was highly dispersed on the CeO_2_ support, in which Ni and P were homogeneously distributed. These results demonstrate that nano-Ni_2_P is uniformly immobilized on the surface of CeO_2_. Furthermore, the Ni *K*-edge X-ray absorption near edge structure (XANES) spectrum shows that the absorption edge energy of nano-Ni_2_P/CeO_2_ is close to that of Ni foil, suggesting that the Ni species in nano-Ni_2_P/CeO_2_ possesses metallic states (Fig. S4). This conclusion is also supported by the result of the XPS analysis of nano-Ni_2_P/CeO_2_, where two peaks observed at 852.7 and 869.9 eV are similar to those of metallic Ni 2p_3/2_ (852.8 eV) and Ni 2p_1/2_ (870.0 eV), respectively (Fig. S5).

Initially, the catalytic potential of the nano-Ni_2_P/support catalysts for the C-3 alkylation of oxindole with benzyl alcohol was investigated at 140 °C for 10 h in toluene (Table 1). Notably, nano-Ni_2_P/CeO_2_ exhibited a high catalytic activity to provide 3-benzyl-2-oxindole (**1a**) in 95% yield (entry 1). In contrast, nano-Ni_2_P/TiO_2_ and nano-Ni_2_P/SiO_2_ gave low yields of **1a**, accompanied by the production of 3-benzylideneoxindole (**2a**) in ca. 30% yield (entries 2 and 3). Furthermore, nano-Ni_2_P immobilized on other supports such as Al_2_O_3_, hydrotalcite (HT, Mg_6_Al_2_(OH)_16_CO_3_·4H_2_O), MgO, Nb_2_O_5_, and ZnO showed almost no activity for the C-3 alkylation of oxindole (entries 4–8), indicating that modulation of the support significantly affects the catalytic performance of nano-Ni_2_P for the alkylation reaction. For comparison with nano-Ni_2_P/CeO_2,_ Ni/CeO_2_ was prepared via the impregnation method, and H_2_-treated Ni/CeO_2_ (Ni/CeO_2_-Red) was also synthesized. These species were subjected to testing as model catalysts for the conventional Ni nanoparticles of NiO and Ni(0), respectively, in the C-3 alkylation of oxindole with benzyl alcohol (see Supplementary Information for details regarding catalyst preparation). However, in sharp contrast to the highly active nano-Ni_2_P/CeO_2_, these conventional nickel nanoparticle catalysts exhibited very low activities (entries 9 and 10). These results clearly demonstrate the importance of the combination of nano-Ni_2_P with a CeO_2_ support for efficiently promoting the alkylation of oxindole.

**Table 1:** C-3 alkylation of oxindole with benzyl alcohol using Ni catalysts^a^.


Entry	Catalyst	Conv. (%)	Yield (%)^b^	
			**1a**	**2a**
1	Nano-Ni_2_P/CeO_2_	> 99	95	4
2	Nano-Ni_2_P/TiO_2_	51	12	26
3	Nano-Ni_2_P/SiO_2_	33	3	29
4	Nano-Ni_2_P/Al_2_O_3_	3	1	1
5	Nano-Ni_2_P/HT	3	0	1
6	Nano-Ni_2_P/MgO	2	0	0
7	Nano-Ni_2_P/Nb_2_O_5_	5	0	0
8	Nano-Ni_2_P/ZnO	4	0	0
9	Ni/CeO_2_	30	14	10
10	Ni/CeO_2_-Red	9	6	3

^a^Reaction conditions: catalyst (0.15 g, 5 mol% Ni), oxindole (0.5 mmol), benzyl alcohol (1 mmol), toluene (2 mL), 140 °C, 10 h, N_2_ atmosphere.

^b^Yields based on oxindole were determined by gas chromatography-mass spectrometry (GC-MS) using naphthalene as an internal standard.

**Scheme 2:**
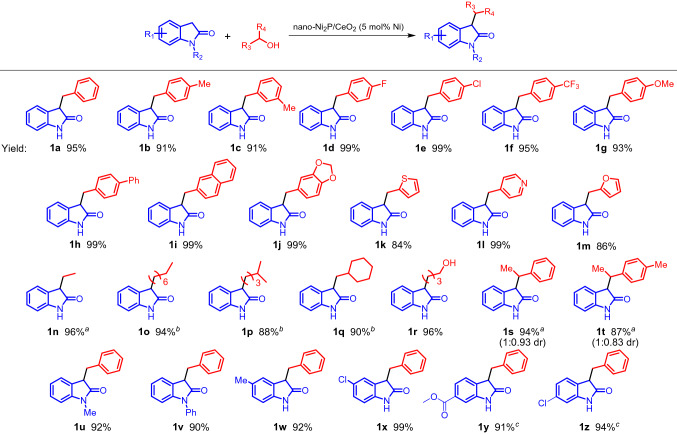
Substrate scope of the C-3 alkylation of oxindoles with alcohols catalyzed by nano-Ni_2_P/CeO_2_. Reaction conditions: nano-Ni_2_P/CeO_2_ (0.15 g, 5 mol% Ni), oxindole (0.5 mmol), alcohol (1 mmol), toluene (2 mL), 140 °C, 10 h, N_2_ atmosphere. Yields based on oxindole were determined by GC–MS using naphthalene as an internal standard. ^a^Alcohol (5 mmol), 180 °C, 24 h. ^b^Alcohol (5 mmol), 160 °C, 24 h. ^c^160 °C, 24 h.

With the nano-Ni_2_P/CeO_2_ in hand, we explored the substrate scope in the C-3 alkylation of oxindoles with alcohols (Scheme 2). Benzyl alcohols substituted with electron-withdrawing or electron-donating groups, such as methyl, halogen, trifluoromethyl, methoxy, and phenyl groups, were reacted with oxindole to afford the corresponding mono-C-3 alkylated oxindoles in high yields (**1a–1j**). It was also found that nano-Ni_2_P/CeO_2_ promoted the C-3 alkylation of oxindole with heterocyclic alcohols, such as 2-thiophenemethanol and 4-pylidinemethanol, although nitrogen or sulfur atoms often coordinate strongly to the metals, resulting in catalyst deactivation (**1k** and **1l**)^69,70^. Furfuryl alcohol, which is an important biomass-derived chemical alternative to petroleum-based chemicals, also acted as a good alkylation reagent to provide the corresponding C-3 alkylated oxindole (**1m**)^71^. Less active aliphatic alcohols (**1n–1r**) and secondary alcohols (**1s** and **1t**) could be applied to this catalytic system, giving the corresponding products in high yields. Notably, when 1,4-butanediol was used as the alkylating agent, mono-C3-alkylated oxindole was selectively obtained without the formation of di-alkylated products (**1r**). The excellent performance of nano-Ni_2_P/CeO_2_ was also demonstrated in the C-3 alkylation using 1-phenylethanol and 1-(*p*-tolyl)ethanol, which are challenging reagents due to their steric hindrances (**1s** and **1t**)^61^. Various oxindoles such as 1-methyloxindole, 1-phenyloxindole, 5-methyloxindole, 5-chlorooxindole, methyl-2-oxoindole-6-carboxylate, and 6-chlorooxindole, were also alkylated with benzyl alcohol to provide the desired products in > 90% yields (**1u**–**1z**). The above results therefore demonstrate that nano-Ni_2_P/CeO_2_ functions as a highly active catalyst for the C-3 alkylation of oxindoles with a wide range of alcohols.

**Scheme 3:**
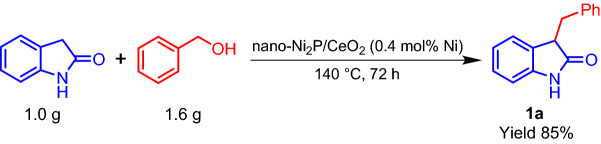
The gram-scale C-3 alkylation of oxindole with benzyl alcohol catalyzed by nano-Ni_2_P/CeO_2_. Reaction conditions: nano-Ni_2_P/CeO_2_ (0.4 mol% Ni), oxindole (1.0 g; 7.5 mmol), benzyl alcohol (1.6 g; 15 mmol), 140 °C, 72 h, N_2_ atmosphere. The yield based on oxindole was determined by GC–MS using naphthalene as an internal standard.

Furthermore, Ni_2_P/CeO_2_ was found to be applicable to a gram-scale reaction, with 1.0 g of oxindole being converted into **1a** in 85% yield, where the TON reached 212 (Scheme 3). This TON is greater than those of previously reported precious metal-based heterogeneous catalyst systems (Table S2), indicating the high performance of nano-Ni_2_P/CeO_2_ compared to those of the precious metal catalysts.

**Figure 2:**
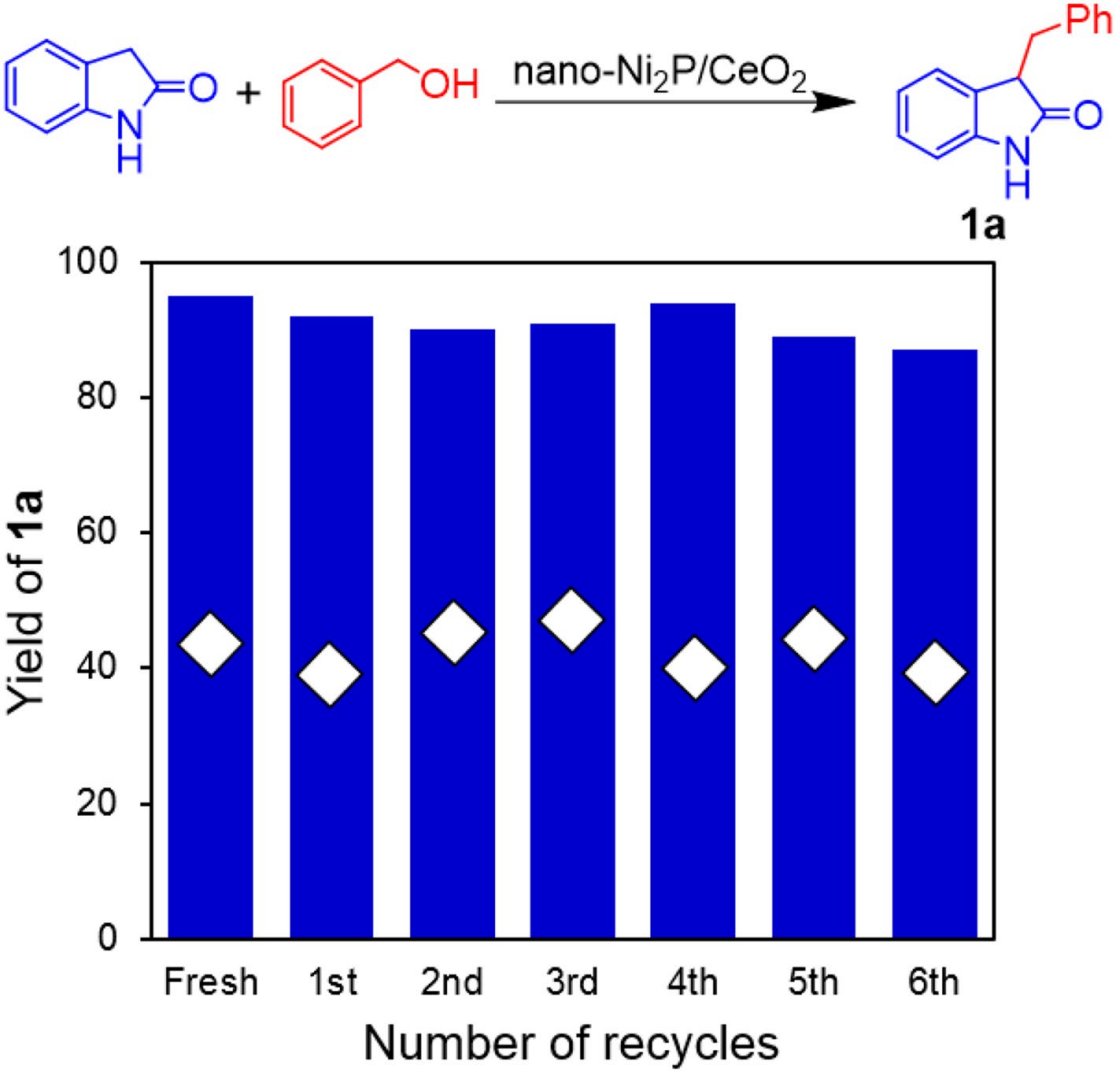
Nano-Ni_2_P/CeO_2_ recycling experiments for the C-3 alkylation of oxindole with benzyl alcohol. Reaction conditions: nano-Ni_2_P/CeO_2_ (0.15 g; 5 mol% Ni), oxindole (0.5 mmol), benzyl alcohol (1 mmol), toluene (2 mL), 140 °C, N_2_ atmosphere. Reaction time: 10 h (blue bars), 4 h (open diamond). Yields based on oxindole were determined by GC–MS using naphthalene as an internal standard.

Subsequently, the durability of nano-Ni_2_P/CeO_2_ was assessed through recycling experiments. After the alkylation of oxindole with benzyl alcohol, nano-Ni_2_P/CeO_2_ was easily recovered from the reaction mixture by centrifugation, and was used again in the following run without any pre-treatment (Fig. 2). Indeed, nano-Ni_2_P/CeO_2_ provided a high yield of **1a** even after the 6th recycling experiment. We further investigated the reaction rate at an incomplete reaction time (4 h), and found that **1a** (open diamond in Fig. 2) was obtained in similar yields using reused and fresh nano-Ni_2_P/CeO_2_, thereby demonstrating the excellent reusability of this catalyst. Inductively coupled plasma atomic emission spectrometry (ICP-AES) analysis revealed that the concentration of Ni in the solution after the alkylation was below the detection limit (0.1 ppm), and elemental analysis of the nano-Ni_2_P/CeO_2_ showed that the Ni content of catalyst did not change following the reaction (Table S3). These results clearly demonstrate the high durability of nano-Ni_2_P/CeO_2_ in the C-3 alkylation reactions of oxindole.

**Figure 3:**
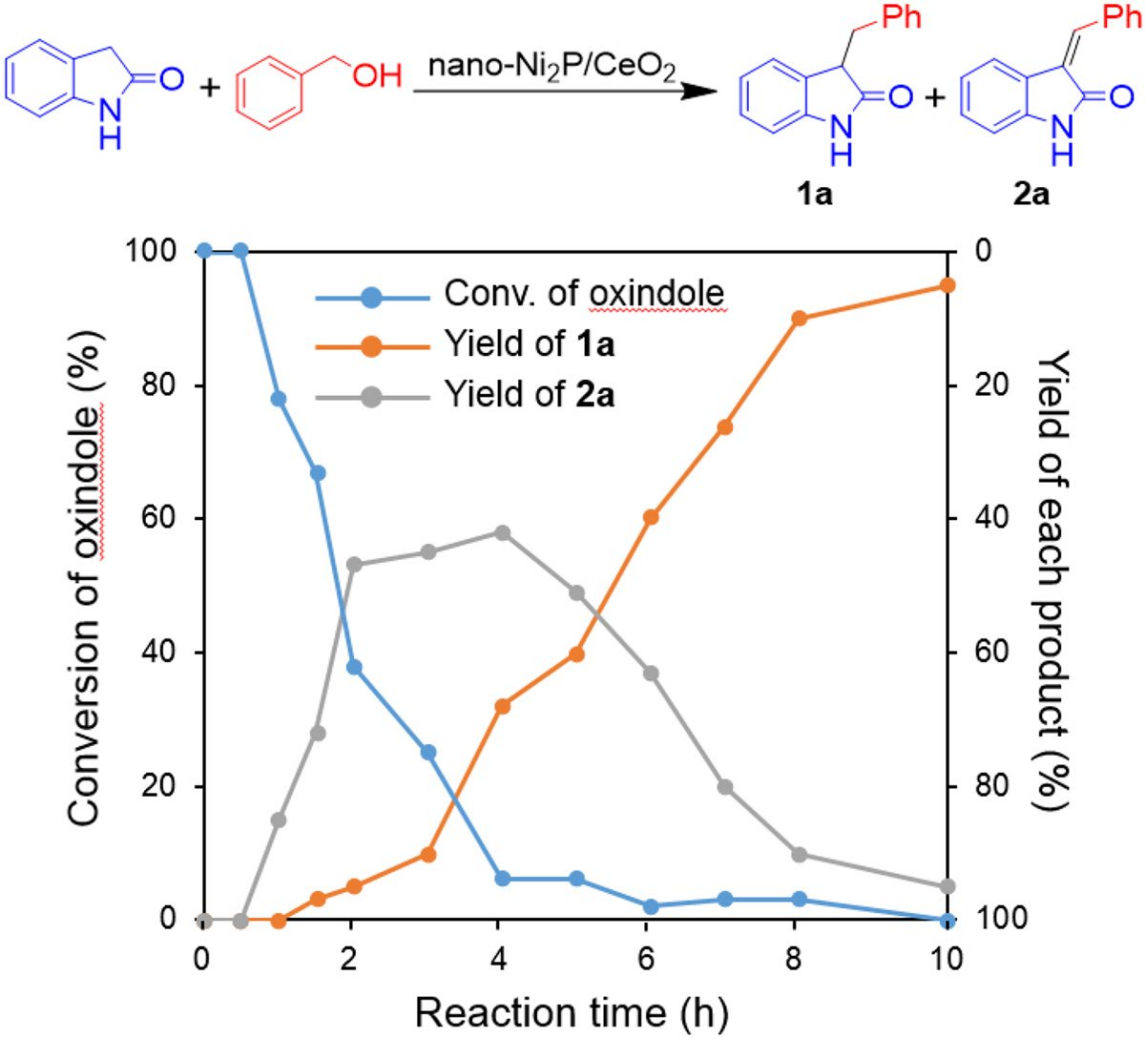
Reaction profile for the C-3 alkylation of oxindole with benzyl alcohol catalyzed by nano-Ni_2_P/CeO_2_. Reaction conditions: nano-Ni_2_P/CeO_2_ (5 mol% Ni), oxindole (0.5 mmol), benzyl alcohol (1 mmol), toluene (2 mL), 140 °C, N_2_ atmosphere. The conversions and yields were calculated based on oxindole.

**Figure 4:**
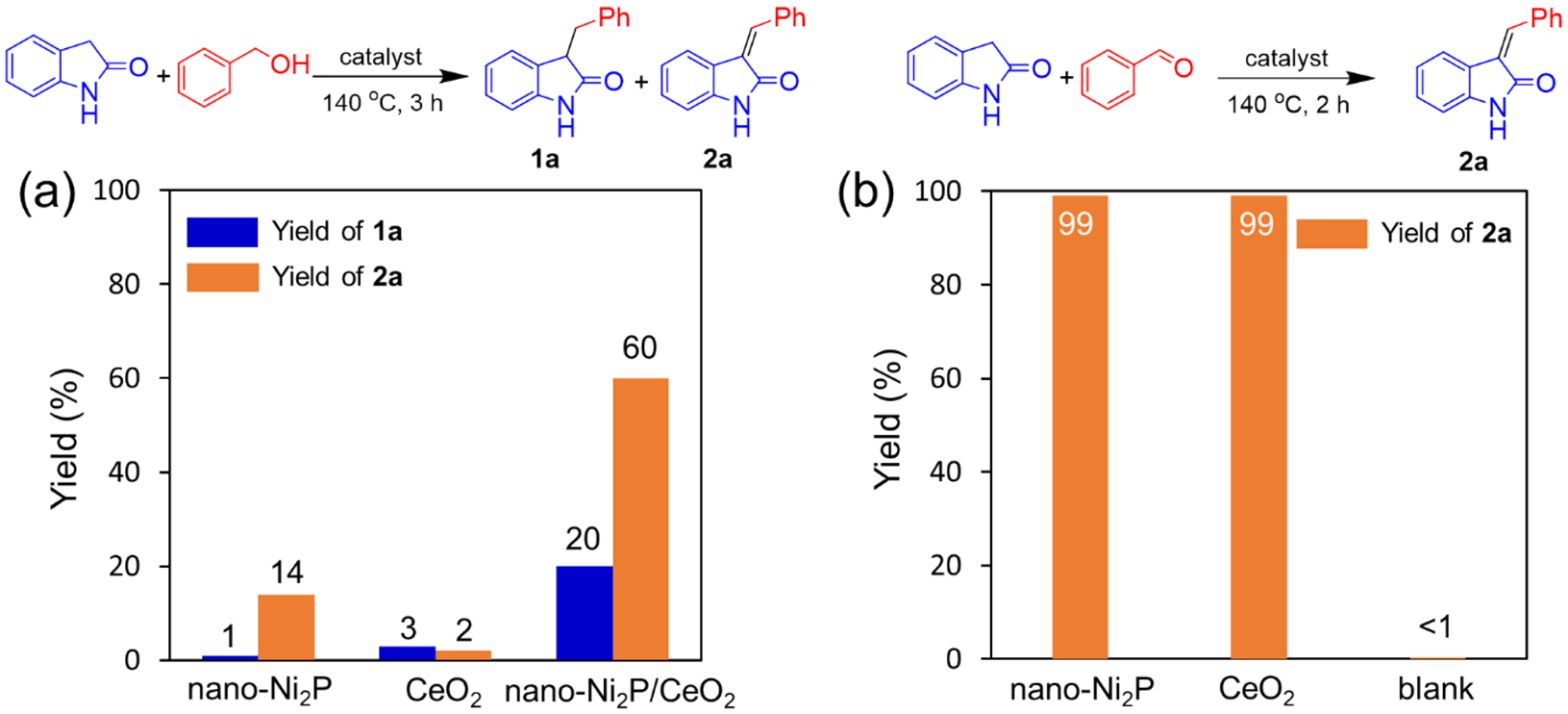
Control experiments for (**a**) the C-3 alkylation of oxindole with benzyl alcohol, and (**b**) the aldol-type condensation of oxindole with benzaldehyde. Reaction conditions: nano-Ni_2_P (4.3 mg), CeO_2_ or nano-Ni_2_P/CeO_2_ (0.15 g), oxindole (0.5 mmol), benzyl alcohol or benzaldehyde (1 mmol), toluene (2 mL), N_2_ atmosphere. Yields based on oxindole were determined by GC–MS using naphthalene as an internal standard.

**Scheme 4:**
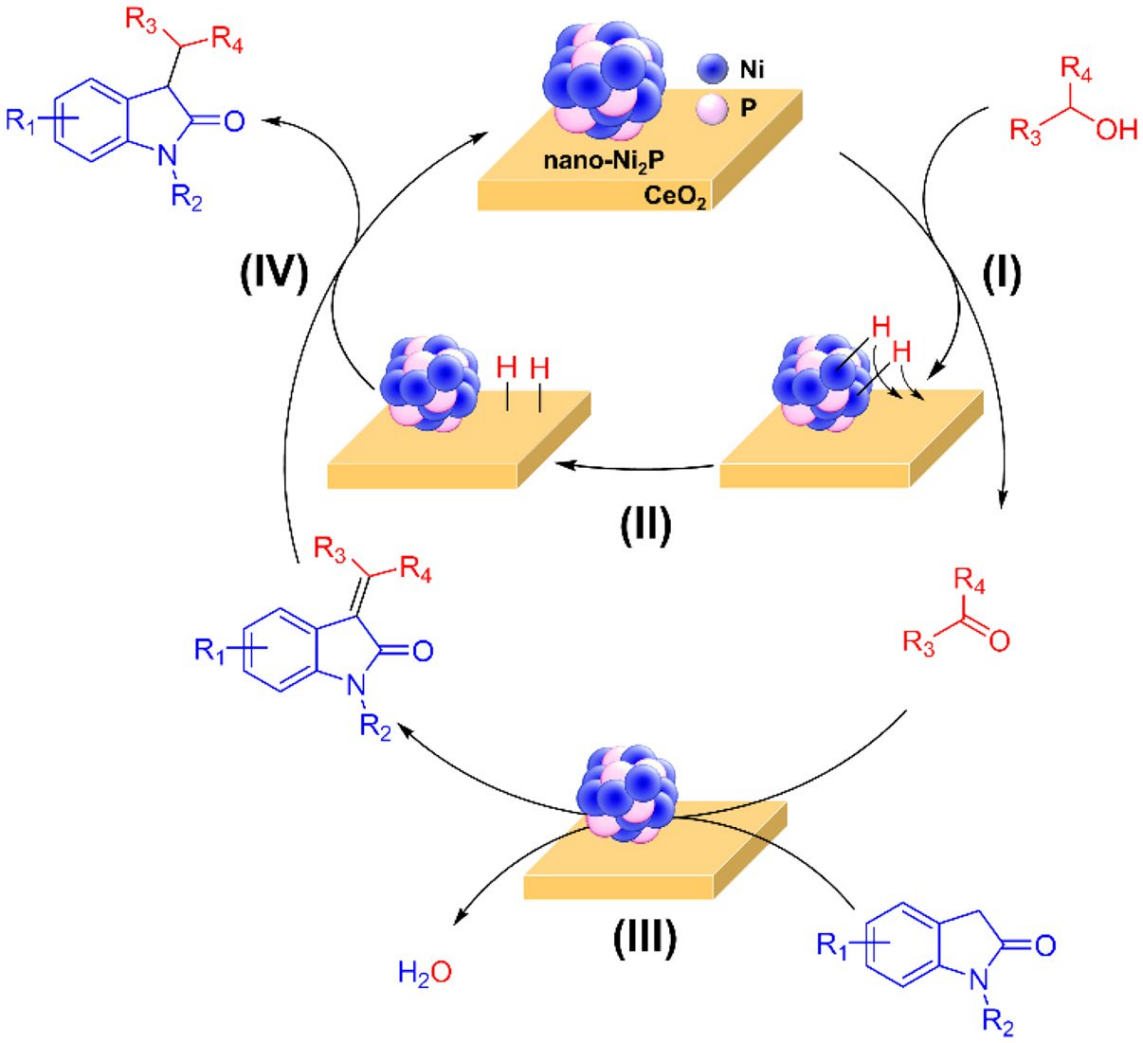
Proposed reaction pathway for the C-3 alkylation of oxindoles with alcohols catalyzed by nano-Ni_2_P/CeO_2_.

Figure 3 shows the time profile of the C-3 alkylation of oxindole with benzyl alcohol catalyzed by nano-Ni_2_P/CeO_2_. During the first 4 h, oxindole was rapidly consumed, accompanied by the formation of **2a**. Afterwards, the yield of **2a** gradually decreased with an increase in the yield of **1a**. These results indicate that **2a** is an intermediate in the C-3 alkylation of oxindole with benzyl alcohol. To clarify the roles of nano-Ni_2_P and CeO_2_ in the C-3 alkylation of oxindole using alcohols, control experiments were conducted, as outlined in Fig. 4. Initially, a reaction using oxindole and benzyl alcohol was carried out in the presence of nano-Ni_2_P, CeO_2_, or nano-Ni_2_P/CeO_2_ under the same reaction conditions as presented in Table 1, with the exception that a shorter reaction time was employed (i.e., 3 h) (Fig. 4a). As shown, nano-Ni_2_P/CeO_2_ promoted the reaction, giving **1a** and **2a** in 20 and 60% yields, respectively. On the other hand, nano-Ni_2_P yielded a trace of the desired product **1a**, and reaction intermediate **2a** was formed in 14% yield. When CeO_2_ was used, the reaction produced only small amounts of **1a** and **2a**. Subsequently, the condensation of oxindole with benzaldehyde was carried out using nano-Ni_2_P or CeO_2_ (Fig. 4b). When either nano-Ni_2_P or CeO_2_ was used, the aldol-type condensation proceeded efficiently to provide **2a** in a high yield. In a blank test, no formation of **2a** was observed, indicating that nano-Ni_2_P and CeO_2_ are active for the condensation reaction. The above results demonstrate that benzaldehyde is produced on nano-Ni_2_P by the dehydrogenation of benzyl alcohol, and subsequently, the aldol-type condensation of benzaldehyde with oxindole occurs on either nano-Ni_2_P or CeO_2_^72^. It is therefore considered that the hydrogen generated on nano-Ni_2_P during dehydrogenation may spill over onto the CeO_2_ surface, thereby allowing the hydrogenation of **2a** to **1a** on CeO_2_^73,74^. Indeed, hydrogen spillover on metal nanoparticle-supported CeO_2_ and hydrogen transfer catalysis by CeO_2_ have both been reported^75,76^. Considering the above information, we proposed a reaction pathway for the C-3 alkylation of oxindole with alcohol catalyzed by nano-Ni_2_P/CeO_2_ using the BH methodology (Scheme 4). Initially, the alcohol is dehydrogenated to the corresponding carbonyl compound by nano-Ni_2_P (I). The hydrogen generated on the nano-Ni_2_P then spills over onto the CeO_2_ surface (II), and the aldol-type condensation of oxindole with the carbonyl compound occurs catalyzed by either nano-Ni_2_P or CeO_2_ to provide an alkenyloxindole intermediate (III). Finally, the subsequent hydrogenation of alkenyloxindole following hydrogen spillover to CeO_2_ yields the desired C3-alkylated product (IV). This well-designed cooperative catalysis by nano-Ni_2_P and CeO_2_ is a key factor in efficiently promoting the C-3 alkylation of oxindole using alcohols.

## Conclusions

We herein report the development of a highly efficient and reusable non-precious metal-based heterogeneous catalyst for promoting the C-3 alkylation of oxindoles with alcohols. More specifically, a cerium dioxide-supported nickel phosphide nanoalloy (nano-Ni_2_P/CeO_2_) catalyst efficiently promoted the C-3 alkylation of oxindoles with alcohols. This catalytic system was applicable to various alcohols, including benzylic and aliphatic alcohols, providing the corresponding products in high yields. Indeed, this is the first catalytic system for the C-3 alkylation of oxindoles with alcohols using non-precious metal-based heterogeneous catalysts. Furthermore, nano-Ni_2_P/CeO_2_ was easily recoverable and reusable without any significant loss in activity. The catalytic activity of nano-Ni_2_P/CeO_2_ was high, and was comparable to those of previously reported precious metal-based catalysts. In this reaction, the cooperation between nano-Ni_2_P and CeO_2_ was found to play a key role; nano-Ni_2_P dehydrogenates the alcohol to generate the corresponding carbonyl compound and hydrogen. Subsequently, nano-Ni_2_P or CeO_2_ promotes the aldol condensation of oxindoles with the produced carbonyl compound to provide an alkenyl oxindole. CeO_2_ then receives hydrogen from nano-Ni_2_P and hydrogenates the C-3 alkenyl oxindole to give the desired product. Such concerted catalysis by nano-Ni_2_P and CeO_2_ occurs efficiently, leading to a high catalytic performance in the C-3 alkylation of oxindoles with alcohols. The results of this study also demonstrate that metal phosphides have great potential as highly efficient heterogeneous catalysts, not only in hydrogenation reactions, but also in various other organic syntheses, through concerted effects with metal oxide supports.

## Supplementary Information

Supplementary Information.
