# Supplementary material for: A nickel phosphide nanoalloy catalyst for the C-3 alkylation of oxindoles with alcohols
Source: Sci Rep. 2021 May 21;11:10673. doi: 10.1038/s41598-021-89561-1 (PMC8140154; doi:10.1038/s41598-021-89561-1)
Supplement: Supplementary file 1 — Supplementary Information. [file 41598_2021_89561_MOESM1_ESM.docx]

**Supplementary Information**

**A Nickel Phosphide Nanoalloy Catalyst for the C-3 Alkylation**

**of Oxindoles with Alcohols**

Shu Fujita,^a^ Kohei Imagawa,^a^ Sho Yamaguchi,^a^ Jun Yamasaki,^b^ Seiji Yamazoe,^c^

Tomoo Mizugaki^a,d^ and Takato Mitsudome*^a^

^a^Department of Materials Engineering Science, Graduate School of Engineering Science, Osaka University, 1-3 Machikaneyama, Toyonaka, Osaka 560-8531, Japan.

E-mail: mitsudom@cheng.es.osaka-u.ac.jp

^b^Department of Materials Engineering Science, Graduate School of Engineering Science, Osaka University, 1-3 Machikaneyama, Toyonaka, Osaka 560-8531, Japan.

^c^Department of Chemistry, Tokyo Metropolitan University, 1-1 Minami Osawa, Hachioji, Tokyo 192-0397, Japan.

^d^Innovative Catalysis Science Division, Institute for Open and Transdisciplinary Research Initiatives (ICS-OTRI), Osaka University, Suita, Osaka 565-0871, Japan.

**Table of Contents**

1. **General experimental details**
2. **Catalyst preparation**

**Figure S1.** The photo of nano-Ni_2_P/CeO_2_.

1. **Typical reaction procedure**
2. **Recycling experiments**
3. **Characterization and catalytic activities of the nano-Ni_2_P catalysts**

**Figure S2.** TEM images of nano-Ni_2_P supported on (a) TiO_2_, (b) SiO_2_, (c) Al_2_O_3_, (d) HT, (e) MgO, (f) Nb_2_O_5_, and (g) ZnO.

**Figure S3.** XRD pattern of nano-Ni_2_P.

**Figure S4.** Ni *K*-edge XANES spectra of Ni foil, NiO, nano-Ni_2_P, and nano-Ni_2_P/CeO_2_.

**Figure S5.** XPS spectrum of Ni 2p for nano-Ni_2_P/CeO_2_.

**Table S1.** BET surface area of the supported nano-Ni_2_P catalysts.

**Table S2.** C-3 alkylation of oxindole with benzyl alcohol using heterogeneous catalysts.

**Table S3.** ICP-AES elemental analysis of fresh and used nano-Ni_2_P/CeO_2_.

1. **Characterization data for products**
2. **^1^H NMR and ^13^C NMR spectra of products**

**References**

**1. General experimental details**

NiCl_2_·6H_2_O (98%) and Nb_2_O_5_ were purchased from Wako Pure Chemical Corporation (Japan). Triphenylphosphite (97%) and hexadecylamine (95%) were purchased from Tokyo Chemical Industry Co., Ltd. (Tokyo, Japan). CeO_2_ (JRC-CEO-2), TiO_2_ (JRC-TIO-15), and MgO (JRC-MGO-3) as reference catalysts, were obtained from the Catalysis Society of Japan. SiO_2_ (Q-6) was purchased from Fuji Silysia Chemical (Japan). Al_2_O_3_ (AKP-G015) was obtained from Sumitomo Chemical Co., Ltd. (Japan). Hydrotalcite (HT, AD-500NS, Mg_6_Al_2_(OH)_16_CO_3_·4H_2_O) was obtained from Tomita Pharmaceutical Co. Ltd. (Japan). ZnO was obtained from Sigma-Aldrich Japan. BET measurement was carried out using BELCAT-A (BEL Japan Inc). X-ray diffraction (XRD) measurements were performed using a Philips X’Pert-MPD diffractometer with Cu-Kα radiation. Transmission electron microscopy (TEM) was conducted on a FEI Tecnai G2 20ST instrument operating at 200 kV. Scanning transmission electron microscopy (STEM) images and elemental maps were collected using a 200 kV instrument (JEM-ARM200F, JEOL) equipped with a cold field-emission gun. Gas chromatography–mass spectrometry (GC-MS) was performed using a GCMS-QP2010 SE (Shimadzu, Japan) instrument equipped with an inert Cap WAX-HT capillary column (30 m × 0.25 mm i.d., film thickness 0.25 µm). The oven temperature was programmed as follows: starting temperature, 60 °C; hold at 60 °C for 2 min; ramp to 240 °C at a rate of 20 °C/min. Inductively coupled plasma-atomic emission spectrometry (ICP-AES) was performed using an ICPS-8100 instrument (Shimadzu, Japan). ^1^H and ^13^C nuclear magnetic resonance (NMR) spectra were recorded using a JEOL JNM-ESC400 spectrometer.

**2. Catalyst preparation**

**Synthesis of nano-Ni_2_P**

In a typical synthetic procedure, NiCl_2_·6H_2_O (1.0 mmol) was combined with hexadecylamine (10 mmol) and triphenyl phosphite (10 mmol) in a Schlenk flask. The temperature was then increased to 320 °C under an argon atmosphere and held for 2 h with stirring, to afford a black colloidal solution. The mixture was then allowed to cool to 25 °C, and the black product was isolated by precipitation in acetone. The redispersion and precipitation cycle was repeated using a chloroform–acetone solvent mixture. The obtained powder was dried *in vacuo* overnight.

**Synthesis of the nano-Ni_2_P/Support catalyst**

Typically, nano-Ni_2_P (30 mg) was dispersed in hexane (100 mL) via sonication for 1 h and then stirred with CeO_2_ (1.0 g) for 6 h at room temperature. The obtained product was dried *in vacuo* overnight to yield a gray nano-Ni_2_P/CeO_2_ powder. A similar procedure was followed to prepare the other nano-Ni_2_P/support (TiO_2_, SiO_2_, Al_2_O_3_, HT, MgO, Nb_2_O_5_, and ZnO) catalysts. The photo of the prepared Ni_2_P/CeO_2_ catalyst is shown in Figure S1.


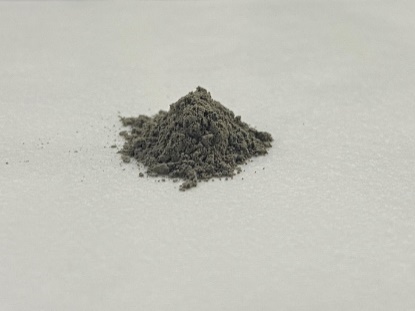


**Figure S1.** The photo of nano-Ni_2_P/CeO_2_.

**Synthesis of Ni/CeO_2_ and Ni/CeO_2_-Red**

Ni(NO_3_)_2_·6H_2_O (0.2 mmol) was added to acetone (50 mL) with stirring. After 10 min, CeO_2_ (1.0 g) was added and the mixture was stirred for a further 1 h. Acetone was then removed by evaporation under reduced pressure. The obtained powder was dried *in vacuo* overnight and calcined in air at 500 °C for 3 h to give Ni/CeO_2_. Ni/CeO_2_ was then treated with H_2_ under atmospheric pressure at 600 °C for 2 h to afford Ni/CeO_2_-Red.

**3. Typical reaction procedure**

A typical reaction procedure for the C-3 alkylation of oxindole with benzyl alcohol using nano-Ni_2_P/CeO_2_ is described below. More specifically, nano-Ni_2_P/CeO_2_ (0.15 g) was placed in a 30 mL stainless-steel autoclave, followed by the addition of oxindole (0.5 mmol), benzyl alcohol (1 mmol), and toluene (2 mL). The reaction mixture was then stirred vigorously at 140 °C under N_2_. The reaction solution was then analyzed by GC–MS to determine the conversion and yield using an internal standard method.

The turnover numbers are calculated as follows:

TON = $\frac{the mol of C-3 alkylated oxindole product}{the mol of Ni used in the reaction}$

**4. Recycling experiments**

After the catalytic reaction, the catalyst was separated from the reaction mixture by centrifugation, washed with ethanol and toluene, and then reused in the subsequent reaction.

**5. Characterization and catalytic activities of the nano-Ni_2_P catalysts**


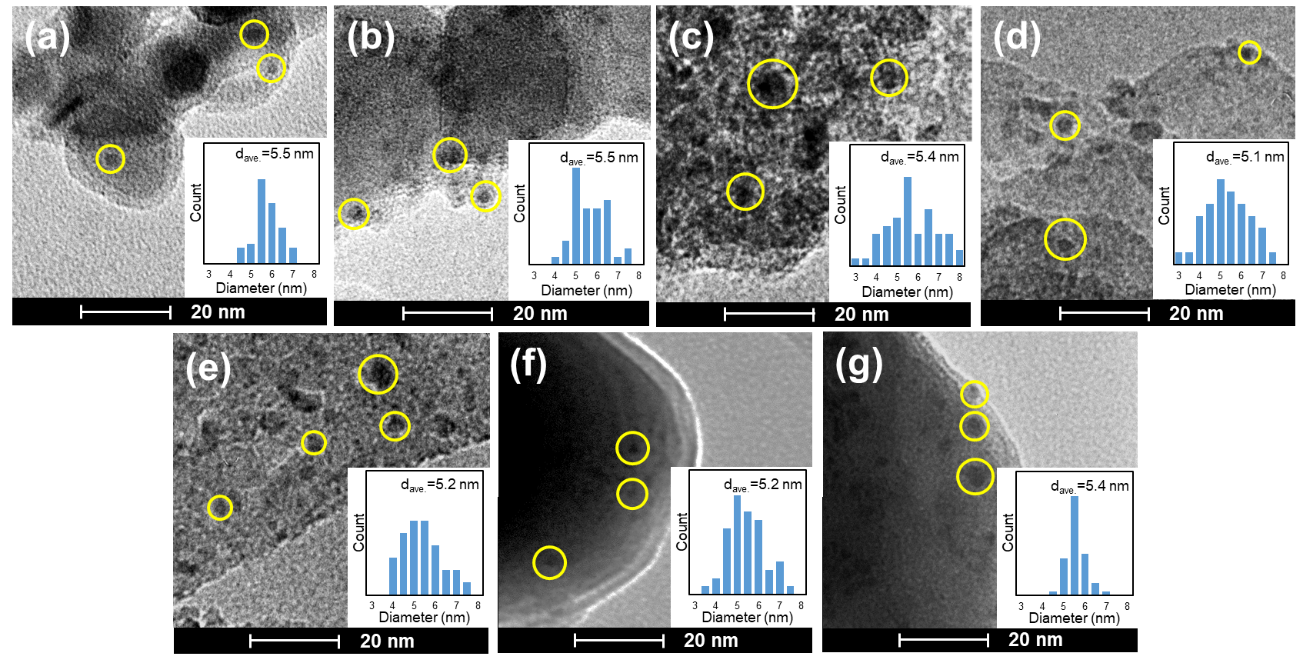


**Figure S2.** TEM images of nano-Ni_2_P supported on (a) TiO_2_, (b) SiO_2_, (c) Al_2_O_3_, (d) HT, (e) MgO, (f) Nb_2_O_5_, and (g) ZnO.


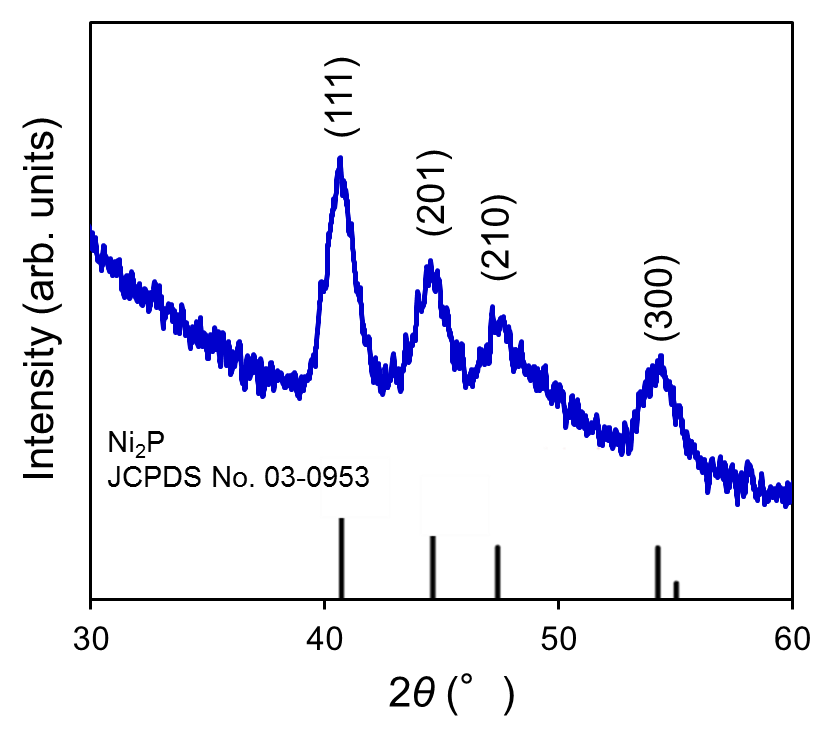


**Figure S3.** XRD pattern of nano-Ni_2_P.

**
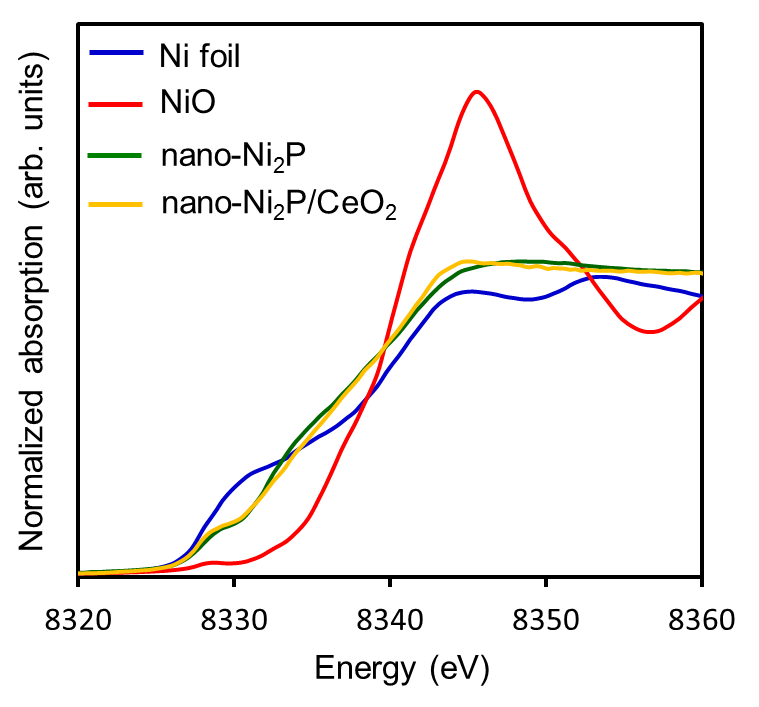
**

**Figure S4.** Ni *K*-edge XANES spectra of Ni foil, NiO, nano-Ni_2_P, and nano-Ni_2_P/CeO_2_.

**
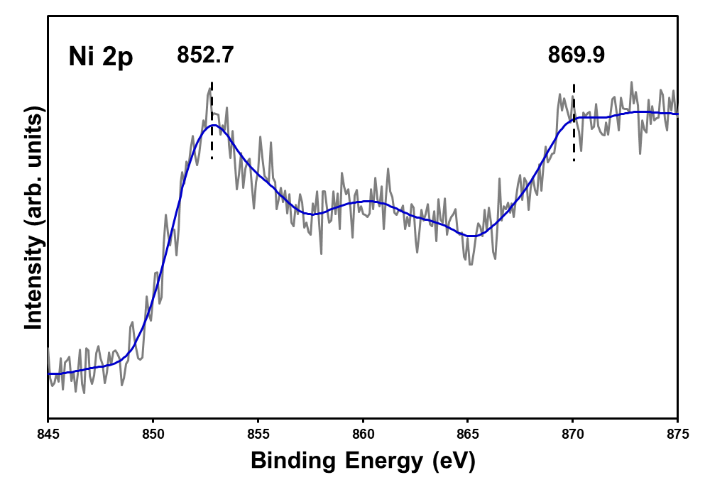
**

**Figure S5.** XPS spectrum of Ni 2p for nano-Ni_2_P/CeO_2_.

**Table S1.** BET surface area of the supported nano-Ni_2_P catalysts.

| Samples | BET areas (m^2^·g^-1^) |
| --- | --- |
| nano-Ni_2_P/CeO_2_ | 121 |
| nano-Ni_2_P/TiO_2_ | 63.3 |
| nano-Ni_2_P/SiO_2_ | 270 |
| nano-Ni_2_P/Al_2_O_3_ | 160 |
| nano-Ni_2_P/HT | 53.1 |
| nano-Ni_2_P/MgO | 21.1 |
| nano-Ni_2_P/Nb_2_O_5_ | 4.10 |
| nano-Ni_2_P/ZnO | 4.50 |

**Table S2.** C-3 alkylation of oxindole with benzyl alcohol using heterogeneous catalysts.

| Catalyst | Conditions | Yield [%] | TON | Ref. |
| --- | --- | --- | --- | --- |
| nano-Ni_2_P/CeO_2_ | 140 °C, 10 h in toluene | 95 | 19 | This work |
| nano-Ni_2_P/CeO_2_ | 140 °C, 72 h in toluene | 85 | 212 | This work |
| Me-Ir-In-SBA-15 | KOH (0.4 mmol), 110 °C, 24 h in toluene | 93.5 | 144 | 1 |
| Pt/CeO_2_ | 165 °C, 24 h in mesitylene | 99 | 99 | 2 |
| Pd/C | KOH (0.2 mmol), 120 °C, 24 h in dioxane | 99 | 10 | 3 |
| Raney Ni | 220 °C, 4 h in methanol | 89 | 0.52 | 4 |

**Table S3.** ICP-AES elemental analysis of fresh and used nano-Ni_2_P/CeO_2_.

|  | Ni (wt%) |
| --- | --- |
| Fresh nano-Ni_2_P/CeO_2_ | 1.03 |
| Used nano-Ni_2_P/CeO_2_ | 1.03 |

**6. Characterization data for products**

All reaction products were characterized by GC-MS and NMR. The retention times (GC-MS) and chemical shifts (^1^H and ^13^C) of the products were in agreement with those of previously reported values.

**3-Benzyl-2-oxindole** (**1a**)^5^

CAS registry No. [7511-08-2]. ^1^H NMR (400 MHz, CDCl_3_, ppm): δ = 9.28 (brs, 1H), 7.25–7.13 (m, 6H), 6.90–6.86 (m, 2H), 6.72 (d, *J* = 7.2 Hz, 1H), 3.74 (dd, *J* = 9.2, 4.4 Hz, 1H), 3.49 (dd, *J* = 13.6, 4.4 Hz, 1H), 2.92 (dd, *J* = 13.6, 9.2 Hz, 1H); ^13^C NMR (100 MHz, CDCl_3_, ppm) δ = 180.0, 141.5, 137.8, 129.4, 129.0, 128.3, 127.9, 126.6, 124.7, 121.9, 109.8, 47.6, 36.6.

**1,3-Dihydro-3-[(4-methylphenyl)methyl]-2*H*-indol-2-one** (**1b**)^3^

CAS registry No. [170956-94-2]. ^1^H NMR (400 MHz, CDCl_3_, ppm): δ = 8.71 (brs, 1H), 7.17–7.13 (m, 1H), 7.07–7.05 (m, 4H), 6.91–6.73 (m, 4H), 3.71 (dd, *J* = 9.2, 7.6 Hz, 1H), 3.43 (dd, *J* = 13.2, 4.4, 1H), 2.88 (dd, *J* = 14.0, 9.6 Hz, 1H), 2.30 (s, 3H); ^13^C NMR (100 MHz, CDCl_3_, ppm) δ = 179.7, 141.4, 136.1, 134.7, 129.2, 129.1, 129.0, 127.9, 124.8, 122.0, 109.7, 47.6, 36.2, 21.0.

**3-[(4-Fluorophenyl)methyl]-1,3-dihydro-2*H*-indol-2-one** (**1c**)^3^

CAS registry No. [931927-82-1]. ^1^H NMR (400 MHz, CDCl_3_, ppm): δ = 9.06 (brs, 1H), 7.17–7.12 (m, 2H), 7.03–6.97 (m, 3H), 6.90–6.85 (m, 2H), 6.72 (d, *J* = 7.2 Hz, 1H), 3.73 (dd, *J* = 8.8, 4.0 Hz, 1H), 3.47 (dd, *J* = 14.0, 4.8 Hz, 1H), 2.85 (dd, *J* = 13.2, 9.6 Hz, 1H), 2.28 (s, 3H); ^13^C NMR (100 MHz, CDCl_3_, ppm) δ = 179.9, 141.5, 137.9, 137.8, 130.1, 129.1, 128.2, 127.9, 127.4, 126.4, 124.8, 121.9, 109.7, 47.5, 36.6, 21.3.

**3-[(4-Chlorophenyl)methyl]-1,3-dihydro-2*H*-indol-2-one** (**1d**)^6^

CAS registry No. [131609-35-3]. ^1^H NMR (400 MHz, CDCl_3_, ppm): δ = 9.10 (brs, 1H), 7.19–7.15 (m, 3H), 7.07 (d, *J* = 8.0 Hz, 2H), 6.93 (t, *J* = 7.6 Hz, 1H), 6.84–6.83 (m, 2H), 3.72 (dd, *J* = 8.0, 4.4 Hz, 1H), 3.40 (dd, *J* = 14.4, 4.8 Hz, 1H), 2.98 (dd, *J* = 13.6, 8.8 Hz, 1H); ^13^C NMR (100 MHz, CDCl_3_, ppm) δ = 179.6, 141.5, 136.0, 132.5, 130.7, 128.5, 128.4, 128.1, 124.6, 122.1, 109.9, 47.3, 35.8.

**1,3-​Dihydro-​3-​[[4-​(trifluoromethyl)​phenyl]​methyl]​-2*H*-​indol-​2-​one** (**1e**)^5^

CAS registry No. [31609-35-3]. ^1^H NMR (400 MHz, CDCl_3_, ppm): δ = 9.10 (brs, 1H), 7.16 (m, 3H), 7.07 (d, *J* = 8.0 Hz, 2H), 6.93 (t, *J* = 7.6 Hz, 1H), 6.84–6.83 (m, 2H), 3.72 (dd, *J* = 8.0, 4.4 Hz, 1H), 3.40 (dd, *J* = 14.4, 4.8 Hz, 1H), 2.98 (dd, *J* = 13.6, 8.8 Hz, 1H); ^13^C NMR (100 MHz, CDCl_3_, ppm) δ = 179.6, 141.5, 136.0, 132.5, 130.7, 128.5, 128.4, 128.1, 124.6, 122.1, 109.9, 47.3, 35.8.

**3-[(4-Methoxyphenyl)methyl]-1,3-dihydro-2*H*-indol-2-one** (**1f**)^5^

CAS registry No. [170956-93-1]. ^1^H NMR (400 MHz, CDCl_3_, ppm): δ = 9.16 (brs, 1H), 7.49–7.42 (m, 2H), 7.27 (d, *J* = 8.0 Hz, 2H), 7.18 (t, *J* = 7.8 Hz, 1H), 6.94 (t, *J* = 7.6 Hz, 1H), 3.77 (dd, *J* = 8.0, 4.4 Hz, 1H), 3.48 (dd, *J* = 13.6, 4.4 Hz, 1H), 3.08 (dd, *J* = 13.6, 8.8 Hz, 1H); ^13^C NMR (100 MHz, CDCl_3_, ppm) δ = 179.5, 141.7, 141.4, 129.7, 129.0 (q, *J* = 32.5 Hz), 128.4, 128.2, 125.4 (q, *J* = 4.5 Hz), 125.2 (q, *J* = 3.8 Hz), 124.5, 122.2, 110.0, 47.2, 36.2.

**4-​[(2,​3-Ddihydro-​2-​oxo-​1*H*-​indol-​3-​yl)​methyl]​-benzonitrile** (**1g**)^5^

CAS registry No. [170956-95-3]. ^1^H NMR (400 MHz, CDCl_3_, ppm): δ = 7.17–7.13 (m, 1H), 7.08–7.06 (m, 2H), 6.92–6.83 (m, 2H), 6.80–6.75 (m, 3H), 3.75 (s, 3H), 3.72–3.68 (m, 1H), 3.42 (dd, *J* = 14.4, 4.8 Hz, 1H), 2.90 (dd, *J* = 13.6, 9.2 Hz, 1H); ^13^C NMR (100 MHz, CDCl_3_, ppm) δ = 179.9, 158.3, 141.5, 130.3, 129.7, 12.9.0, 127.9, 124.7, 121.9, 113.6, 109.8, 55.1, 47.7, 35.7.

**3-​([1,​1'-​Biphenyl]​-​4-​ylmethyl)​-​1,​3-​dihydro-2*H*-​indol-​2-​one** (**1h**)^7^

CAS registry No. [1268496-02-1]. ^1^H NMR (400 MHz, CDCl_3_, ppm): δ = 9.13 (brs, 1H), 7.55–7.53 (m, 2H), 7.47–7.45 (m, 2H), 7.42–7.38 (m, 2H), 7.33–7.29 (m, 1H), 7.22 (d, *J* = 8.4 Hz, 2H), 7.18–7.14 (m, 1H), 6.92–6.80 (m, 3H), 3.77 (dd, *J* = 9.2, 4.8 Hz, 1H), 3.51 (dd, *J* = 13.6, 4.4 Hz, 1H), 2.97 (dd, *J* = 13.6, 9.6 Hz, 1H); ^13^C NMR (100 MHz, CDCl_3_, ppm) δ = 179.7, 141.5, 140.7, 139.4, 136.8, 129.8, 129.0, 128.7, 128.0, 127.1, 126.9, 126.9, 124.8, 122.0, 109.8, 47.5, 36.2.

**1,​3-​Dihydro-​3-​(2-​naphthalenylmethyl)​-2*H*-​indol-​2-​one** (**1i**)^3^

CAS registry No. [1202521-48-9]. ^1^H NMR (400 MHz, CDCl_3_, ppm): δ = 7.80–7.70 (m, 3H), 7.61 (s, 1H), 7.44–7.42 (m, 2H), 7.33 (dd, *J* = 8.0, 1.2 Hz, 1H), 7.13 (t, *J* = 7.8 Hz, 1H), 6.87–6.81 (m, 2H), 6.73 (d, *J* = 7.2 Hz, 1H), 3.83 (dd, *J* = 9.2, 3.6 Hz, 1H), 3.63 (dd, *J* = 9.2, 4.8 Hz, 1H), 3.08 (dd, *J* = 14.0, 9.2 Hz, 1H); ^13^C NMR (100 MHz, CDCl_3_, ppm) δ = 179.7, 141.4, 135.3, 133.3, 132.3, 128.9, 128.0, 128.0, 127.6, 127.6, 127.5, 126.0, 125.5, 124.8, 122.0, 109.8, 47.4, 36.8.

**3-​(1,​3-Bbenzodioxol-​5-​ylmethyl)​-​1,​3-​dihydro-2*H*-​indol-​2-​one** (**1j**)^6^

CAS registry No. [330574-07-7]. ^1^H NMR (400 MHz, CDCl_3_, ppm): δ = 8.91 (brs, 1H), 7.17 (t, *J* = 7.6 Hz, 1H), 6.92 (t, *J* = 7.6 Hz, 1H), 6.84 (dd, *J* = 10.8, 8.0 Hz, 2H), 6.67–6.60 (m, 3H), 5.90 (s, 2H), 3.68 (dd, *J* = 8.8, 4.8 Hz, 1H), 3.39 (dd, *J* = 13.6, 4.4 Hz, 1H), 2.88 (dd, *J* = 14.0, 8.8 Hz, 1H); ^13^C NMR (100 MHz, CDCl_3_, ppm) δ = 179.7, 147.5, 146.2, 141.4, 131.4, 128.9, 127.9, 124.7, 122.5, 122.0, 109.8, 109.6, 108.0, 100.8, 47.7, 36.3.

**3-​(2-​Furanylmethyl)​-​1,​3-​dihydro-2*H*-​indol-​2-​one** (**1k**)^5^

CAS registry No. [175092-95-2]. ^1^H NMR (400 MHz, CDCl_3_, ppm): δ = 8.10 (brs, 1H), 7.22–7.18 (m, 1H), 7.09 (d, *J* = 4.8 Hz, 1H), 6.97–6.95 (m, 2H), 6.87–6.78 (m, 3H), 3.75 (dd, *J* = 8.4, 4.8 Hz, 1H), 3.60 (dd, *J* = 14.4, 4.0 Hz, 1H), 3.34 (dd, *J* = 14.8, 8.0 Hz, 1H); ^13^C NMR (100 MHz, CDCl_3_, ppm) δ = 178.5, 141.5, 139.6, 128.5, 128.2, 126.7, 126.4, 124.7, 124.3, 122.3, 109.6, 47.5, 30.7.

**1,​3-Dihydro-​3-​(2-​thienylmethyl)​-2*H*-​indol-​2-​one** (**1l**)^8^

CAS registry No. [175092-97-4]. ^1^H NMR (400 MHz, CDCl_3_, ppm): δ = 8.89 (brs, 1H), 8.44–8.40 (m, 2H), 7.18 (t, *J* = 7.6 Hz, 1H), 7.12–7.08 (m, 2H), 6.97–6.89 (m, 2H), 6.81 (d, *J* = 7.6 Hz, 1H), 3.79 (dd, *J* = 8.0, 4.8 Hz, 1H), 3.40 (dd, *J* = 14.0, 4.8 Hz, 1H), 3.08 (dd, *J* = 13.6, 7.6, 1H); ^13^C NMR (100 MHz, CDCl_3_, ppm) δ = 178.8, 149.4, 141.5, 128.3, 128.0, 124.4, 122.2, 109.9, 46.4, 35.9.

**1,​3-​Dihydro-​3-​(4-​pyridinylmethyl)​-2*H*-​indol-​2-​one** (**1m**)^5^

CAS registry No. [3367-86-0]. ^1^H NMR (400 MHz, CDCl_3_, ppm): δ = 8.43 (brs, 1H), 7.33 (s, 1H), 7.20–7.14 (m, 1H), 6.95–6.70 (m, 1H), 6.86 (d, *J* = 7.6 Hz, 1H), 6.79 (d, *J* = 8.0, 1H), 6.29 (t, *J* = 2.2 Hz, 1H), 6.03 (d, *J* = 2.8 Hz, 1H), 3.80 (dd, *J* = 9.2, 4.4 Hz, 1H), 3.47 (dd, *J* = 14.4, 4.0 Hz, 1H), 2.98 (dd, *J* = 15.2, 9.6 Hz, 1H); ^13^C NMR (100 MHz, CDCl_3_, ppm) δ = 179.0, 151.9, 141.5, 141.3, 128.7, 128.1, 124.7, 122.3, 110.3, 109.6, 107.2, 45.1, 29.0.

**3-​Ethyl-​1,​3-​dihydro-2*H*-​indol-​2-​one** (**1n**)^9^

CAS registry No. [15379-45-0]. ^1^H NMR (400 MHz, CDCl_3_, ppm): δ = 8.27 (brs, 1H), 7.24–7.19 (m, 2H), 7.03 (t, *J* = 7.4 Hz, 1H), 6.89 (d, *J* = 8.0 Hz, 1H), 3.46 (t, *J* = 5.8 Hz, 1H), 2.09–2.00 (m, 2H), 0.93 (t, *J* = 7.6 Hz, 3H); ^13^C NMR (100 MHz, CDCl_3_, ppm) δ = 180.1, 141.6, 129.5, 127.8, 124.1, 122.2, 109.5, 47.0, 23.6, 10.0.

**1,​3-​Dihydro-​3-​octyl-2*H*-​indol-​2-​one** (**1o**)

CAS registry No. [1266682-04-5]. ^1^H NMR (400 MHz, CDCl_3_, ppm): δ = 7.85 (brs, 1H), 7.24–7.14 (m, 2H), 7.04–6.95 (m, 1H), 6.88–6.83 (m, 1H), 3.46 (t, *J* = 6.0 Hz, 1H), 2.01–1.94 (m, 2H), 1.58–1.14 (m, 12H), 0.85 (m, 3H); ^13^C NMR (100 MHz, CDCl_3_, ppm) δ = 180.0, 141.5, 129.9, 127.7, 124.3, 122.1, 109.4, 45.9, 31.8, 30.6, 29.6, 29.3, 29.2, 25.8, 22.6, 14.1.

**1,​3-​Dihydro-​3-​(2-​methylpropyl)​-2*H*-​indol-​2-​one** (**1p**)^7^

CAS registry No. [251550-17-1]. ^1^H NMR (400 MHz, CDCl_3_, ppm): δ = 8.82 (brs, 1H), 7.26–7.19 (m, 2H), 7.02 (t, *J* = 7.2 Hz, 1H), 6.91 (d, *J* = 8.0 Hz, 1H), 3.47 (t, *J* = 6.0 Hz, 1H), 2.02–1.86 (m, 2H), 1.61–1.30 (m, 3H), 1.23–1.17 (m, 2H), 0.84 (d, *J* = 6.4 Hz, 6H); ^13^C NMR (100 MHz, CDCl_3_, ppm) δ = 180.7, 141.6, 129.9, 127.7, 124.1, 122.2, 109.7, 46.1, 38.8, 30.7, 27.7, 23.6, 22.5.

**3-​(Cyclohexylmethyl)​-​1,​3-​dihydro-2*H*-​indol-​2-​one** (**1q**)^1^

CAS registry No. [81609-20-3]. ^1^H NMR (400 MHz, CDCl_3_, ppm): δ = 9.47 (brs, 1H), 7.19 (t, *J* = 6.0 Hz, 1H), 7.00 (t, *J* = 7.6 Hz, 1H), 6.93 (d, *J* = 7.6 Hz, 1H), 3.55–3.44 (m, 1H), 1.94–1.81 (m, 2H), 1.75–1.64 (m, 6H), 1.32–1.12 (m, 3H), 1.03–0.95 (m. 2H); ^13^C NMR (100 MHz, CDCl_3_, ppm) δ = 181.7, 141.6, 130.3, 127.6, 124.3, 122.0, 43.6, 38.4, 34.6, 33.6, 32.7, 26.4, 26.1, 26.1.

**1,​3-​Dihydro-​3-​(4-​hydroxybutyl)​-2*H*-​indol-​2-​one** (**1r**)^4^

CAS registry No. [204012-72-6]. ^1^H NMR (400 MHz, CDCl_3_, ppm): δ = 9.18 (brs, 1H), 7.22–7.17 (m, 2H), 6.99 (d, *J* = 8.0 Hz, 1H), 3.59 (t, *J* = 6.4 Hz, 2H), 3.47 (t, *J* = 5.8 Hz, 1H), 2.38 (brs, 1H), 2.01–1.96 (m, 2H), 1.68–1.33 (m, 4H); ^13^C NMR (100 MHz, CDCl_3_, ppm) δ = 180.8, 141.7, 129.6, 127.8, 124.0, 122.2, 109.8, 62.2, 46.0, 32.5, 30.1, 21.9.

**1,​3-​Dihydro-​3-​(1-​phenylethyl)​-2*H*-​indol-​2-​one** (**1s**)^3^

CAS registry No. [140701-16-2]. ^1^H NMR (400 MHz, CDCl_3_, ppm): major diastereomer: δ = 8.63 (brs, 1H), 7.36–6.83 (m, 8H), 6.51 (d, *J* = 7.2 Hz, 1H), 3.83–3.77 (m, 2H),1.19 (d, *J* = 7.2 Hz, 3H); minor diastereomer: δ = 8.27 (brs, 1H), 7.36–6.83 (m, 8H), 6.74 (d, *J* = 8.0, 1H), 3.65 (d, *J* = 5.2 Hz, 1H). 3.54–3.47 (m, 1H), 1.63 (d, *J* = 7.6 Hz, 3H); ^13^C NMR (100 MHz, CDCl_3_, ppm) δ = 179.3, 178.9, 142.7, 142.1, 141.9, 141.6, 128.3, 128.1, 128.0, 127.9, 127.8, 126.9, 126.7, 126.6, 125.1, 121.9, 121.8, 109.5, 52.9, 52.4, 41.8, 39.5, 19.2, 13.5.

**1,​3-​Dihydro-​3-​[(1R)​-​1-​(4-​methylphenyl)​ethyl]​- 2*H*-​indol-​2-​one** (**1t**)^12^

CAS registry No. [2460575-92-0, 2460575-94-2]. ^1^H NMR (400 MHz, CDCl_3_, ppm): major diastereomer: δ = 7.91 (s, 1H), 7.13–7.00 (m, 4H), 6.90–6.63 (m, 3H), 6.49 (d, *J* = 7.2 Hz, 1H), 3.68 (d, *J* = 4.8 Hz, 1H), 3.56 (d, *J* = 5.6 Hz, 1H), 2.28 (s, 3H), 1.53 (d, *J* = 7.6 Hz, 3H); minor diastereomer: δ = 7.59 (s, 1H), 7.13–7.00 (m, 4H), 6.90–6.63 (m, 3H), 6.64 (d, *J* = 8.0 Hz, 1H), 3.47–3.44 (m, 1H), 3.42–3.37 (m, 1H), 2.18 (s, 3H), 1.10 (d, *J* = 7.6 Hz, 3H); ^13^C NMR (100 MHz, CDCl_3_, ppm) δ = 178.9, 178.4, 141.7, 141.5, 139.7, 139.1, 136.2, 129.0, 128.7, 128.2, 127.9, 127.9, 127.8, 127.7, 127.1, 125.2, 121.9, 109.3, 109.3, 52.9, 52.3, 41.5, 39.2, 21.0, 19.4, 13.7.

**1,​3-​Dihydro-​1-​methyl-​3-​(phenylmethyl)​-2*H*-​indol-​2-​one** (**1u**)^10^

CAS registry No. [3335-85-1]. ^1^H NMR (400 MHz, CDCl_3_, ppm): δ = 7.37–7.15 (m, 5H), 6.91 (t, *J* = 7.2 Hz, 1H), 6.76-6.72 (m, 2H), 3.71 (dd, *J* = 9.6, 4.4 Hz, 1H), 3.50 (dd, *J* = 13.6, 4.8), 3.15 (s, 3H), 2.87 (dd, *J* = 14.0, 10.0); ^13^C NMR (100 MHz, CDCl_3_, ppm) δ = 177.0, 144.2, 137.9, 129.4, 128.3, 127.9, 124.5, 122.0, 107.9, 47.0, 36.8, 26.1.

**1,​3-​Dihydro-​1-​phenyl-​3-​(phenylmethyl)​-2*H*-​indol-​2-​one** (**1v**)^11^

CAS registry No. [23226-10-0]. ^1^H NMR (400 MHz, CDCl_3_, ppm): δ = 7.39 (m, 2H), 7.29 (t, *J* = 13.6, 4.4 Hz, 1H), 7.21–6.85 (m, 10H), 6.57 (d, *J* = 8.4 Hz, 1H), 3.87–3.82 (m, 1H), 3.44 (dd, *J* = 13.6, 4.4 Hz, 1H), 3.09 (dd, *J* = 13.2, 8.0 Hz, 1H); ^13^C NMR (100 MHz, CDCl_3_, ppm) δ =176.4, 144.4, 137.3, 134.5, 129.6, 129.3, 128.2, 128.0, 127.8, 127.4, 126.7, 126.6, 124.7, 122.5, 109.2, 47.2, 37.1.

**1,​3Dihydro-​5-​methyl-​3-​(phenylmethyl)​-2*H*-​indol-​2-​one** (**1w**)^10^

CAS registry No. [861335-99-1]. ^1^H NMR (400 MHz, CDCl_3_, ppm): δ = 8.66 (brs, 1H), 7.27–7.16 (m, 5H), 6.95 (d, *J* = 7.2 Hz, 1H), 6.72 (d, *J* = 8.4 Hz, 1H), 6.56 (s, 1H), 3.70 (dd, *J* = 8.8, 4.8 Hz, 1H), 3.46 (dd, *J* = 13.6, 4.4 Hz, 1H), 2.93 (dd, *J* = 13.6, 6.8 Hz, 1H), 2.21 (s, 3H); ^13^C NMR (100 MHz, CDCl_3_, ppm) δ = 179.6, 138.9, 137.9, 131.4, 129.4, 129.1, 128.2, 126.6, 125.6, 109.3, 47.5, 36.6, 21.1.

**5-​Chloro-​1,​3-​dihydro-​3-​(phenylmethyl)​-2*H*-​indol-​2-​one** (**1x**)^6^

CAS registry No. [1165901-92-7]. ^1^H NMR (400 MHz, CDCl_3_, ppm): δ = 8.82 (brs, 1H), 7.28–7.21 (m, 3H), 7.17 (m, 3H), 6.74 (t, *J* = 8.0 Hz, 2H), 3.73 (dd, *J* = 8.8, 4.8 Hz, 1H), 3.46 (dd, *J* = 13.6, 9.2 Hz, 1H), 2.95 (*J* = 13.6, 9.2 Hz, 1H); ^13^C NMR (100 MHz, CDCl_3_, ppm) δ = 178.4, 139.7, 137.1, 130.6, 129.4, 128.5, 128.0, 127.4, 126.9, 125.3, 47.5, 36.4.

**Methyl-3-benzyl-2-oxoindoline-6-carboxylate** (**1y**)

^1^H NMR (400 MHz, CDCl_3_, ppm): δ = 7.45 (s, 1H), 7.26–7.21 (m, 4H), 7.15–7.13 (m, 3H), 6.84 (d, *J* = 7.2 Hz, 1H), 3.78 (dd, *J* = 8.8, 4.8 Hz, 1H), 3.50 (dd, *J* = 13.6, 4.0 Hz, 1H), 3.00 (*J* = 13.6, 8.8 Hz, 1H); ^13^C NMR (100 MHz, CDCl_3_, ppm) δ = 178.1, 166.6, 141.4, 137.1, 134.0, 130.1, 129.4, 128.4, 126.9, 124.7, 123.9, 110.0, 52.3, 47.6, 36.3.

**6-​Chloro-​1,​3-​dihydro-​3-​(phenylmethyl)​-2*H*-​indol-​2-​one** (**1z**)^13^

CAS registry No. [1352945-80-2]. ^1^H NMR (400 MHz, CDCl_3_, ppm): δ = 9.16 (brs, 1H), 7.28–7.15 (m, 5H), 6.87–6.85 (m, 2H), 6.61 (d, *J* = 8.0 Hz, 1H), 3.71 (dd, *J* = 9.2, 4.8 Hz, 1H), 3.47 (dd, *J* = 13.6, 4.4 Hz, 1H), 2.90 (*J* = 13.6, 9.6 Hz, 1H); ^13^C NMR (100 MHz, CDCl_3_, ppm) δ = 179.9, 142.6, 137.4, 133.7, 129.3, 128.4, 127.2, 126.8, 125.7, 122.0, 110.4, 47.2, 36.5.

**7. ^1^H NMR and ^13^C NMR spectra of products**

**
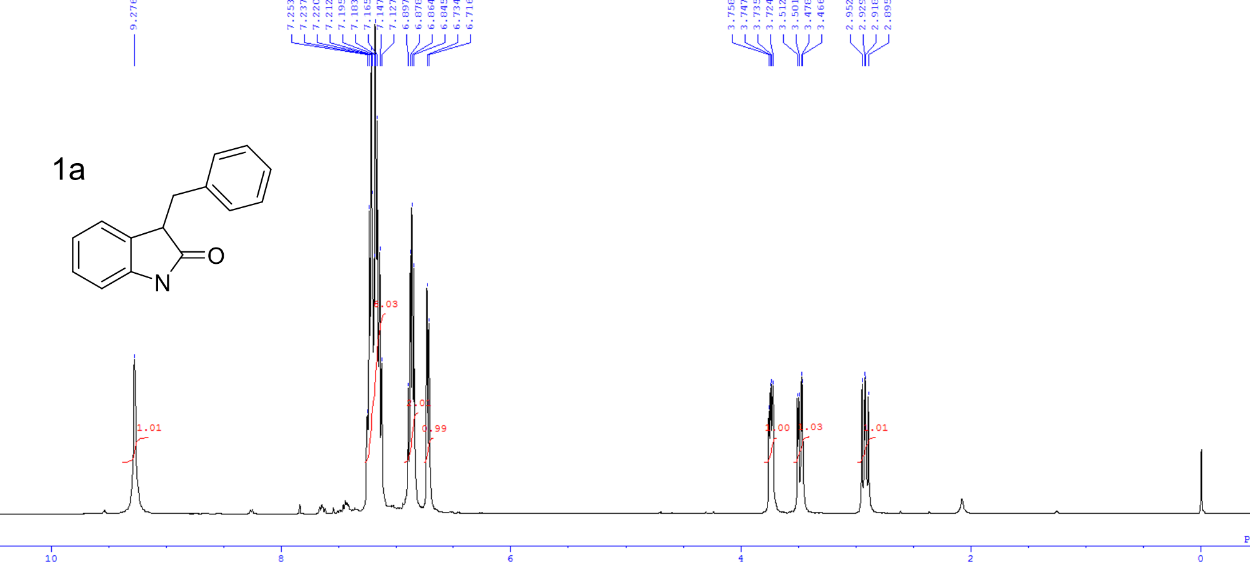
**

^1^H NMR spectrum of **1a**

**
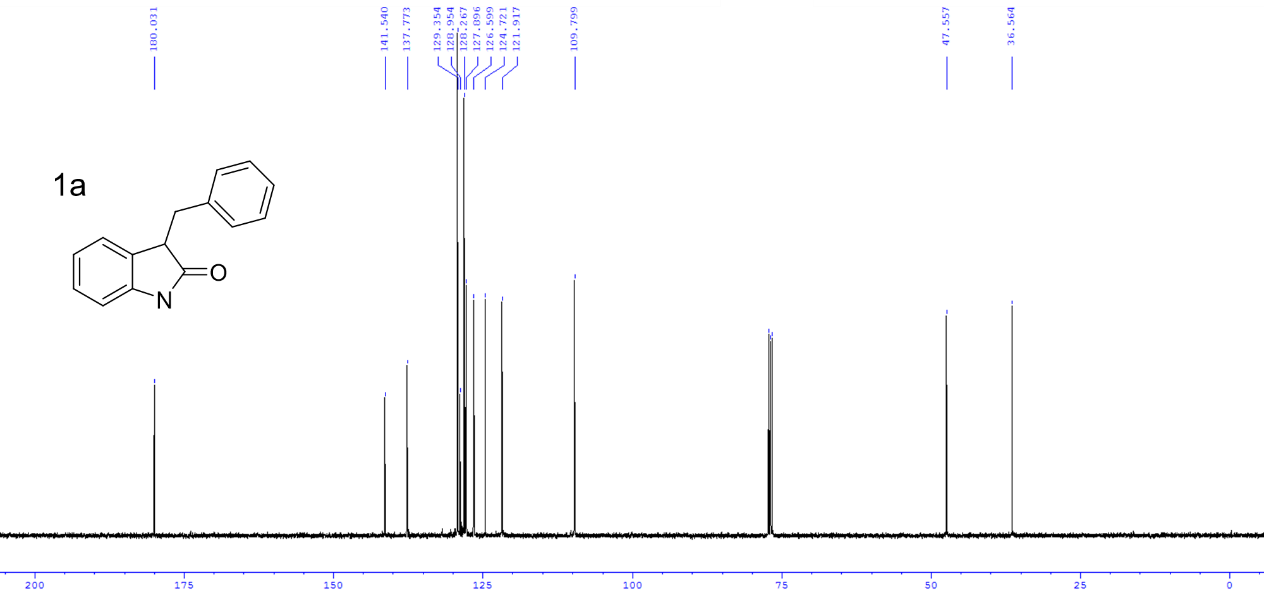
**

^13^C NMR spectrum of **1a**

**
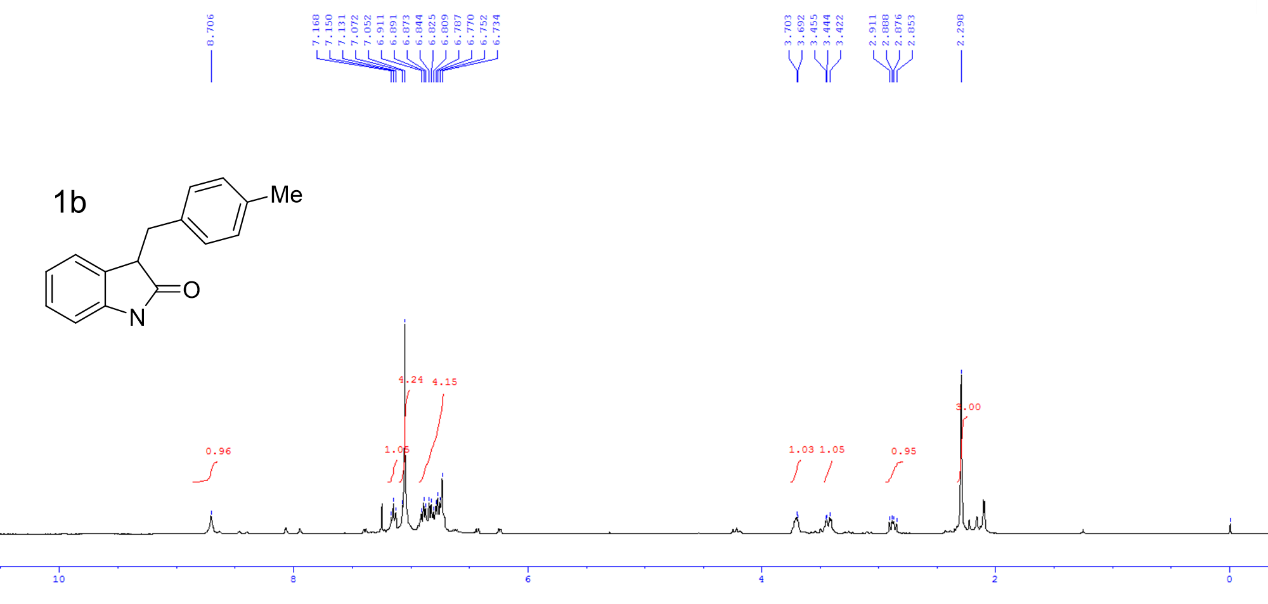
**

^1^H NMR spectrum of **1b**

**
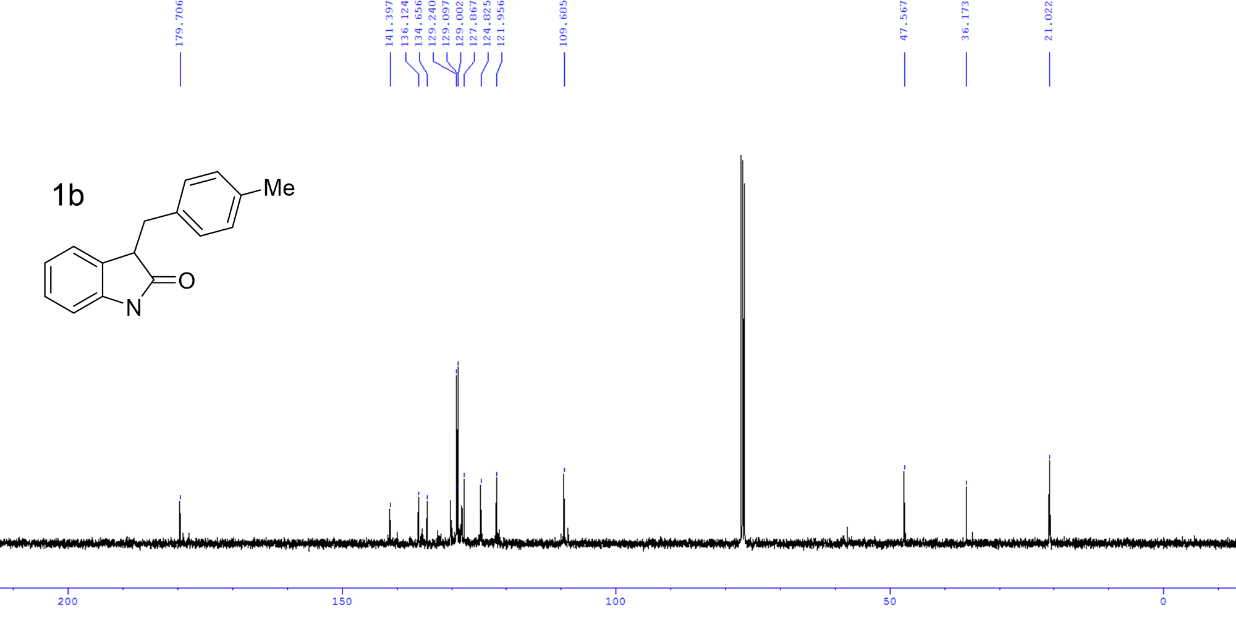
**

^13^C NMR spectrum of **1b**

**
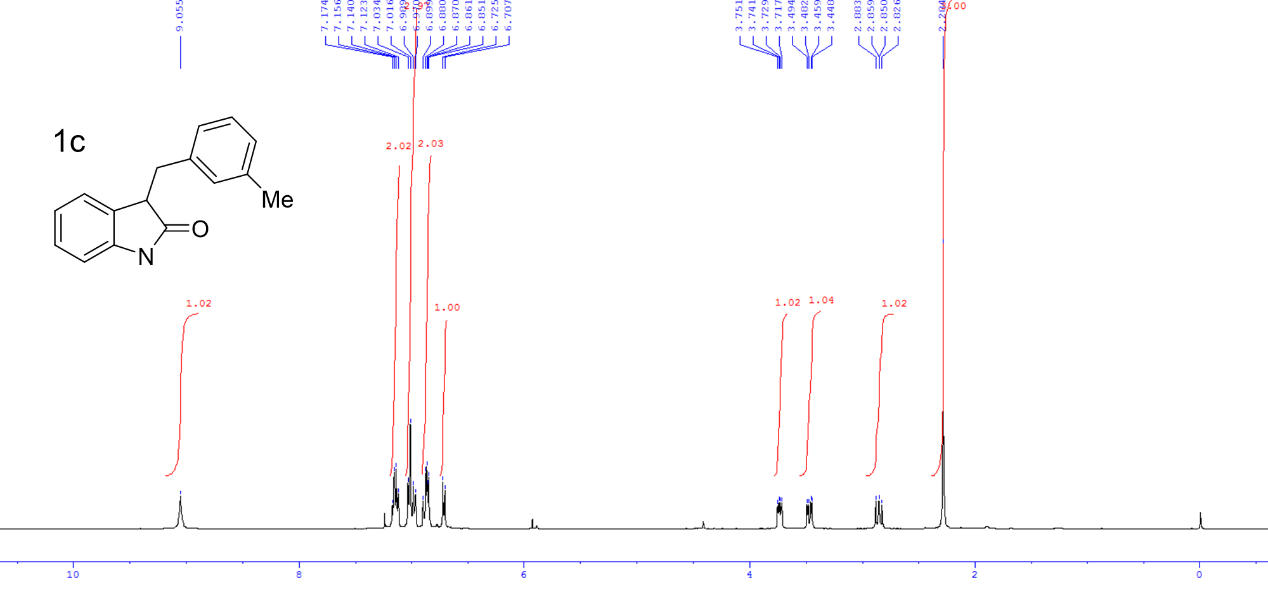
**

^1^H NMR spectrum of **1c**

**
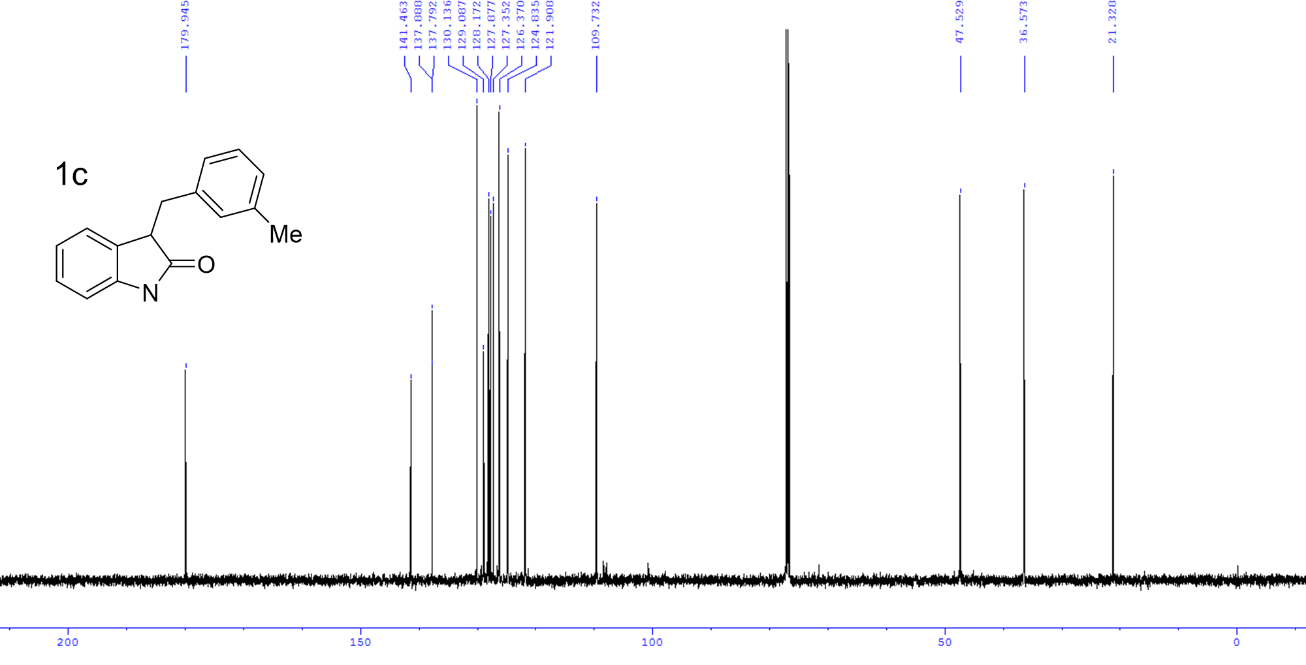
**

^13^C NMR spectrum of **1c**

**
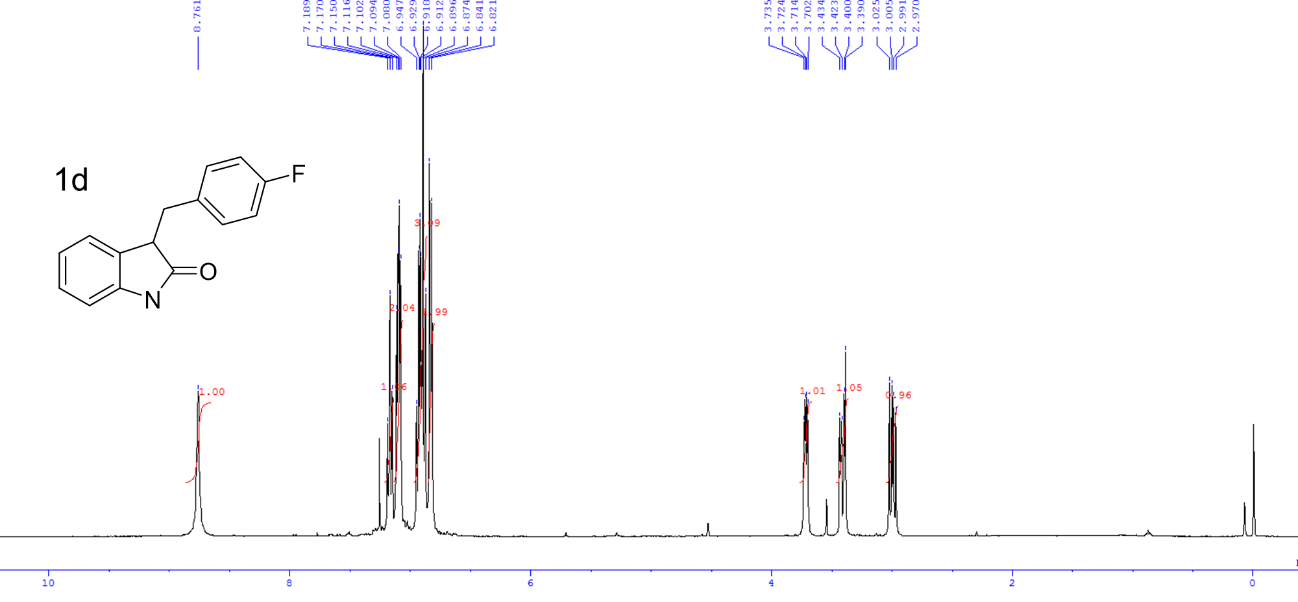
**

^1^H NMR spectrum of **1d**

**
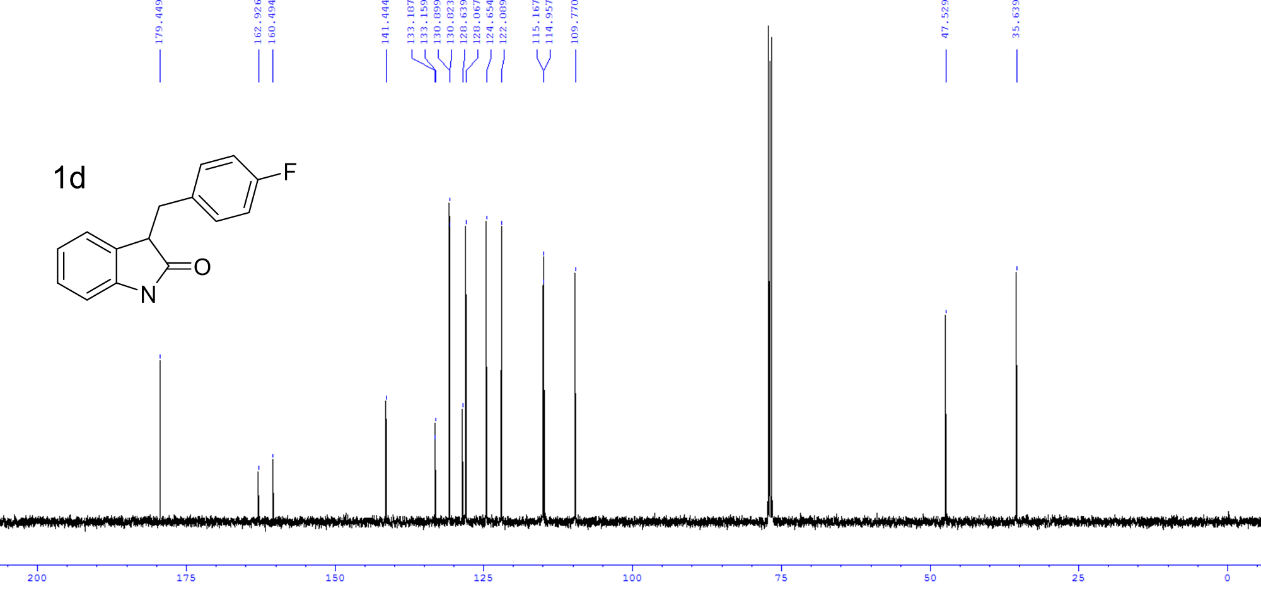
**

^13^C NMR spectrum of **1d**

**
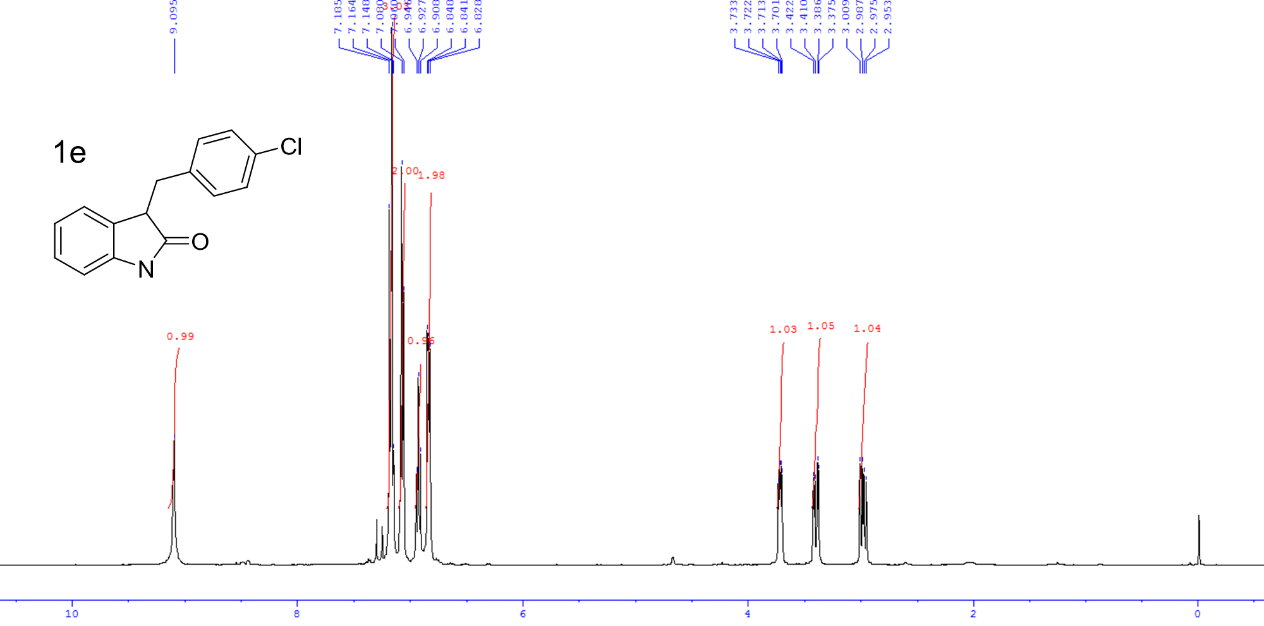
**

^1^H NMR spectrum of **1e**

**
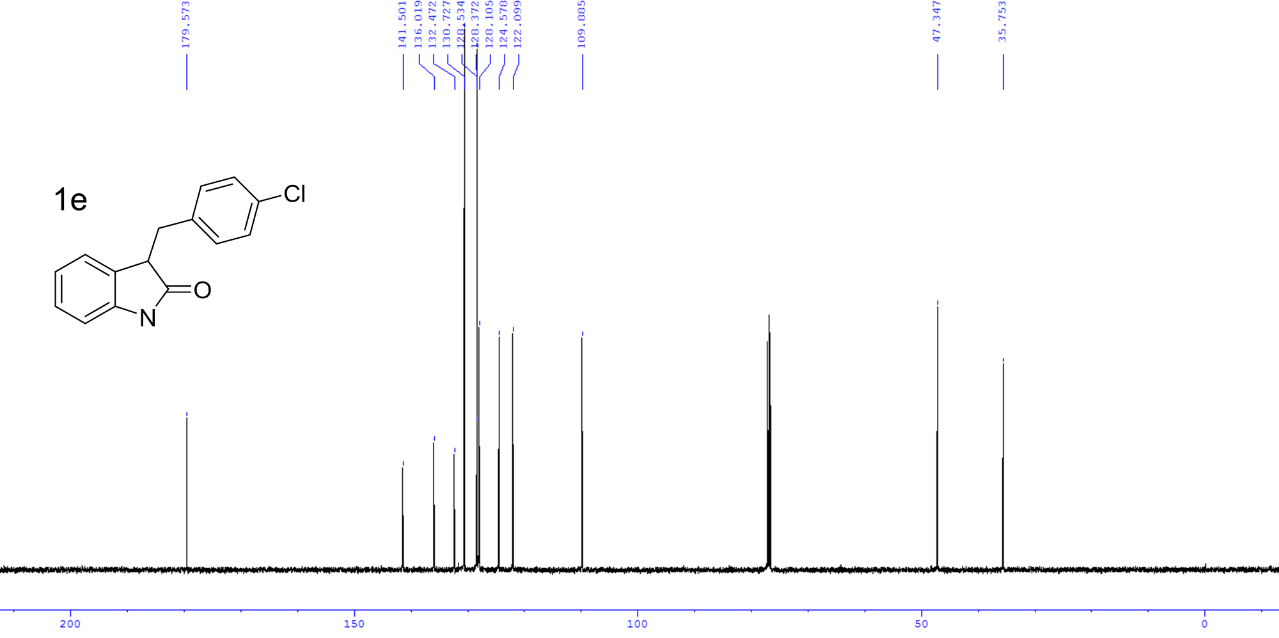
**

^13^C NMR spectrum of **1e**

**
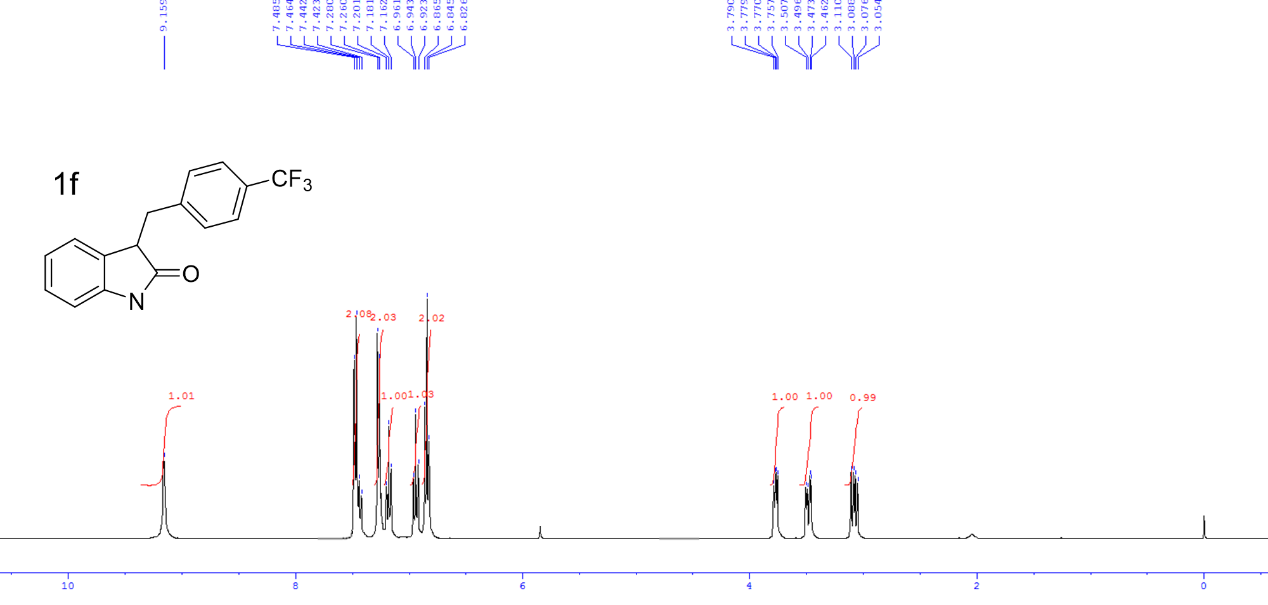
**

^1^H NMR spectrum of **1f**

**
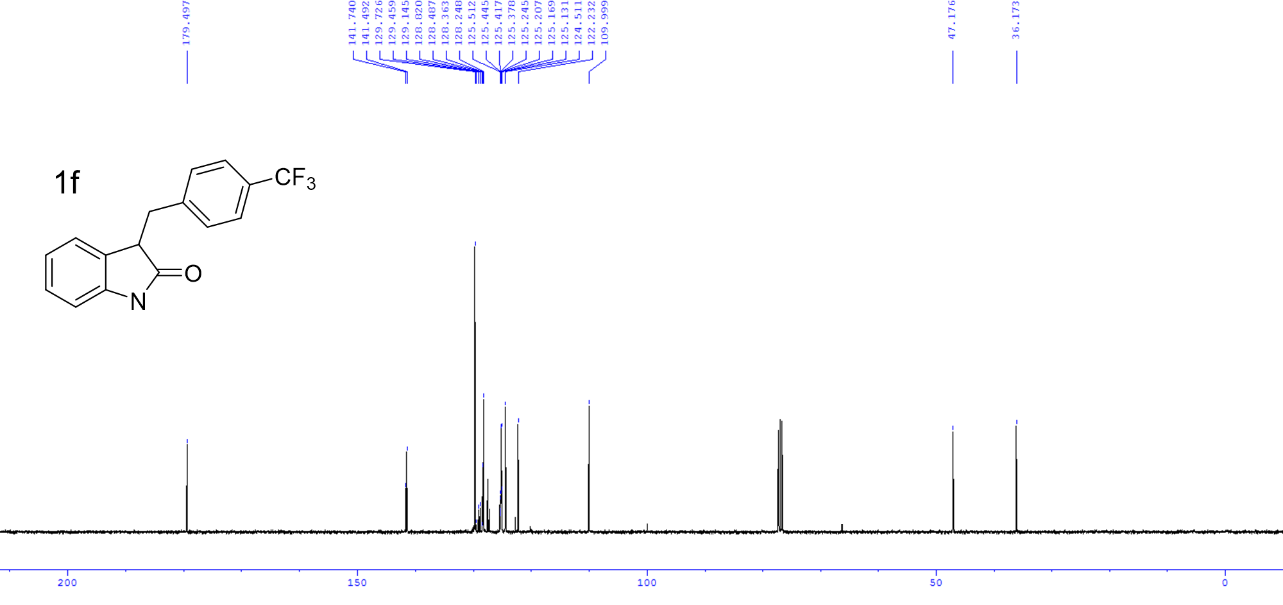
**

^13^C NMR spectrum of **1f**

**
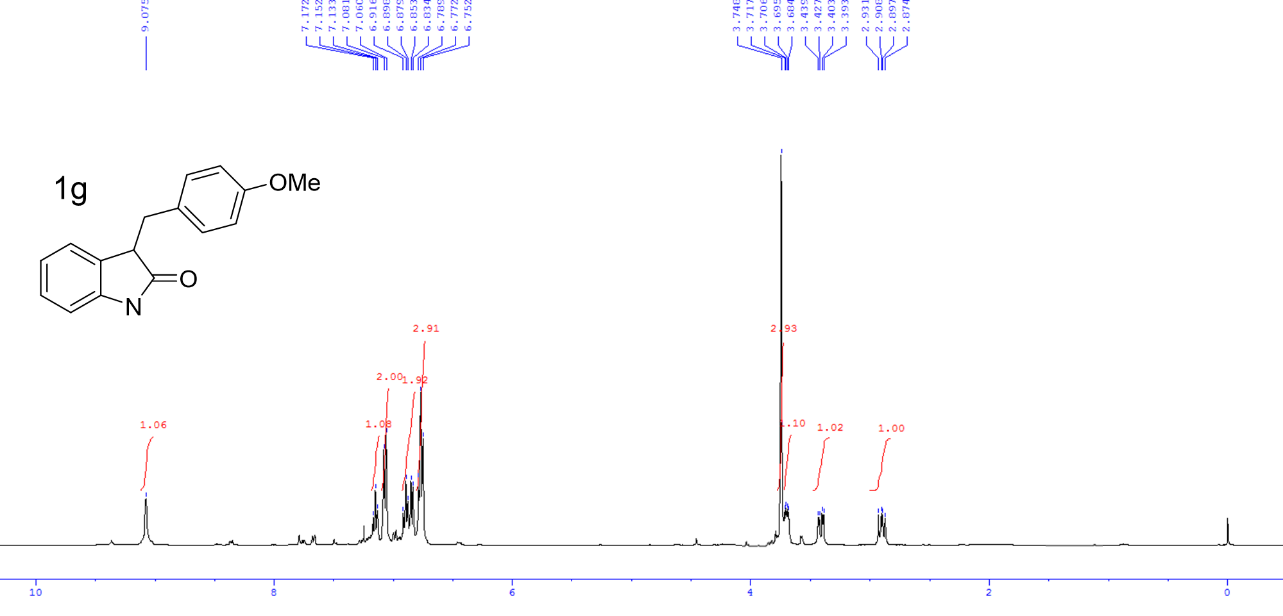
**

^1^H NMR spectrum of **1g**

**
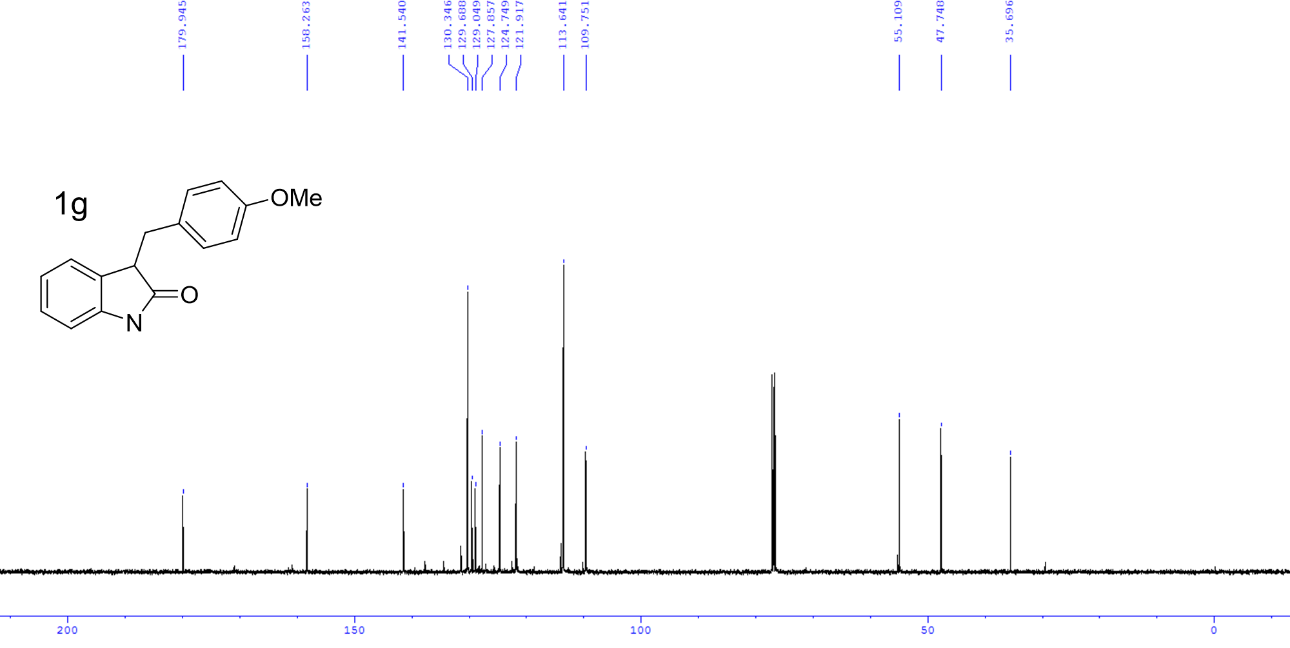
**

^13^C NMR spectrum of **1g**

**
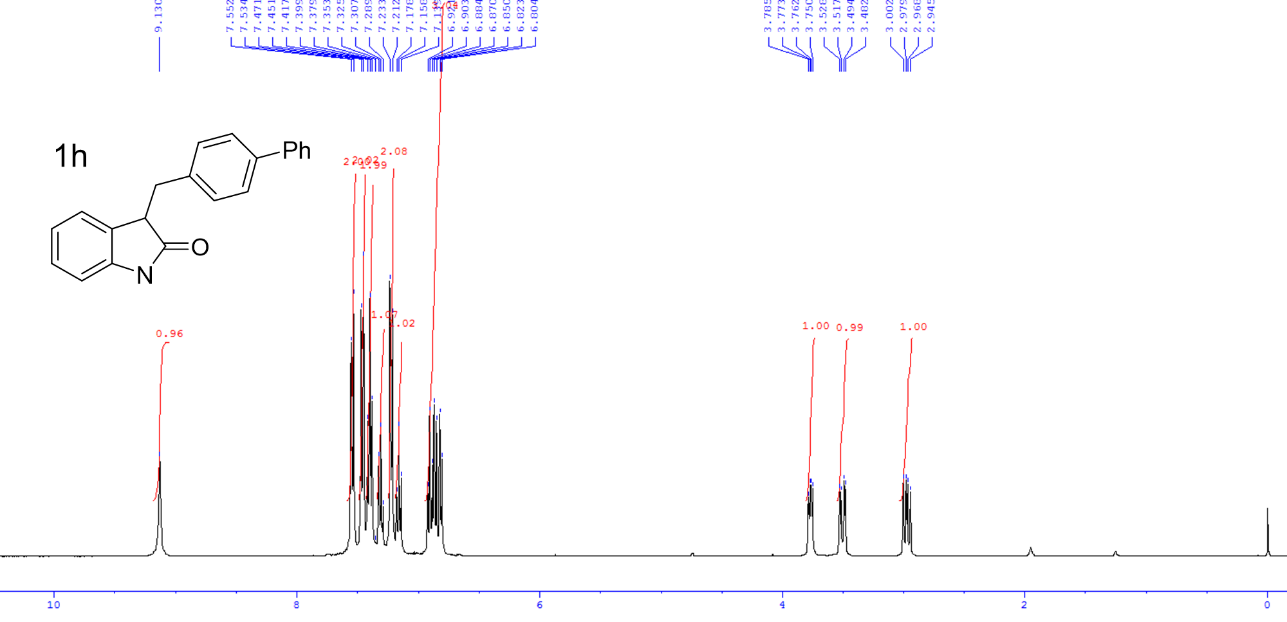
**

^1^H NMR spectrum of **1h**

**
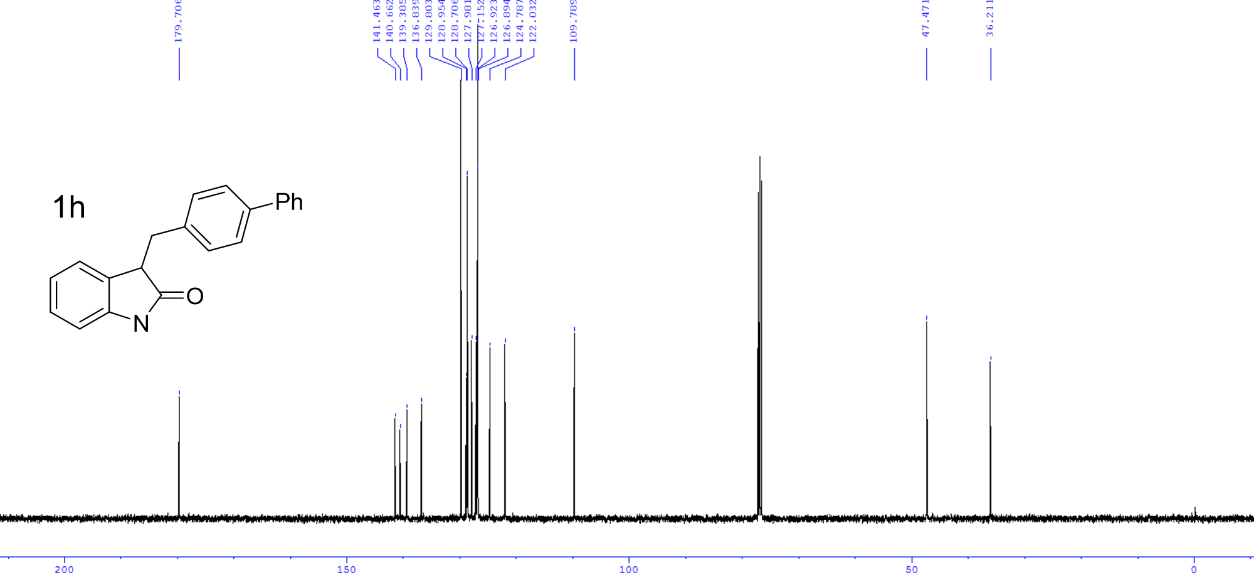
**

^13^C NMR spectrum of **1h**

**
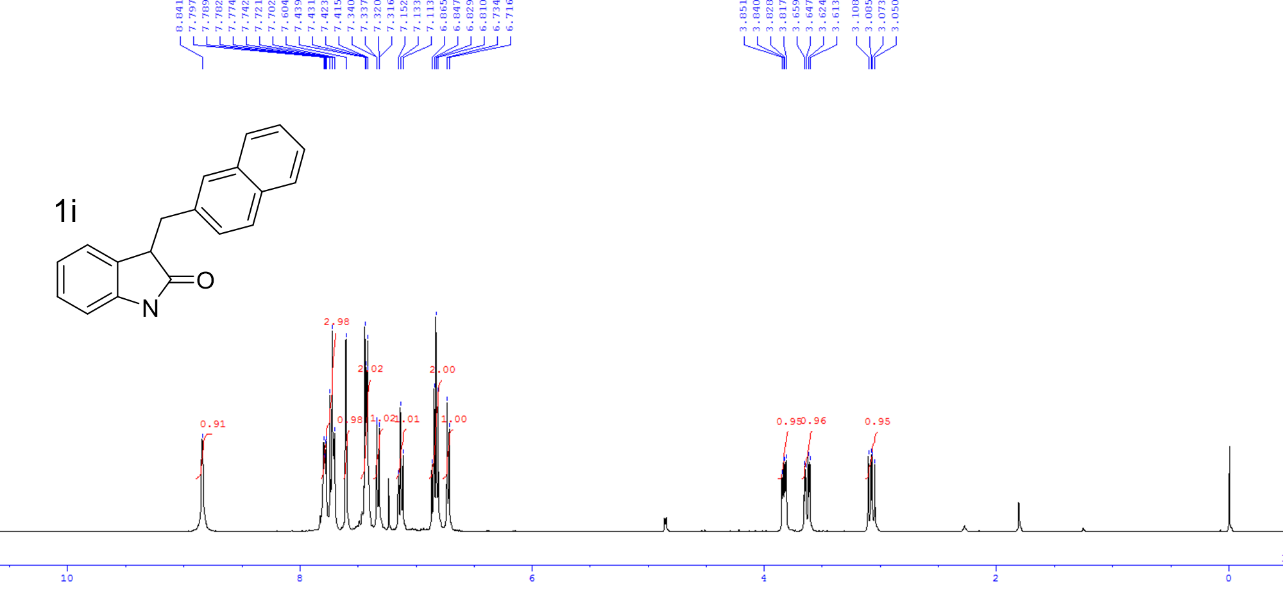
**

^1^H NMR spectrum of **1i**

**
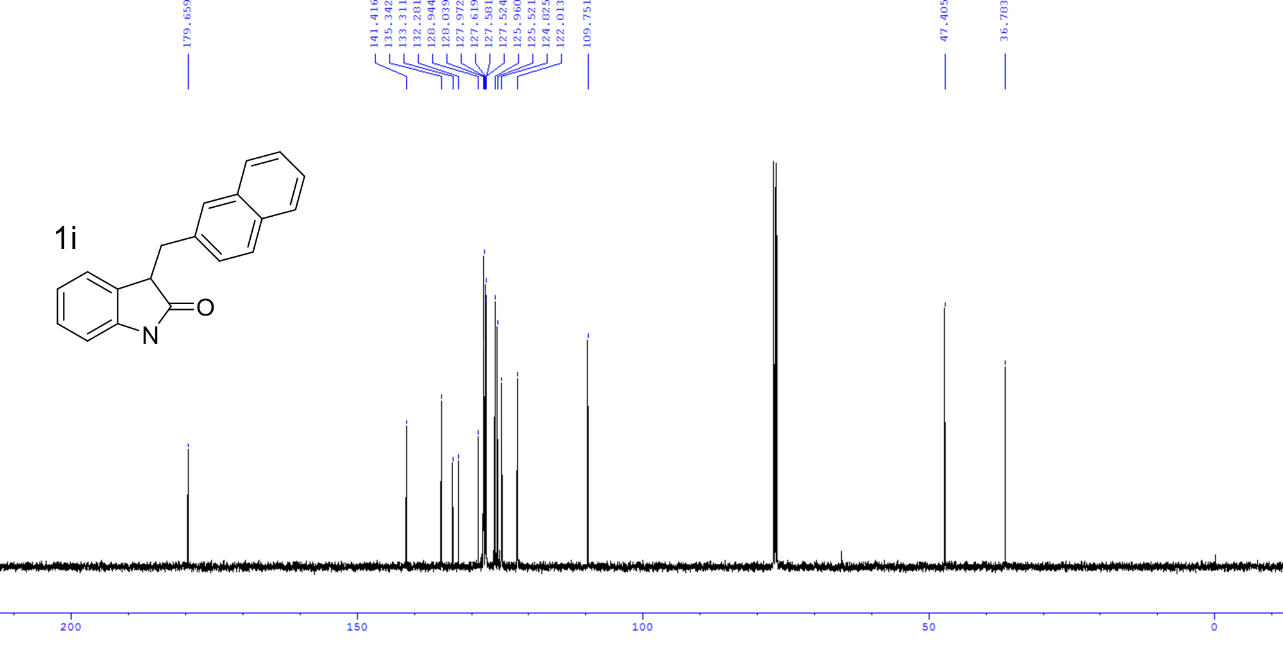
**

^13^C NMR spectrum of **1i**

**
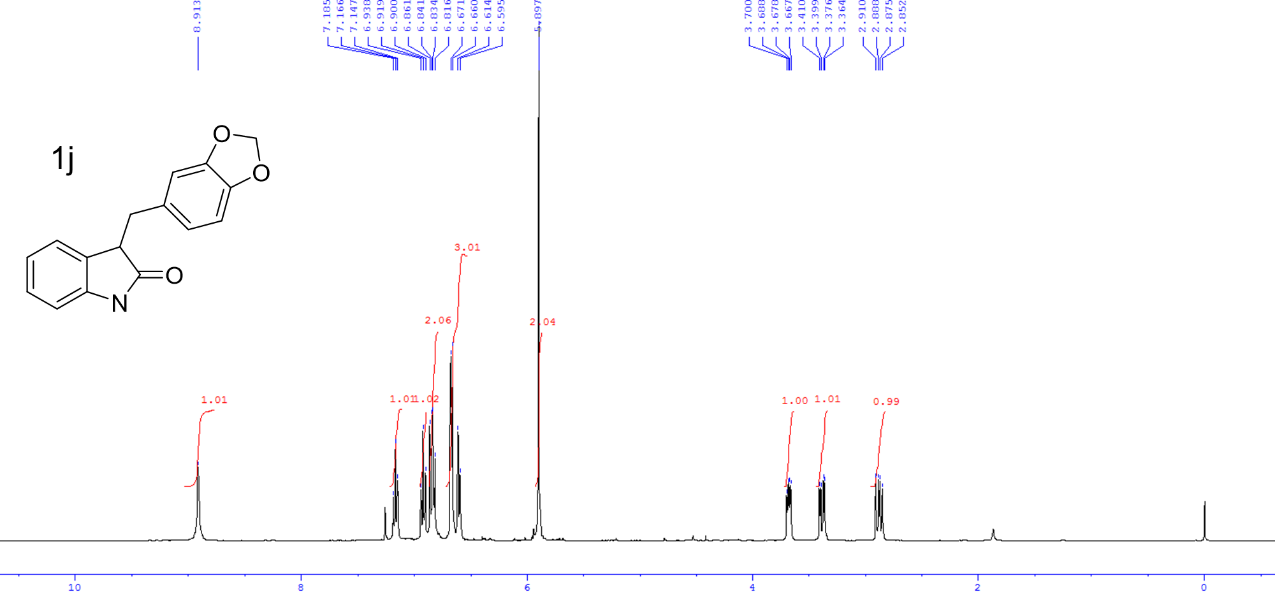
**

^1^H NMR spectrum of **1j**

**
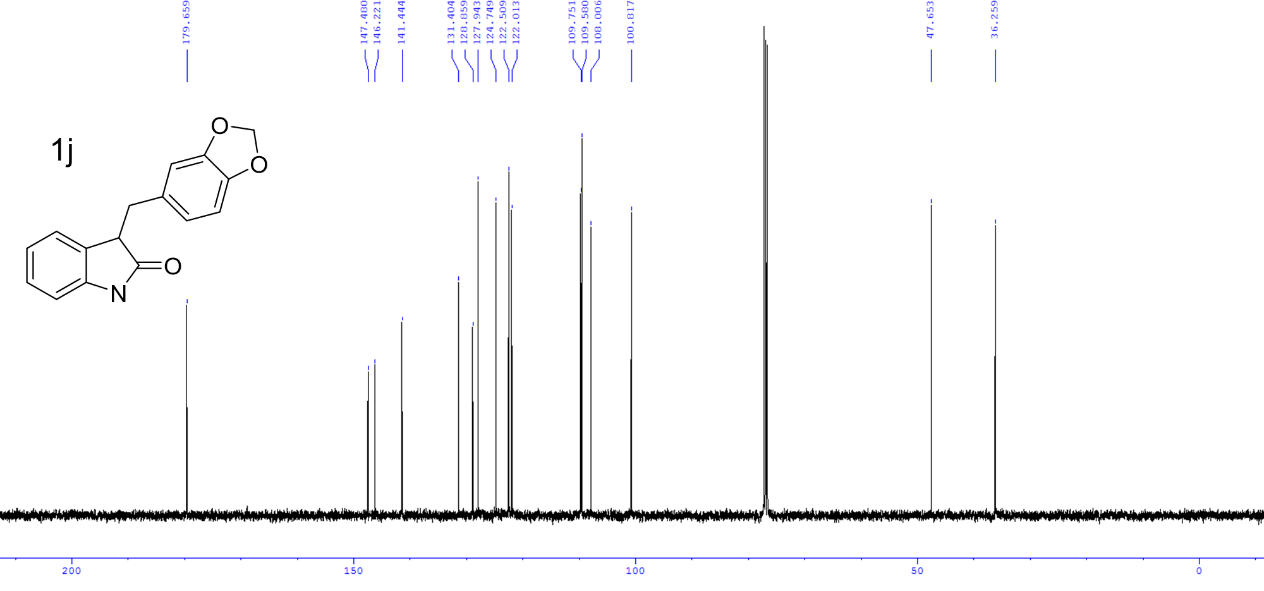
**

^13^C NMR spectrum of **1j**

**
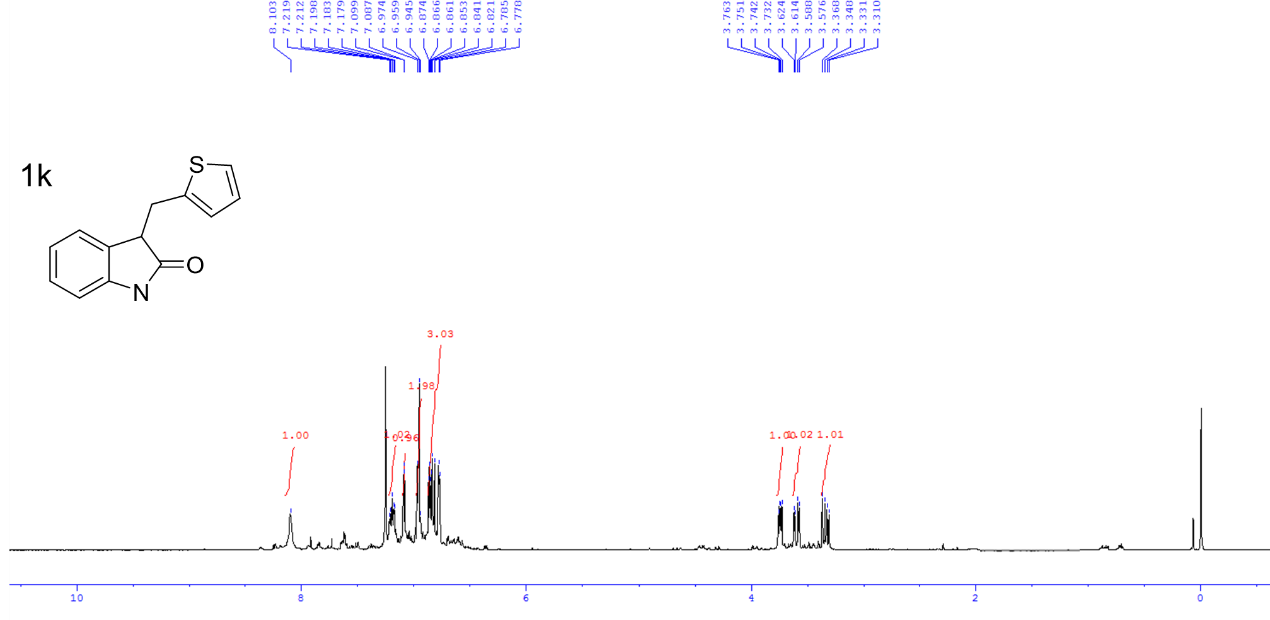
**

^1^H NMR spectrum of **1k**

**
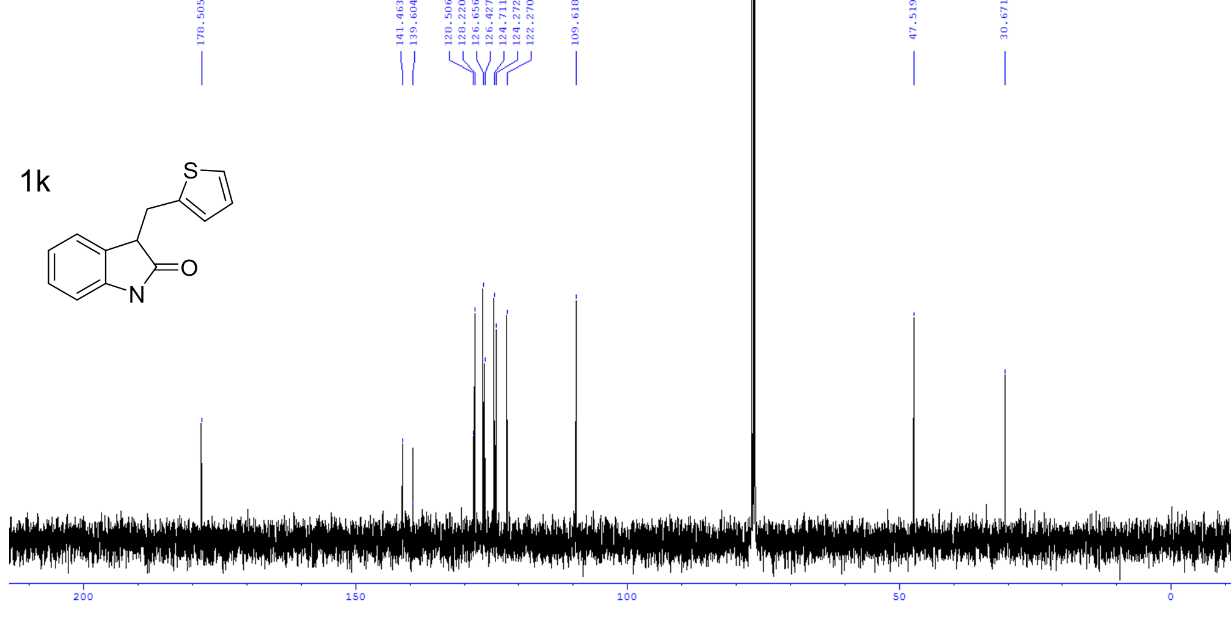
**

^13^C NMR spectrum of **1k**

**
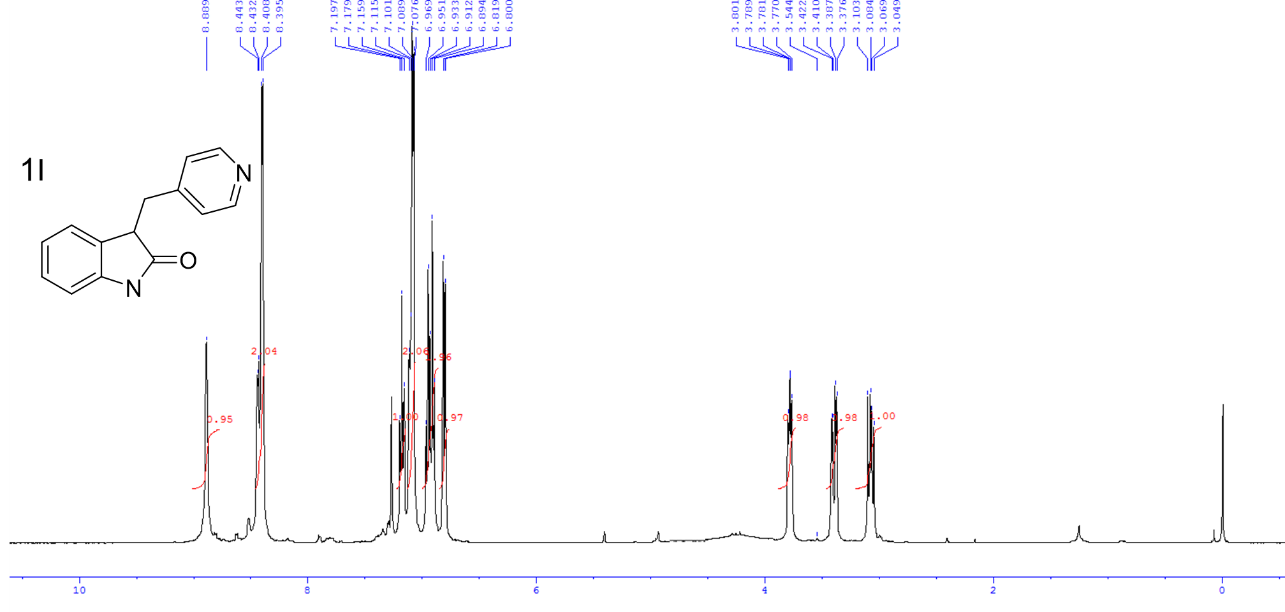
**

^1^H NMR spectrum of **1l**

**
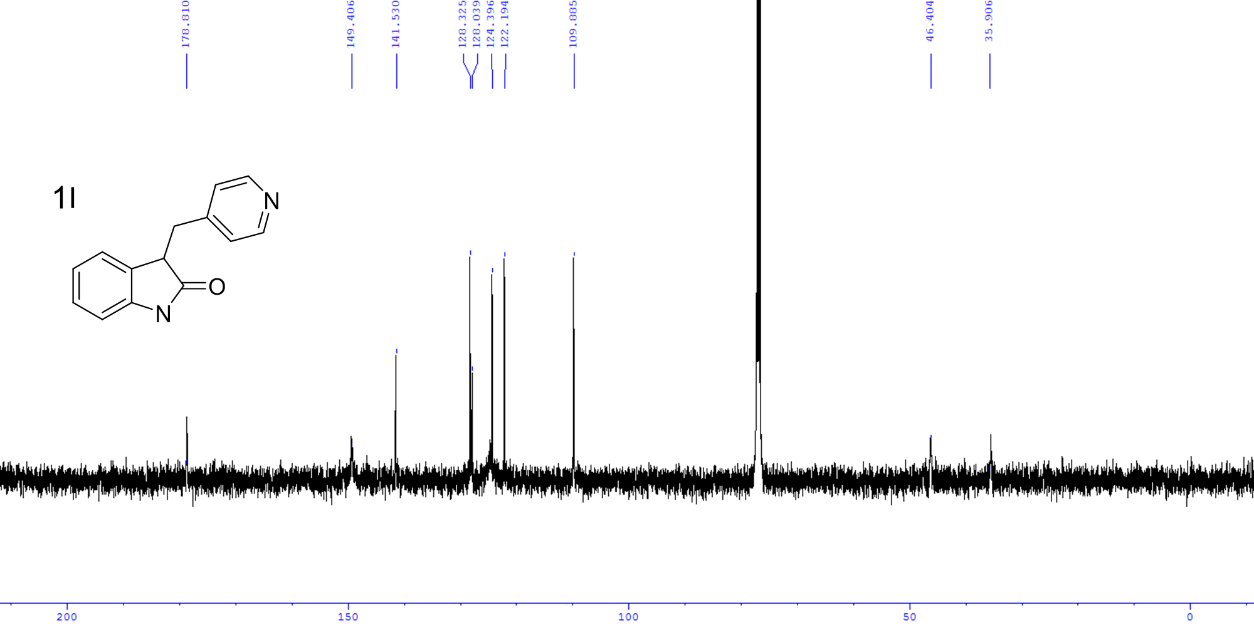
**

^13^C NMR spectrum of **1l**

**
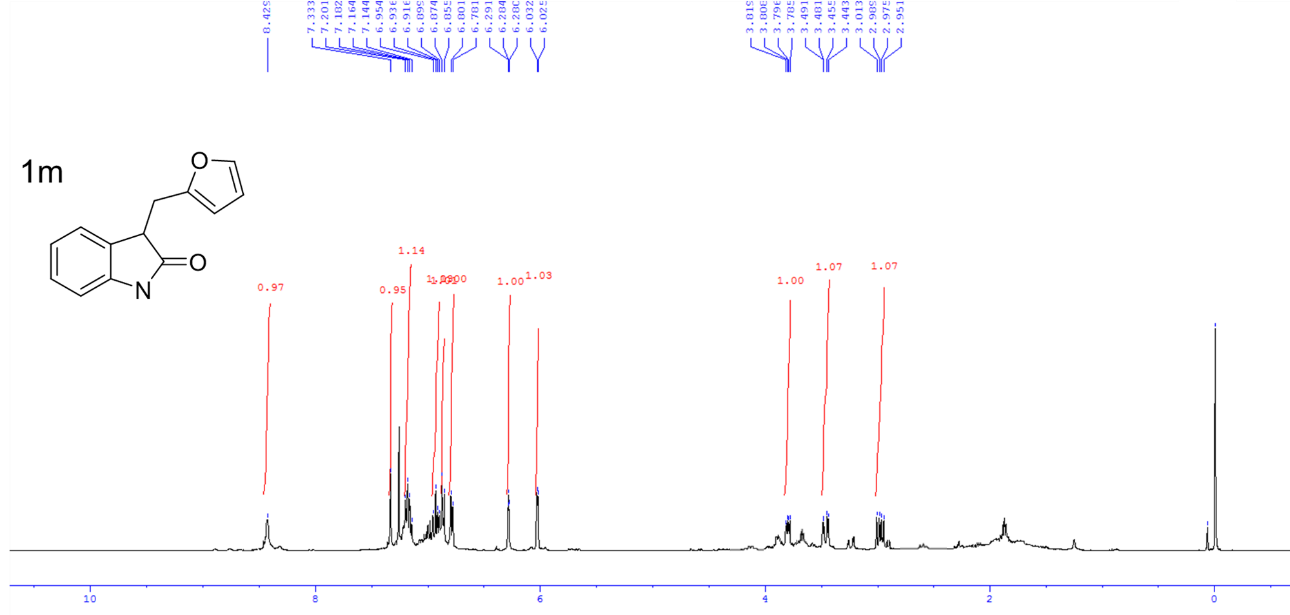
**

^1^H NMR spectrum of **1m**

**
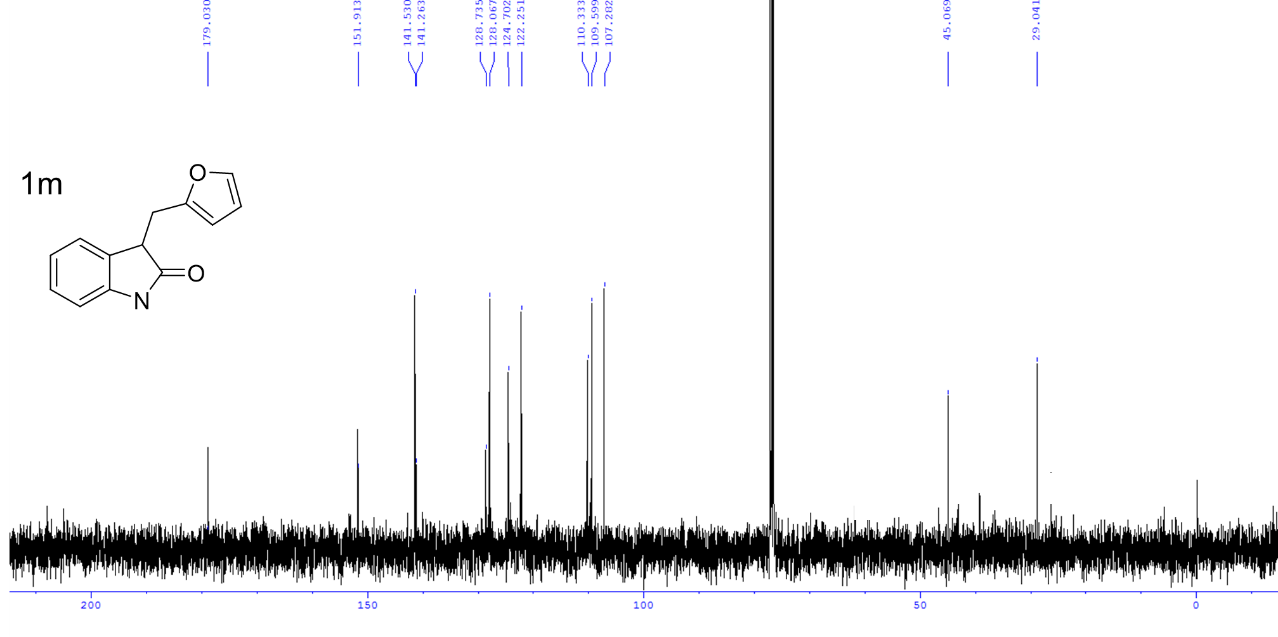
**

^13^C NMR spectrum of **1m**

**
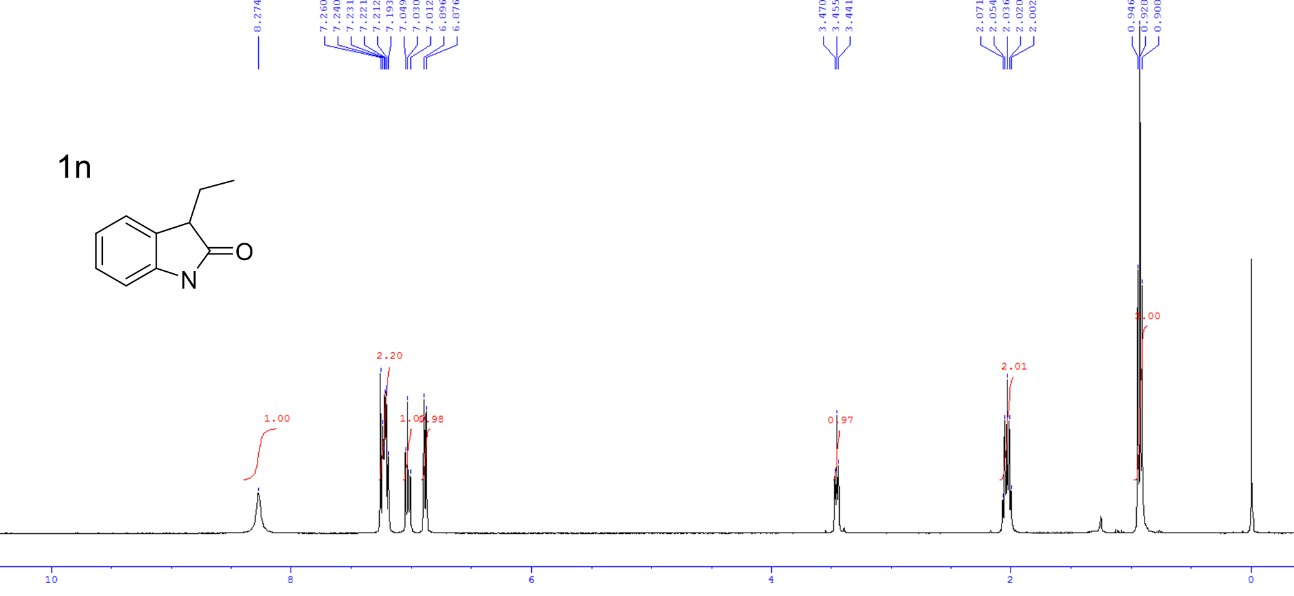
**

^1^H NMR spectrum of **1n**

**
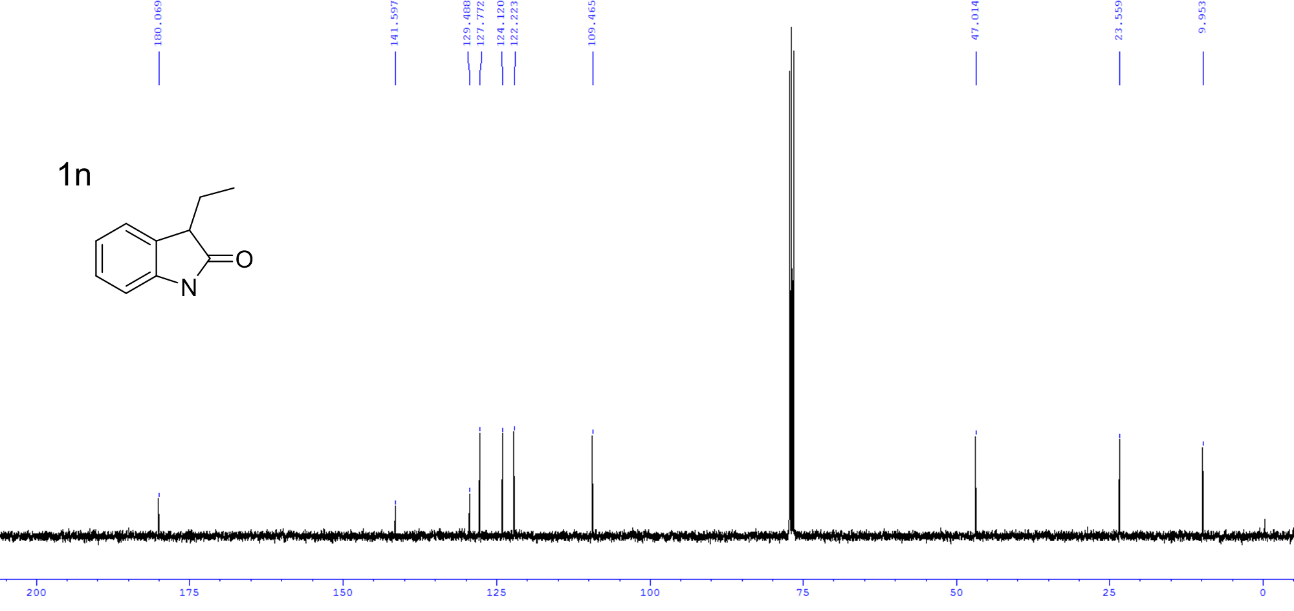
**

^13^C NMR spectrum of **1n**

**
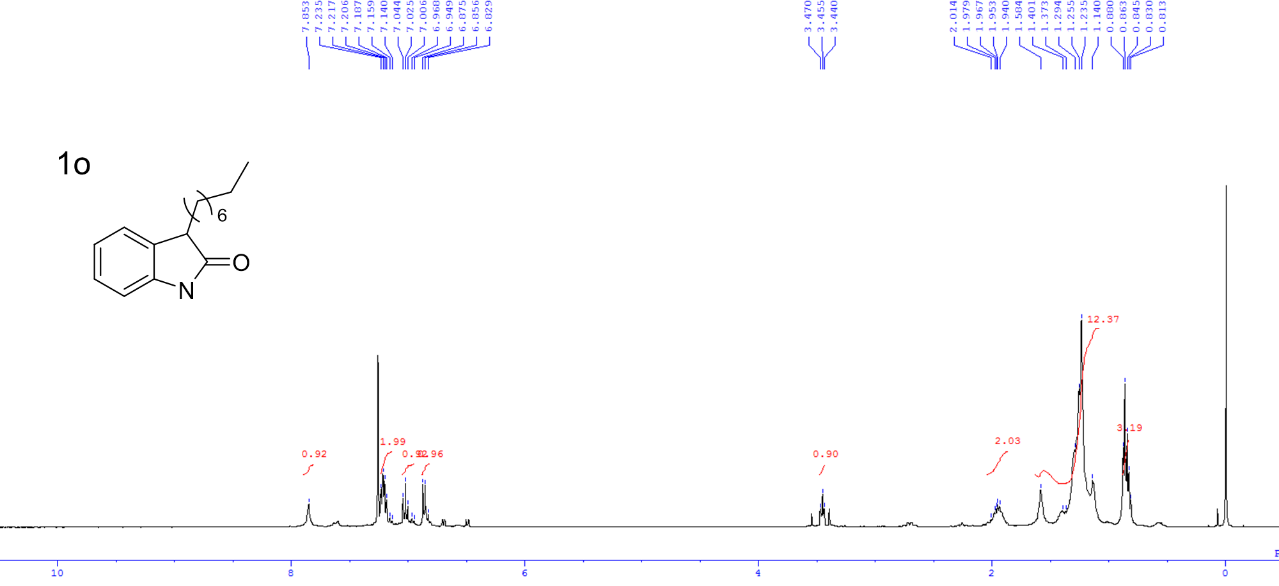
**

^1^H NMR spectrum of **1o**

**
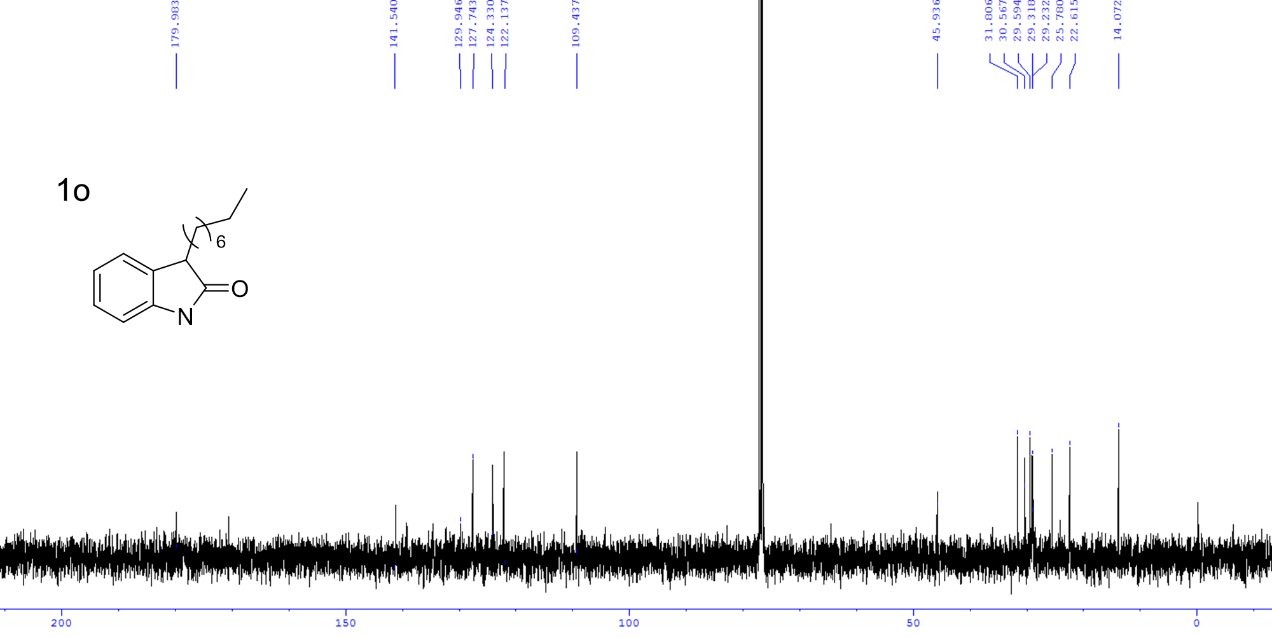
**

^13^C NMR spectrum of **1o**

**
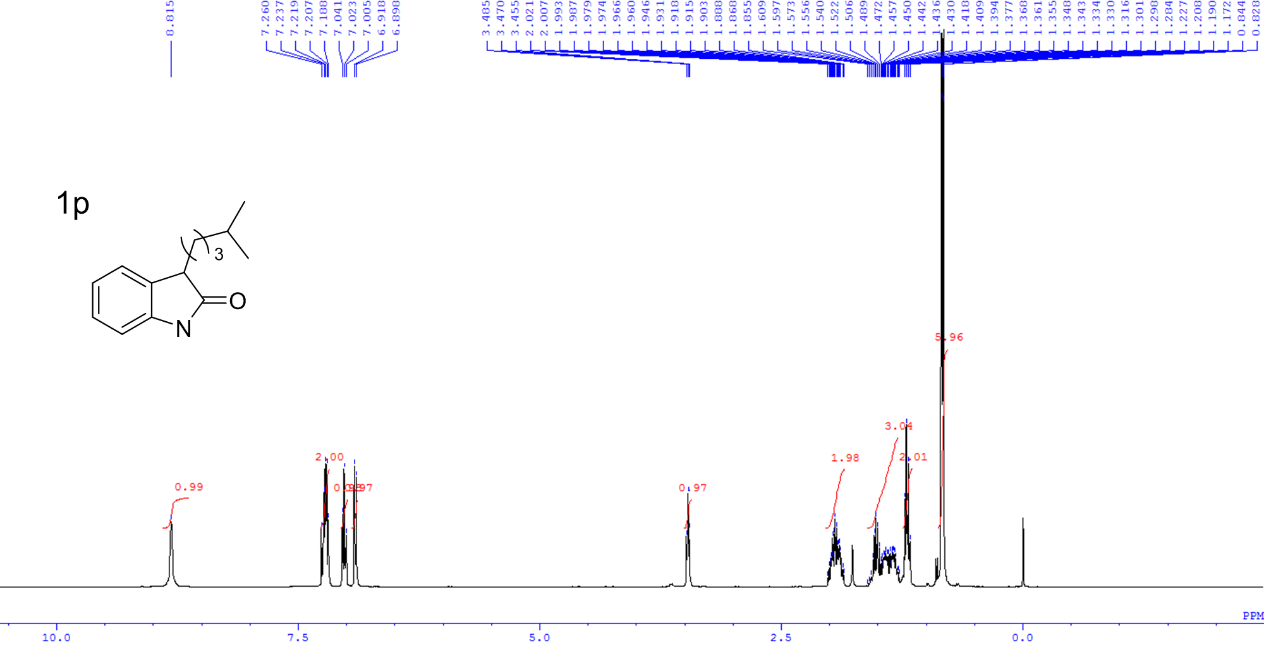
**

^1^H NMR spectrum of **1p**

**
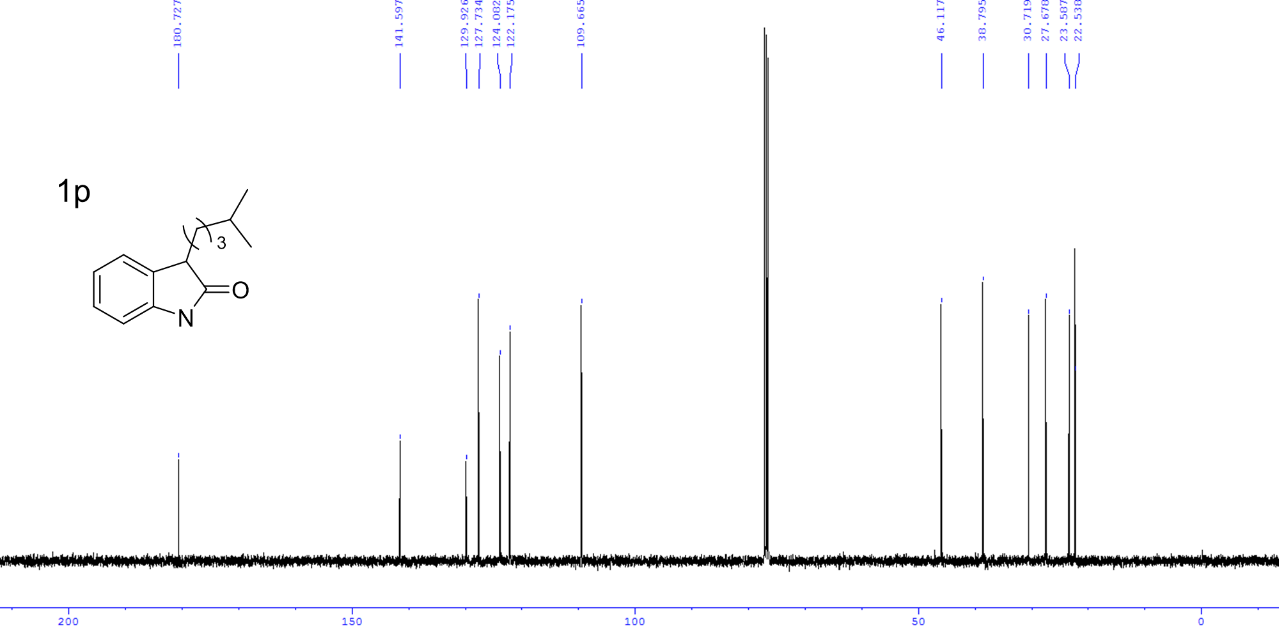
**

^13^C NMR spectrum of **1p**

**
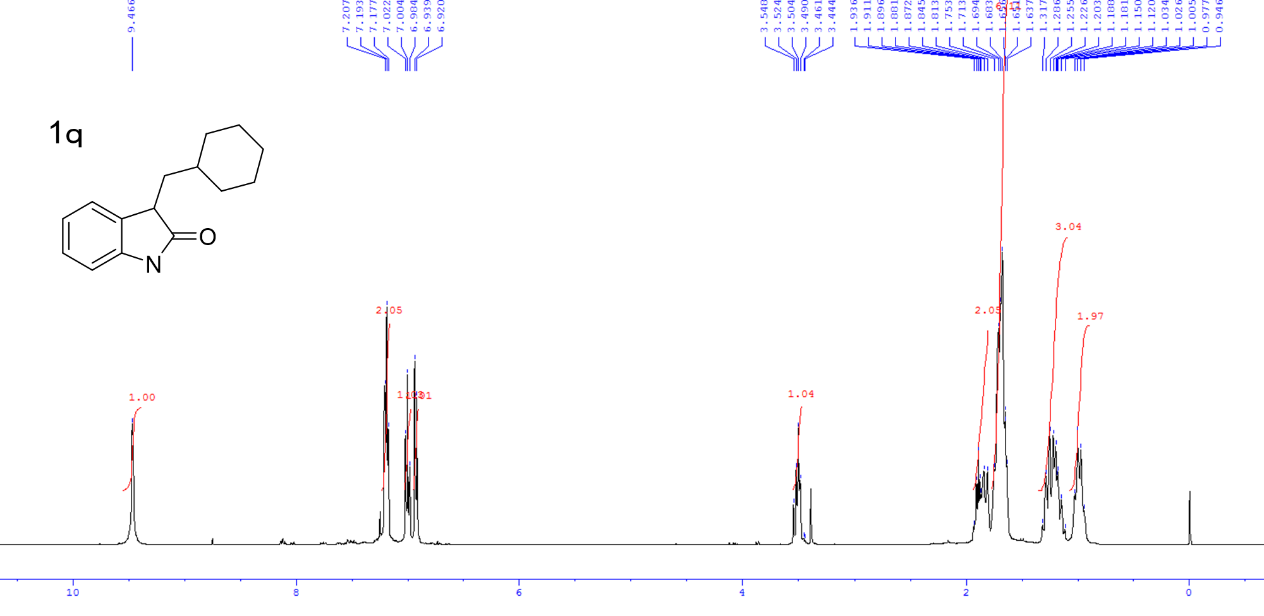
**

^1^H NMR spectrum of **1q**

**
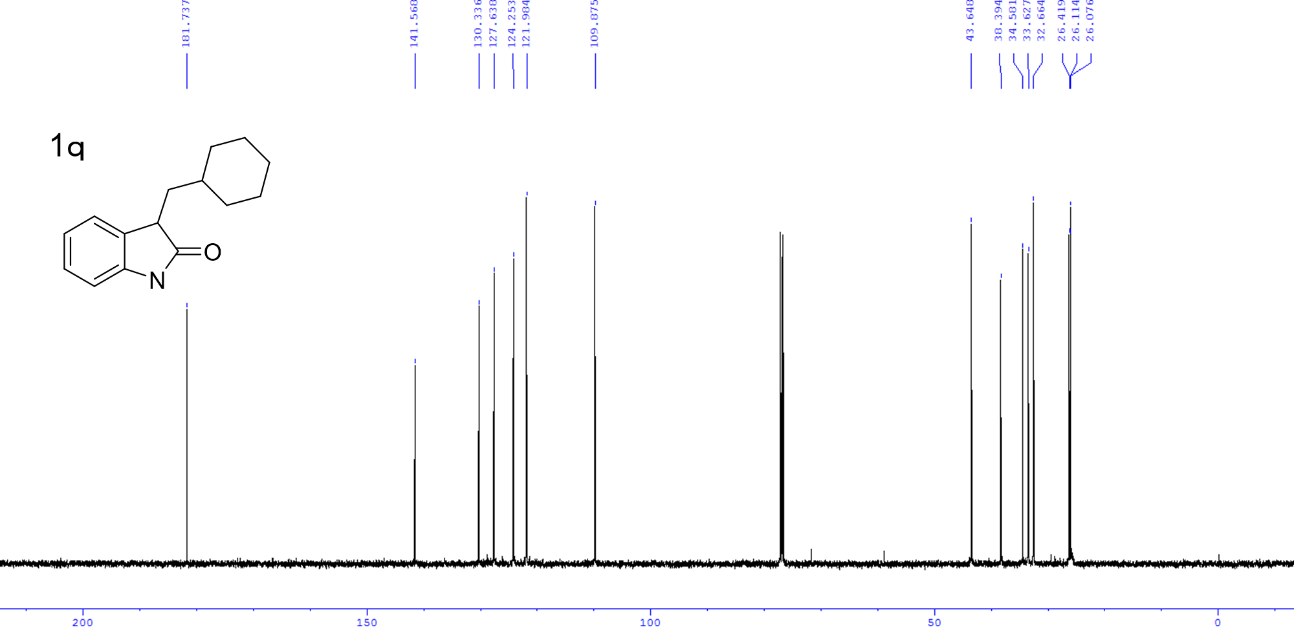
**

^13^C NMR spectrum of **1q**

**
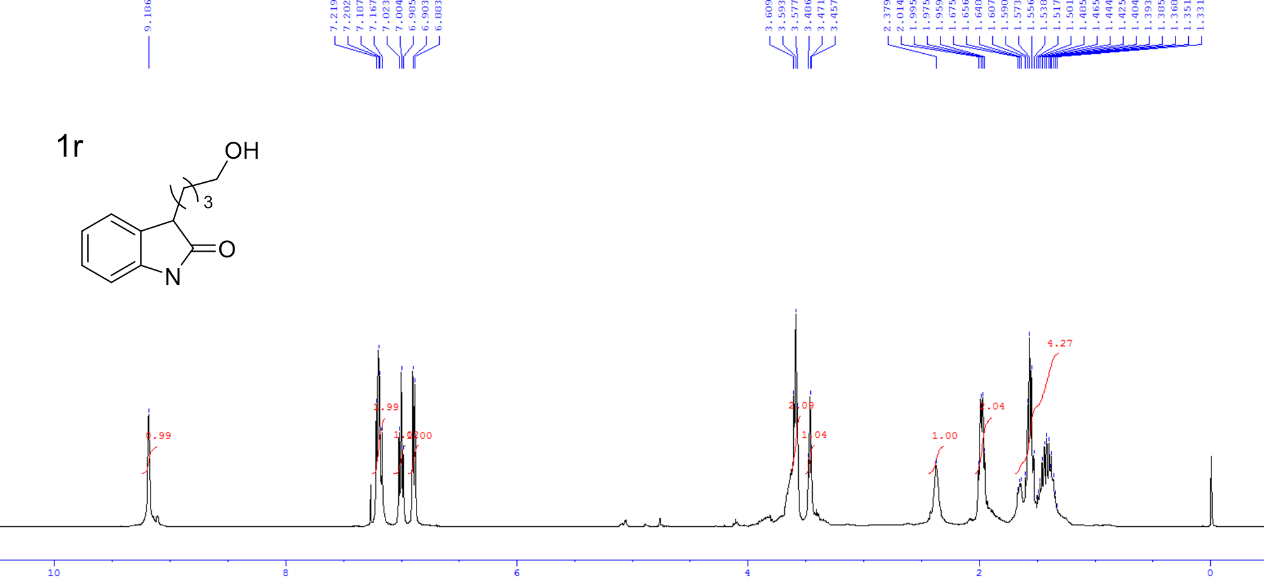
**

^1^H NMR spectrum of **1r**

**
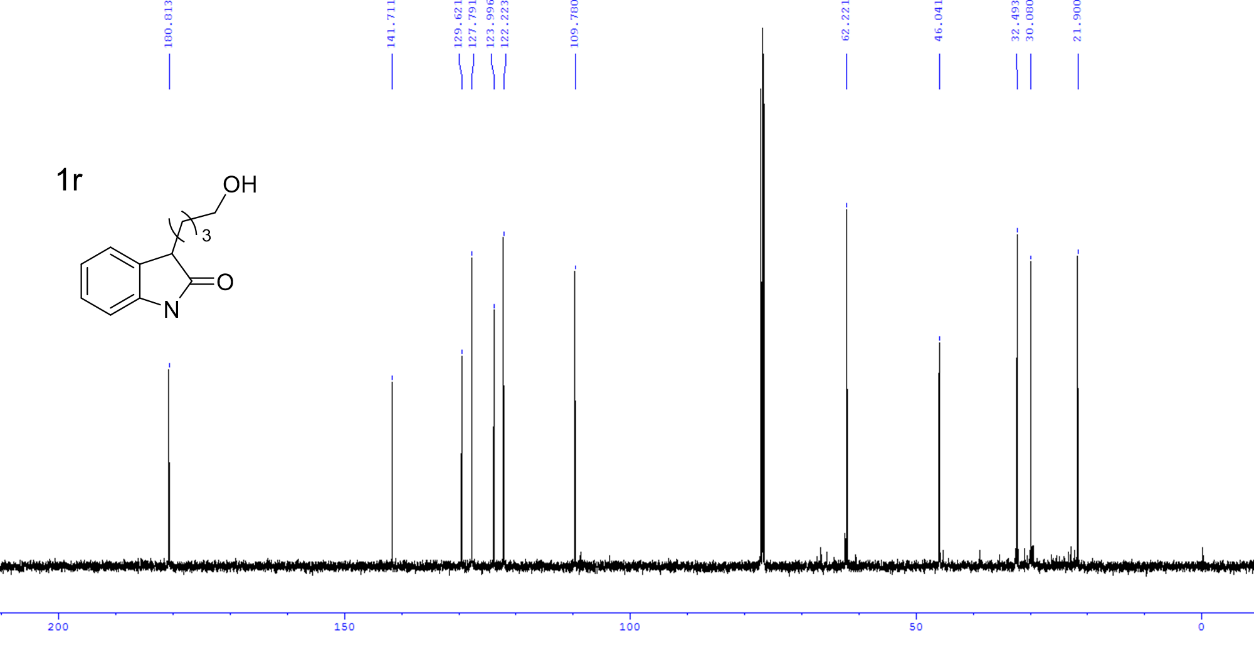
**

^13^C NMR spectrum of **1r**

**
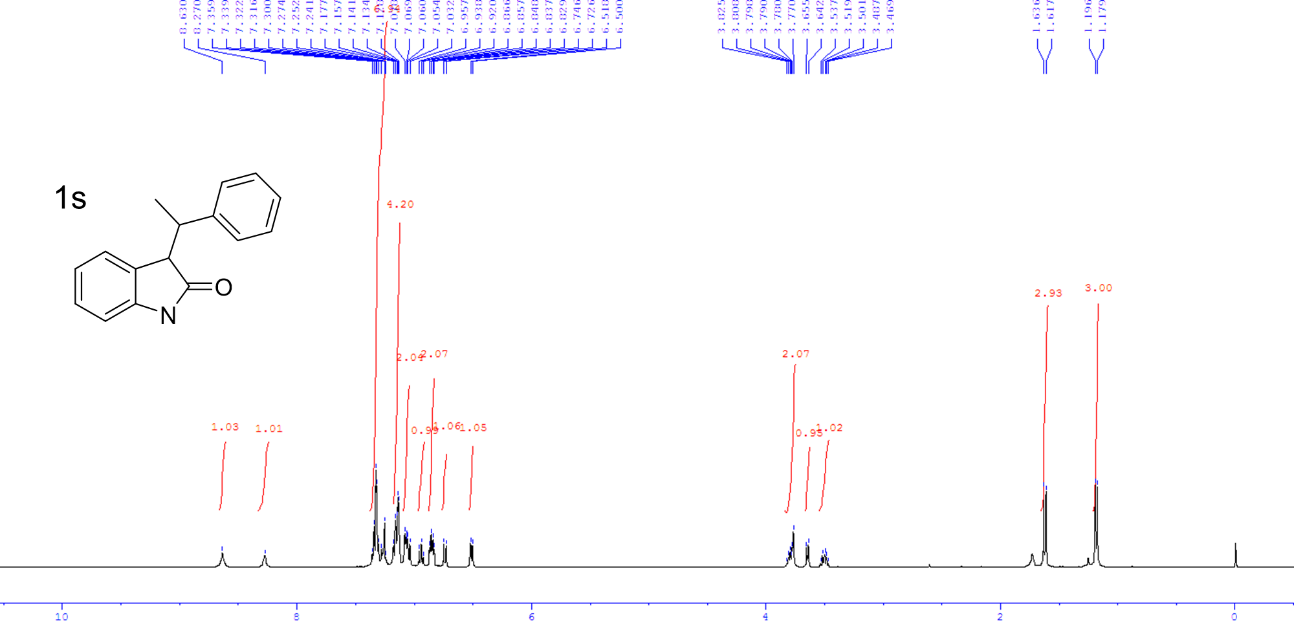
**

^1^H NMR spectrum of **1s**

**
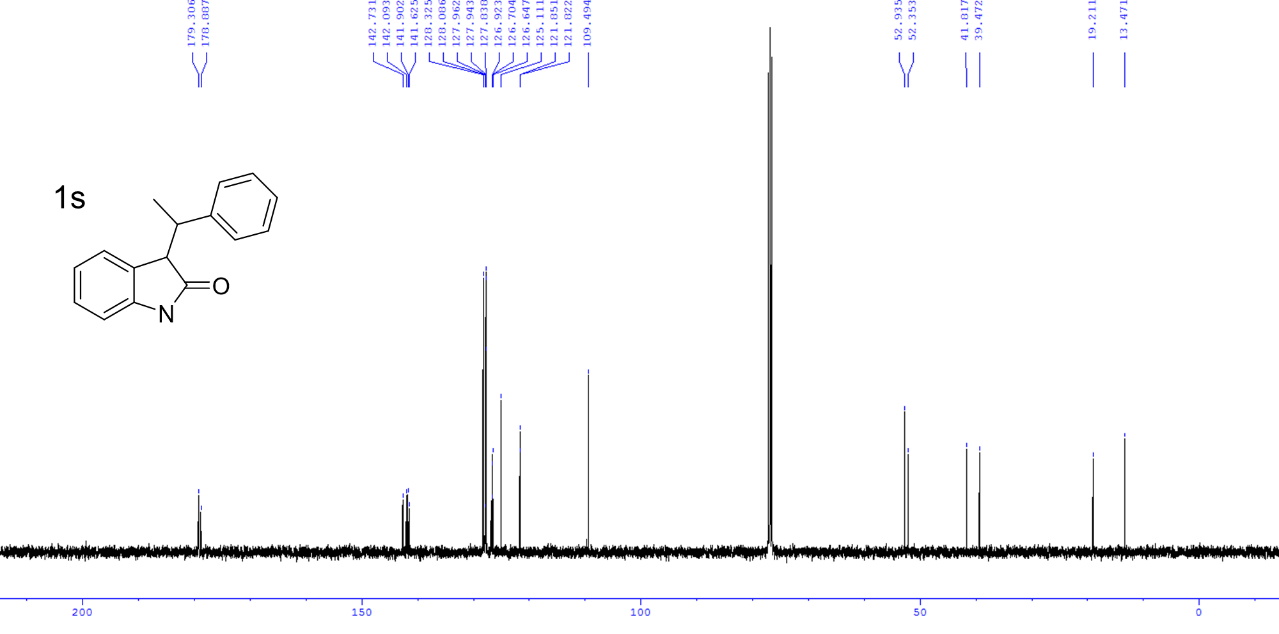
**

^13^C NMR spectrum of **1s**

**
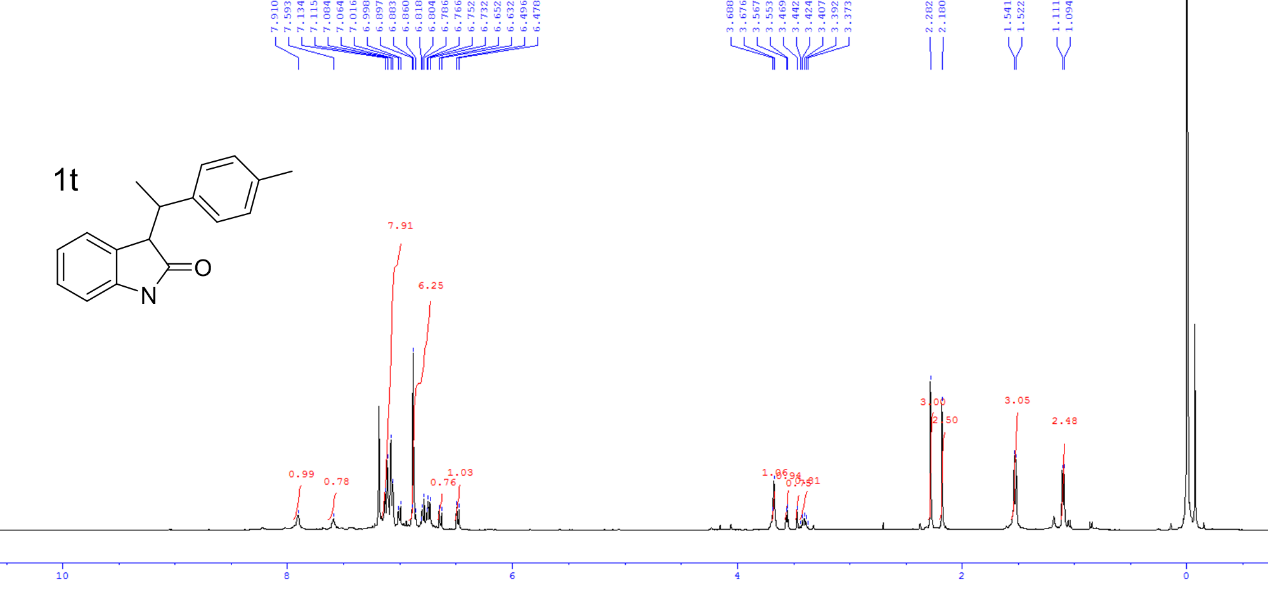
**

^1^H NMR spectrum of **1t**

**
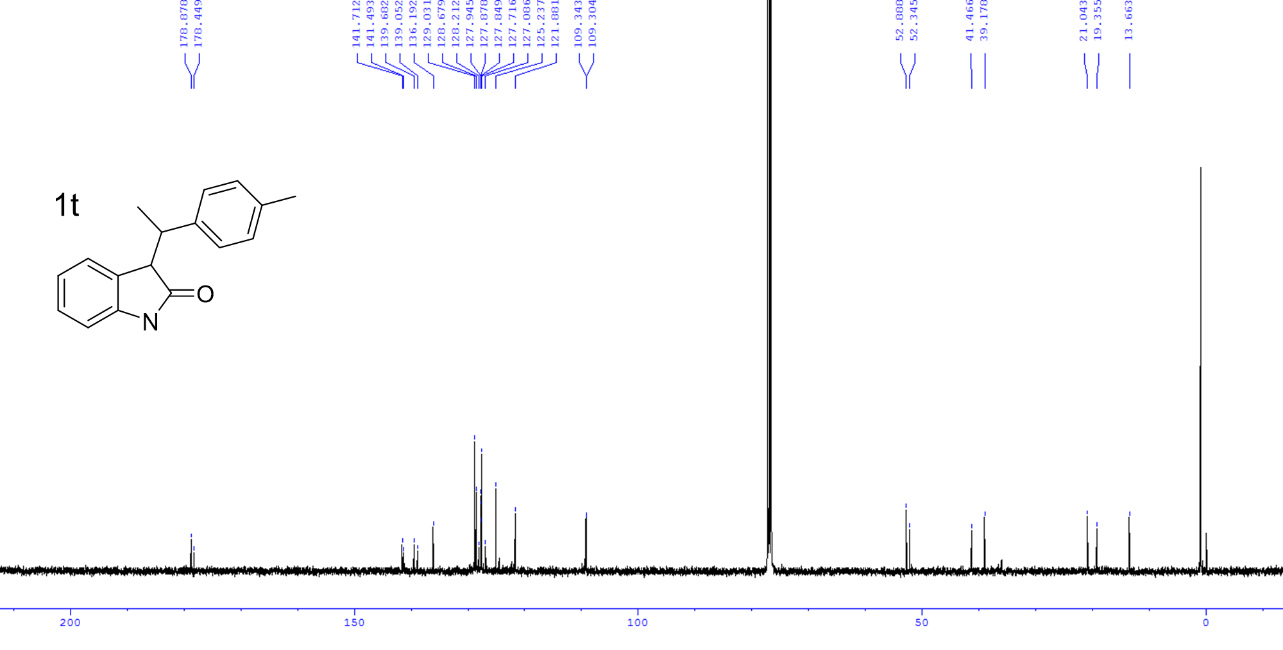
**

^13^C NMR spectrum of **1t**

**
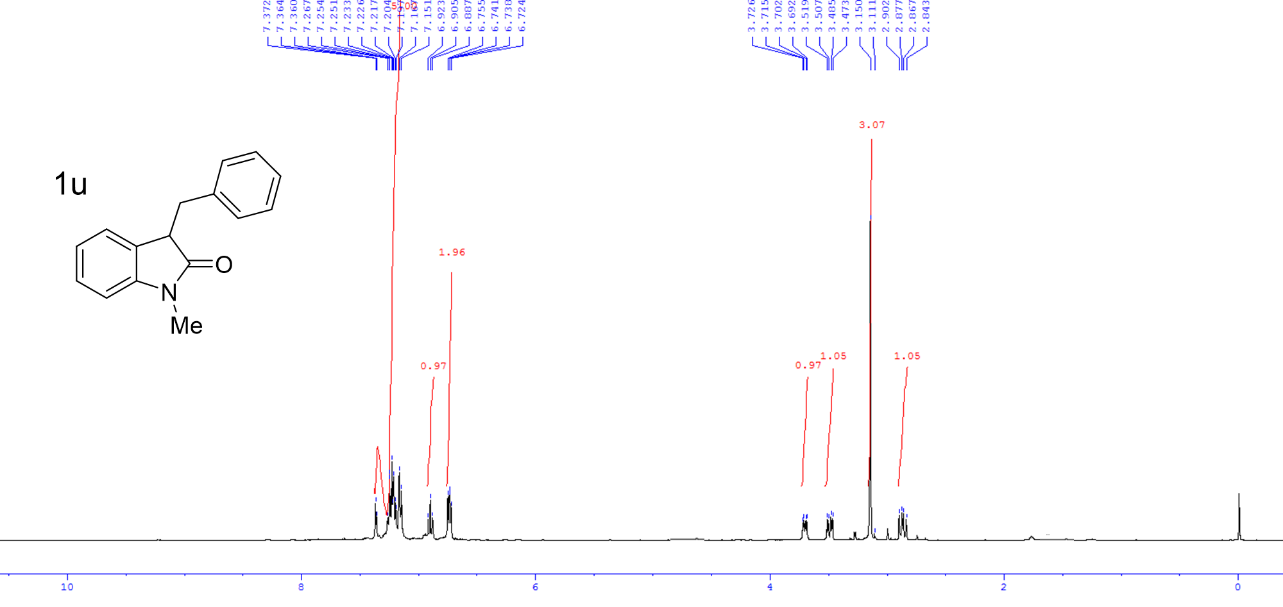
**

^1^H NMR spectrum of **1u**

**
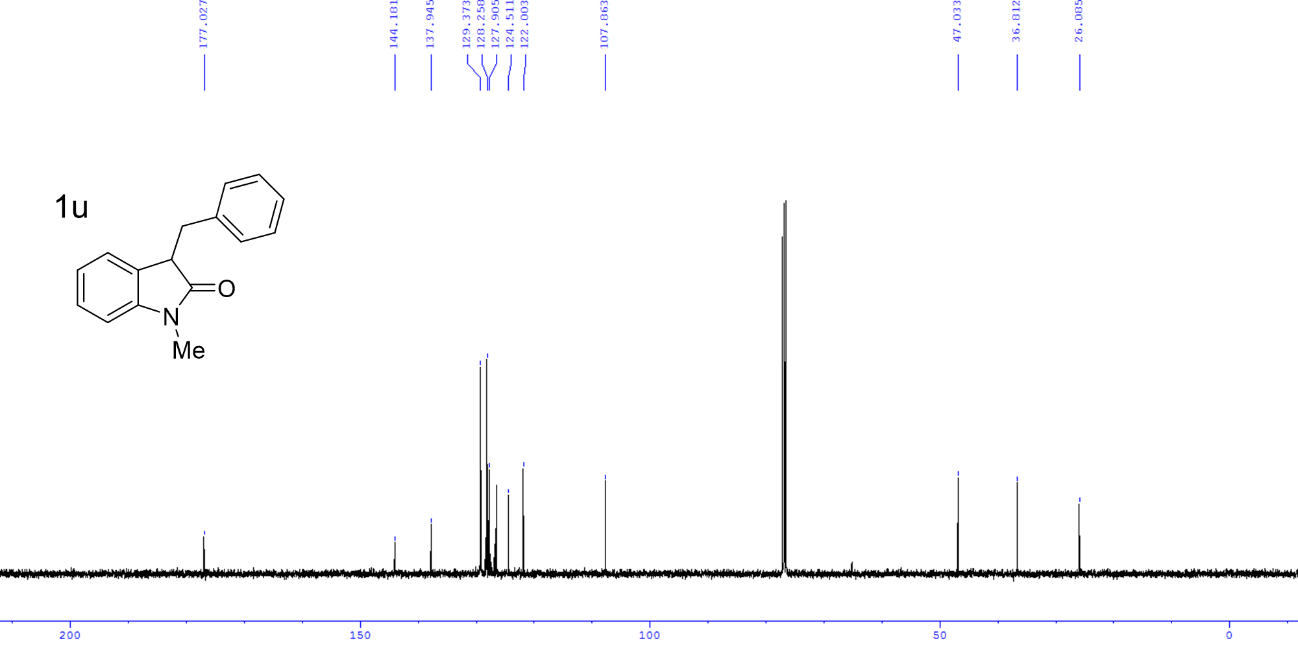
**

^13^C NMR spectrum of **1u**

**
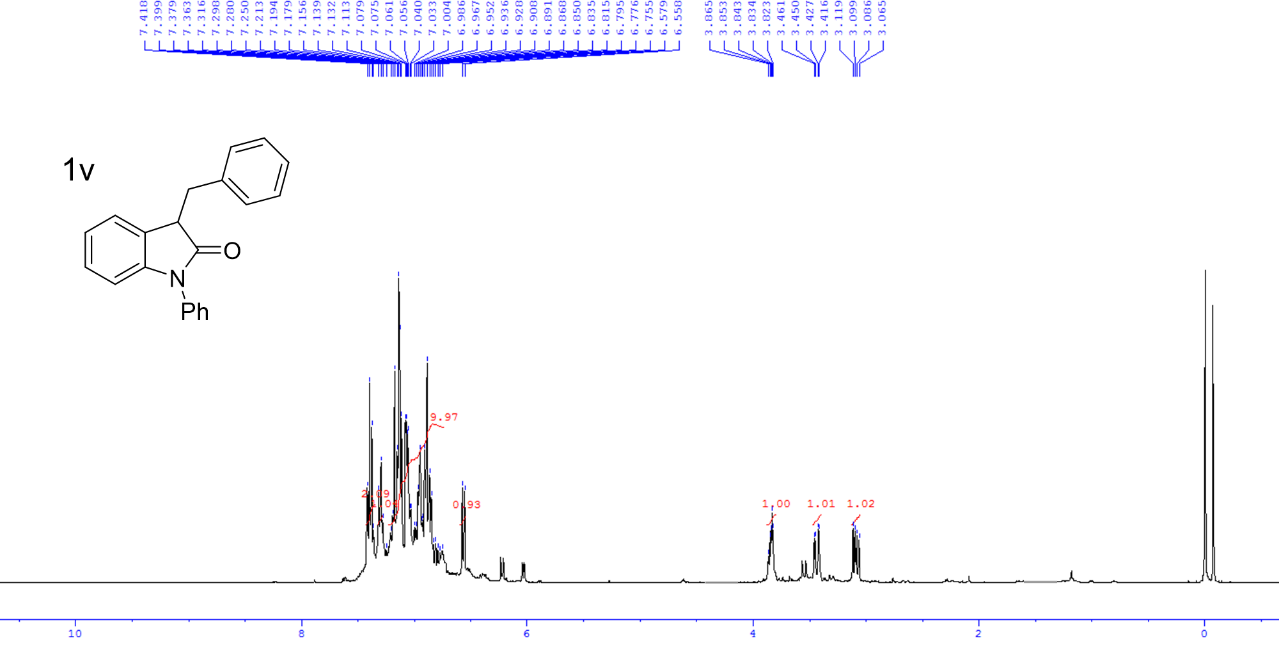
**

^1^H NMR spectrum of **1v**

**
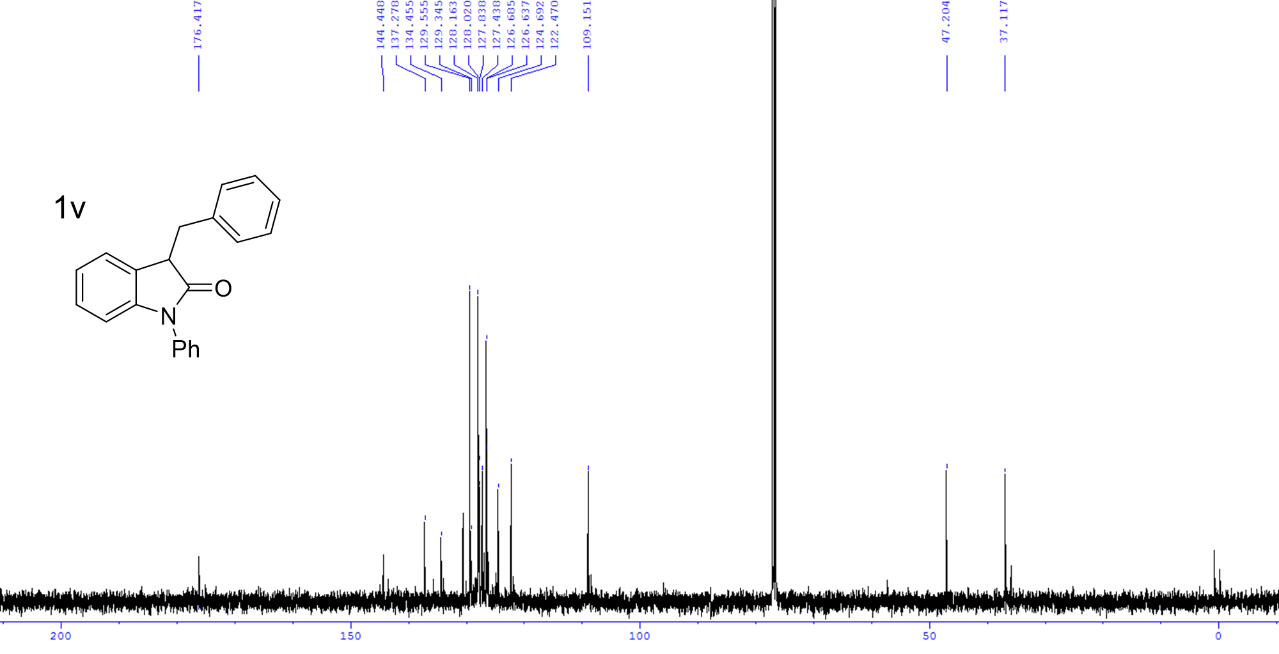
**

^13^C NMR spectrum of **1v**

**
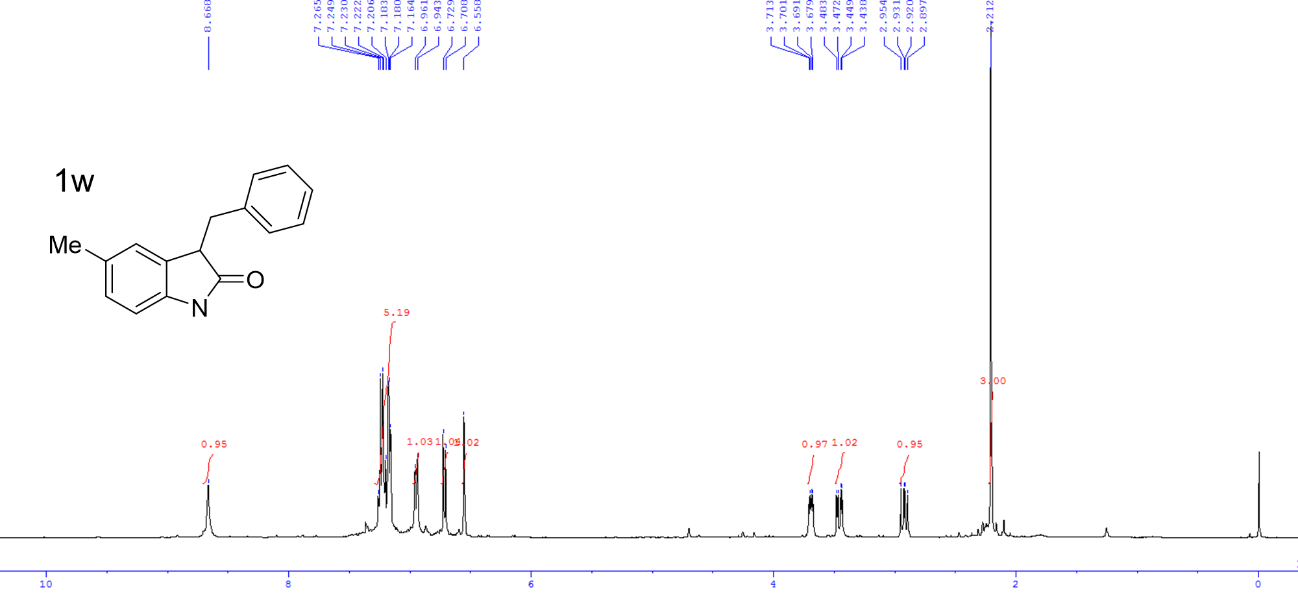
**

^1^H NMR spectrum of **1w**

**
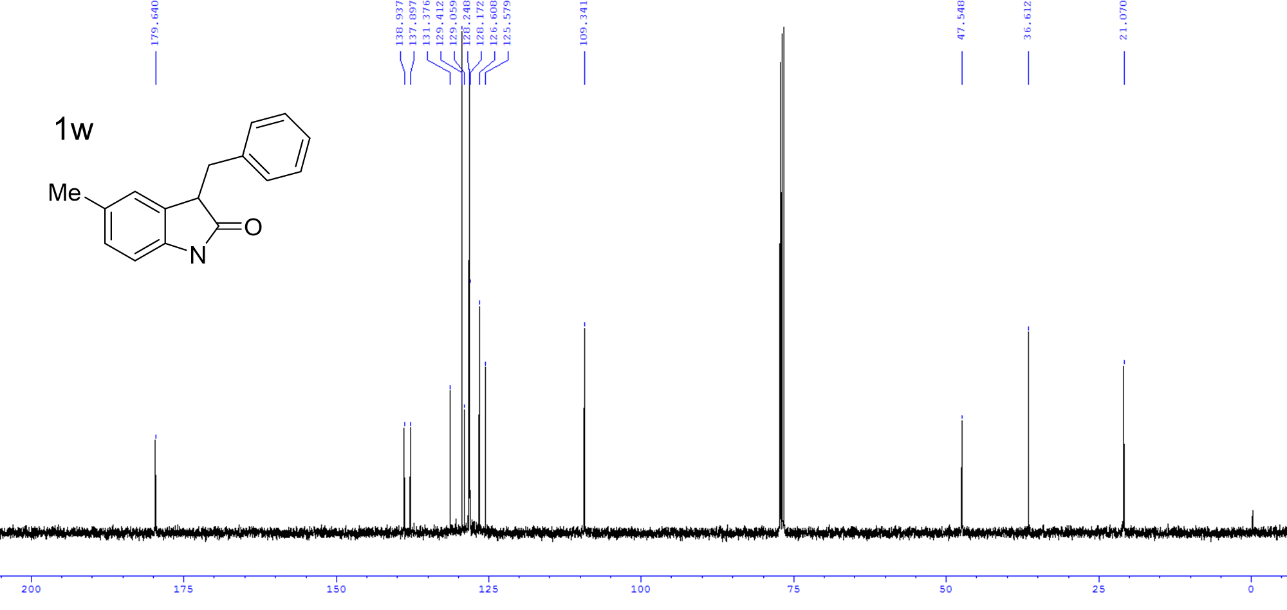
**

^13^C NMR spectrum of **1w**

**
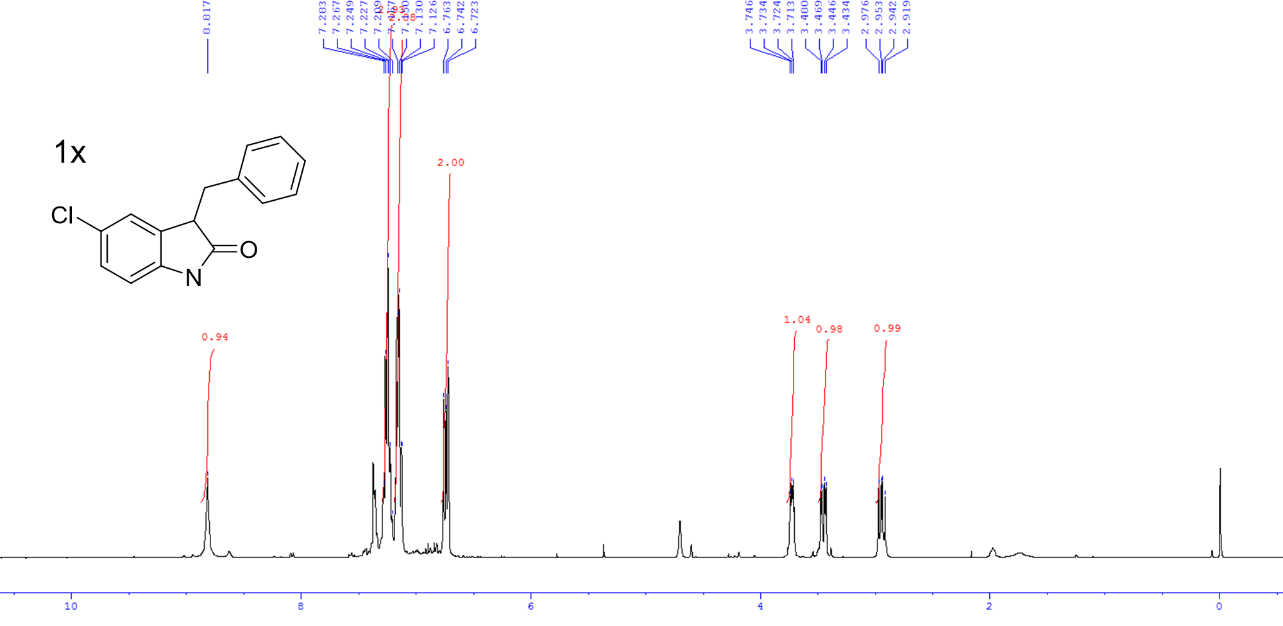
**

^1^H NMR spectrum of **1x**

**
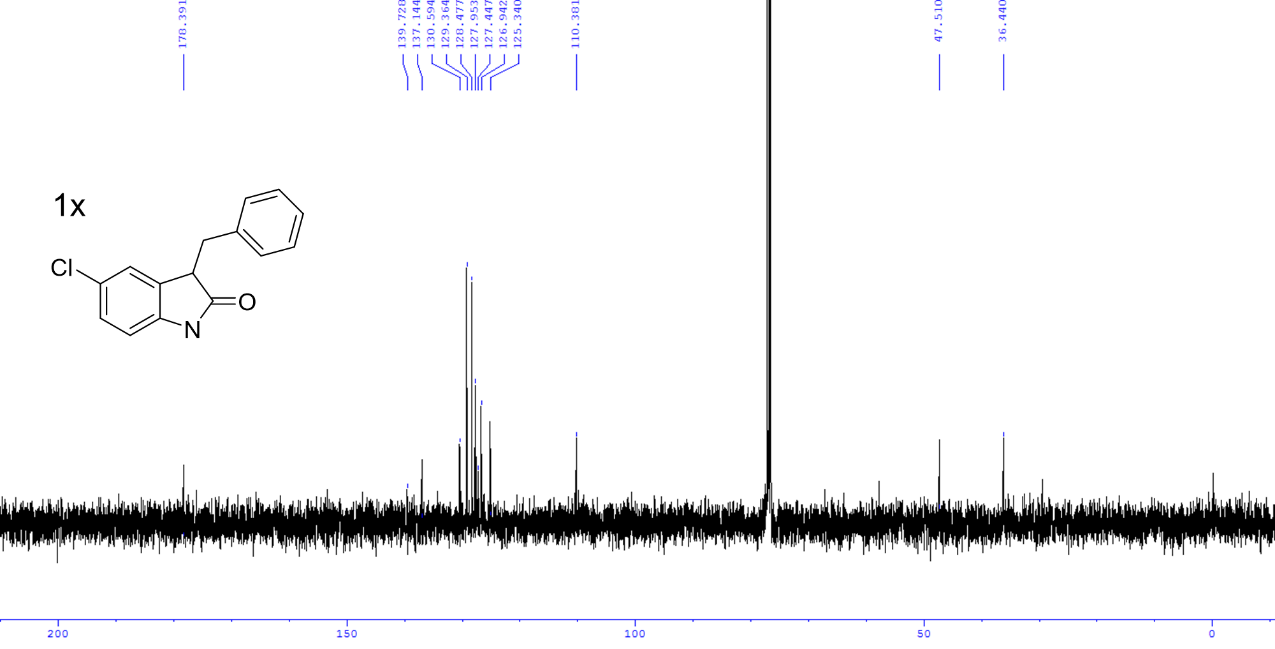
**

^13^C NMR spectrum of **1x**

^
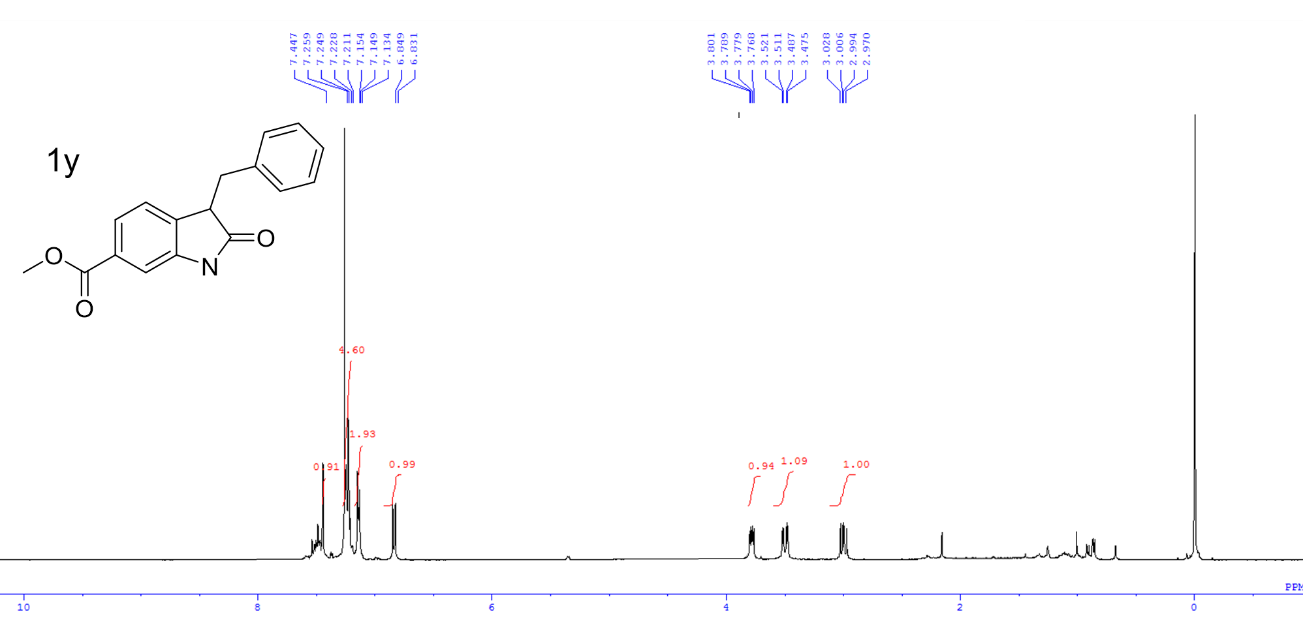
^

^1^H NMR spectrum of **1y**

**
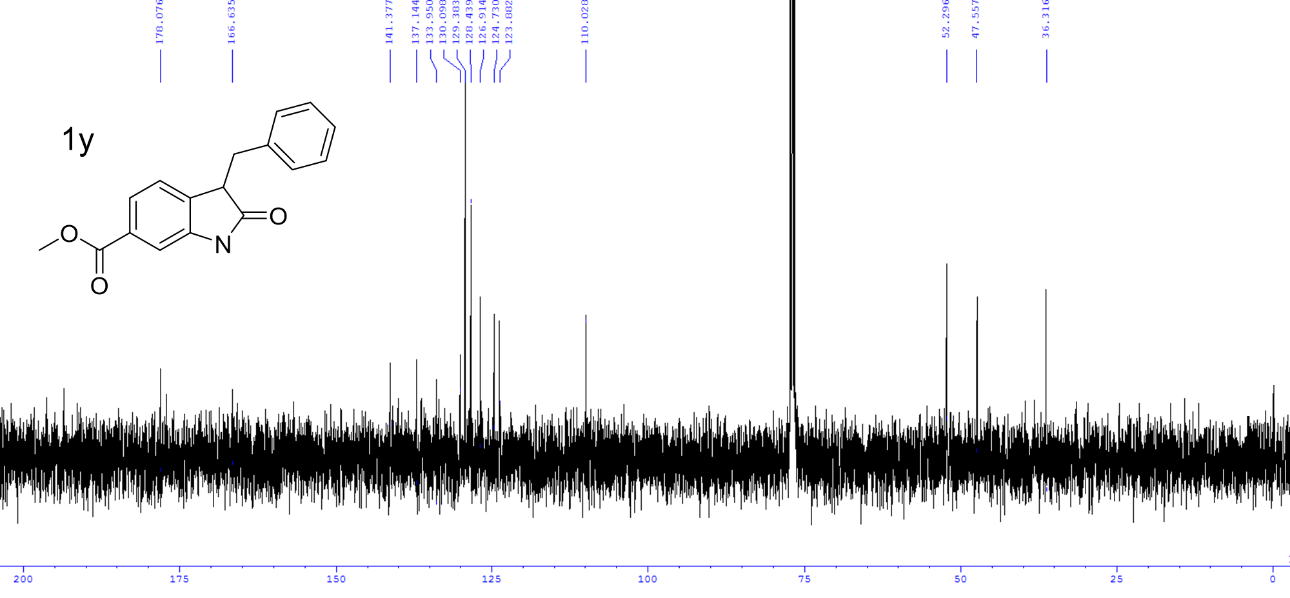
**

^13^C NMR spectrum of **1y**

**
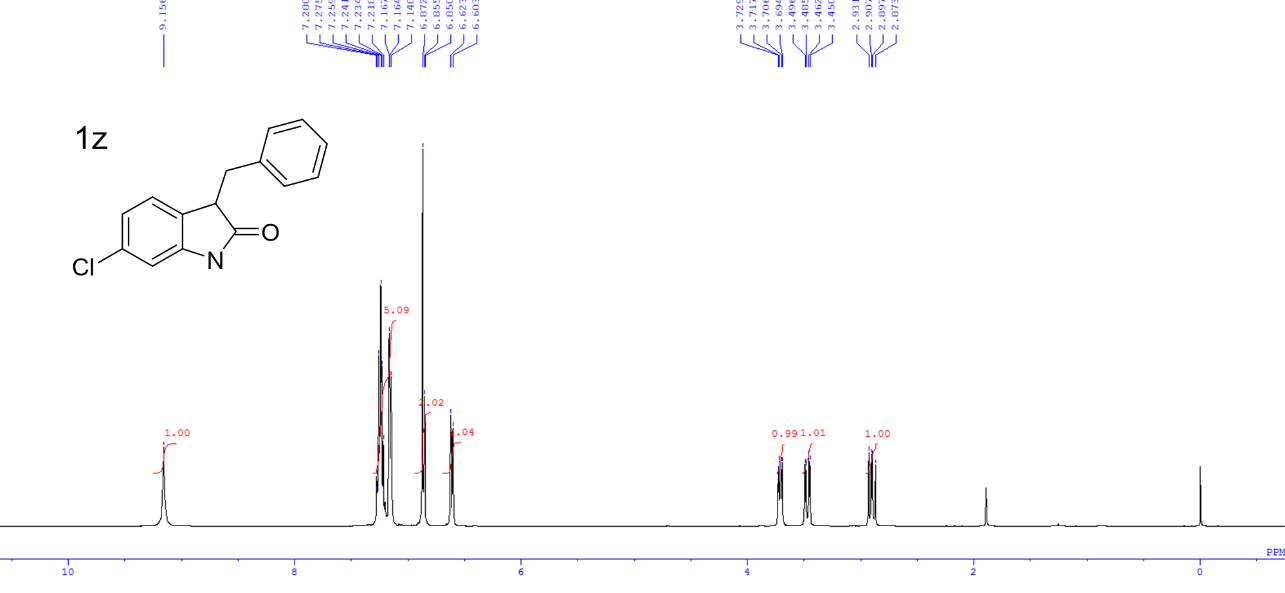
**

^1^H NMR spectrum of **1z**

**
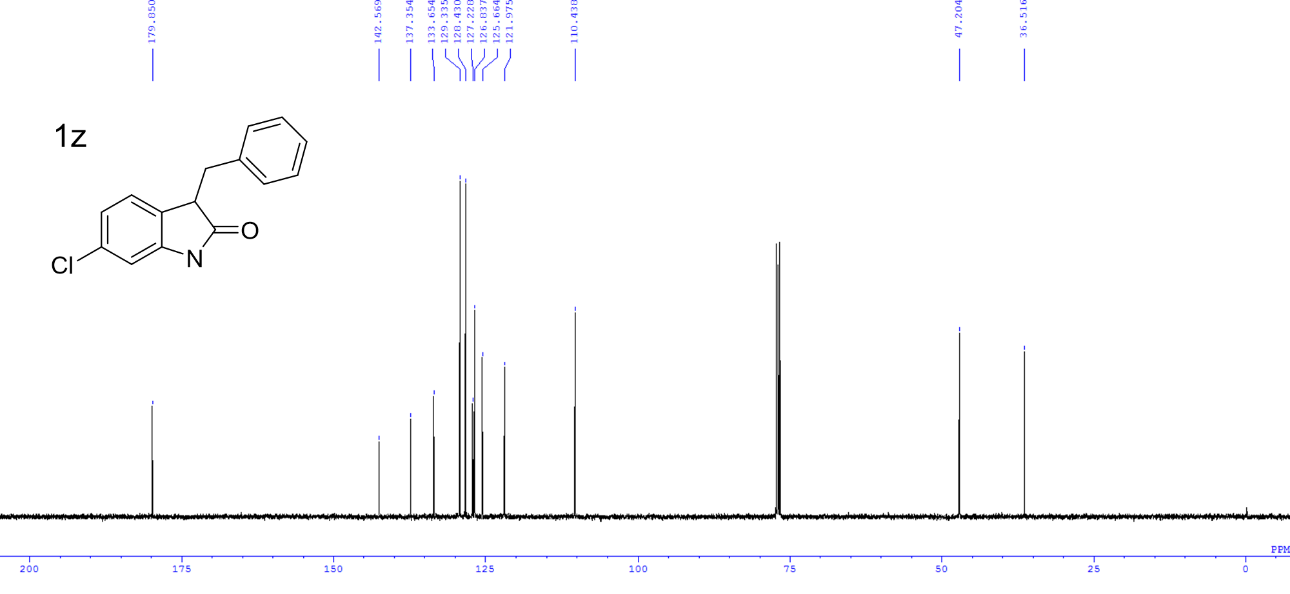
**

^13^C NMR spectrum of **1z**

**References**

1. Chaudhari, C. *et al.* C-3 alkylation of oxindole with alcohols by Pt/CeO_2_ catalyst in additive-free conditions. *Catal. Sci. Technol*. **4**, 1064–1069 (2014).

2. Liu, G. *et al.* C-3 alkylation of oxindole with alcohols catalyzed by an indene-functionalized mesoporous iridium catalyst. *Catal. Commun*. **12**, 655–659 (2011).

3. Putra, A. E., Oe. Y. & Ohta, T. Pd/C-catalyzed alkylation of heterocyclic nucleophiles with alcohols through the “borrowing hydrogen” process. *Eur. J. Org. Chem.* 7799–7805 (2015).

4. Volk, B., Mezei, T. & Simig, G. Raney Nickel induced 3-alkylation of oxindole with alcohols and diols. *Synthesis*, **5**, 595–597 (2002).

5. Jemsem, T. & Madsen, R. Ruthenium-catalyzed alkylation of oxindole with alcohols. *J. Org. Chem*. **74**, 3990–3992 (2009).

6. Cheng, L., Liu, L., Wang, D. & Chen, Y.-J. Highly enantioselective and organocatalytic α-amination of 2-oxindoles. *Org. Lett.* **11**, 3874–3877 (2009).

7. Bisht, G. S., Chaudhari, M. B., Gupte, V. S. & Gnanaprakasam, B. Ru-NHC catalyzed domino reaction of carbonyl compounds and alcohols: a short synthesis of donaxaridine. *ACS Omega* **2**, 8234–8252 (2017).

8. Andreani, A. *et al.* Synthesis and cardiotonic activity of 2-indolinones. *Eur. J. Med. Chem*. **25**, 187–190 (1990).

9. Grigg, R., Whitney, S., Sridharan, V., Keep, A. & Derrick, A. Iridium catalysed C-3 alkylation of oxindole with alcohols under solvent free thermal or microwave conditions. *Tetrahedron* **65**, 4375–4383 (2009).

10. Galzerano, P. Asymmetric iminium ion catalysis with a novel bifunctional primary amine thiourea: controlling adjacent quaternary and tertiary stereocenters. *Chem. Eur. J.* **15**, 7846–7849 (2009).

11. Liu, Y. *et al.* The employment of sodium hydride as a Michael donor in palladium-catalyzed reductions of α,β-unsaturated carbonyl compounds. *Adv. Synth. Catal.* **361**, 1554–1558 (2019).

12. Chakraborty, P., Garg, N., Manoury, E., Poli, R. & Sundararaju, B. C-alkylation of various carbonucleophiles with secondary alcohols under Co^III^-catalysis. *ACS Catal*. **10**, 8023–8031 (2020).

13. Schmid, J., Junge, T., Lang, J., Frey, W., & Peters, R. Polyfunctional bis-lewis-acid-/bis-triazolium catalysts for stereoselective 1,4-additions of 2-oxindoles to maleimides. *Angew. Chem., Int. Ed*. **58**, 5447−5451 (2019).
